# Supplementary material for: Phylogenomic and mitogenomic data can accelerate inventorying of tropical beetles during the current biodiversity crisis
Source: eLife. 2021 Dec 20;10:e71895. doi: 10.7554/eLife.71895 (PMC8798050; doi:10.7554/eLife.71895)
Supplement: Supplementary file 1. [file elife-71895-supp1.docx]

| Sample | #CON | #INF | #MIS |
| --- | --- | --- | --- |
| A00017 | 3 | 3 | 0 |
| A00018 | 3 | 3 | 0 |
| A00019 | 3 | 3 | 0 |
| A00020 | 3 | 1 | 2 |
| A00021 | 3 | 1 | 2 |
| A00022 | 3 | 2 | 1 |
| A00023 | 3 | 2 | 1 |
| A00024 | 3 | 3 | 0 |
| A00025 | 3 | 3 | 0 |
| A00026 | 3 | 2 | 1 |
| A00027 | 3 | 2 | 1 |
| A00028 | 3 | 1 | 2 |
| A00029 | 3 | 3 | 0 |
| A00030 | 3 | 3 | 0 |
| A00031 | 3 | 3 | 0 |
| A00031 | 3 | 3 | 0 |
| A00032 | 3 | 3 | 0 |
| A00032 | 3 | 3 | 0 |
| A00033 | 3 | 2 | 1 |
| A00034 | 3 | 3 | 0 |
| A00035 | 3 | 3 | 0 |
| A00036 | 3 | 3 | 0 |
| A00037 | 3 | 3 | 0 |
| A00038 | 3 | 3 | 0 |
| A00039 | 3 | 3 | 0 |
| A00040 | 3 | 3 | 0 |
| A00041 | 3 | 3 | 0 |
| A00041 | 3 | 3 | 0 |
| A00042 | 3 | 2 | 1 |
| A00043 | 3 | 3 | 0 |
| A00044 | 3 | 3 | 0 |
| A00045 | 3 | 3 | 0 |
| A00046 | 3 | 3 | 0 |
| A00048 | 3 | 2 | 1 |
| A00049 | 3 | 3 | 0 |
| A00050 | 3 | 2 | 1 |
| A00052 | 3 | 3 | 0 |
| A00053 | 3 | 3 | 0 |
| A00054 | 3 | 2 | 1 |
| A00055 | 3 | 3 | 0 |
| A00056 | 3 | 3 | 0 |
| A00057 | 3 | 2 | 1 |
| A00058 | 3 | 3 | 0 |
| A00059 | 3 | 3 | 0 |
| A00061 | 3 | 3 | 0 |
| A00062 | 3 | 3 | 0 |
| A00063 | 3 | 2 | 1 |
| A00064 | 3 | 2 | 1 |
| A00065 | 3 | 2 | 1 |
| A00066 | 3 | 3 | 0 |
| A00067 | 3 | 3 | 0 |
| A00068 | 3 | 3 | 0 |
| A00069 | 3 | 3 | 0 |
| A00070 | 3 | 3 | 0 |
| A00071 | 3 | 3 | 0 |
| A00072 | 3 | 2 | 1 |
| A00074 | 3 | 1 | 2 |
| A00075 | 3 | 3 | 0 |
| A00076 | 3 | 3 | 0 |
| A00077 | 3 | 3 | 0 |
| A00077 | 3 | 3 | 0 |
| A00078 | 3 | 3 | 0 |
| A00079 | 3 | 3 | 0 |
| A00080 | 3 | 2 | 1 |
| A00081 | 3 | 3 | 0 |
| A00082 | 3 | 2 | 1 |
| A00083 | 3 | 3 | 0 |
| A00084 | 3 | 3 | 0 |
| A00085 | 3 | 3 | 0 |
| A00086 | 3 | 3 | 0 |
| A00087 | 3 | 3 | 0 |
| A00088 | 3 | 3 | 0 |
| A00089 | 3 | 3 | 0 |
| A00090 | 3 | 3 | 0 |
| A00090 | 3 | 3 | 0 |
| A00091 | 3 | 3 | 0 |
| A00092 | 3 | 3 | 0 |
| A00093 | 3 | 3 | 0 |
| A00094 | 3 | 3 | 0 |
| A00095 | 3 | 2 | 1 |
| A00096 | 3 | 2 | 1 |
| A00097 | 3 | 3 | 0 |
| A00098 | 3 | 3 | 0 |
| A00099 | 3 | 3 | 0 |
| A00100 | 3 | 3 | 0 |
| A00101 | 3 | 1 | 2 |
| A00102 | 3 | 2 | 1 |
| A00103 | 3 | 1 | 2 |
| A00104 | 3 | 2 | 1 |
| A00105 | 3 | 2 | 1 |
| A00106 | 3 | 3 | 0 |
| A00107 | 3 | 1 | 2 |
| A00109 | 3 | 3 | 0 |
| A00110 | 3 | 2 | 1 |
| A00111 | 3 | 3 | 0 |
| A00112 | 3 | 3 | 0 |
| A00113 | 3 | 3 | 0 |
| A00114 | 3 | 2 | 1 |
| A00115 | 3 | 2 | 1 |
| A00116 | 3 | 2 | 1 |
| A00117 | 3 | 2 | 1 |
| A00118 | 3 | 3 | 0 |
| A00120 | 3 | 2 | 1 |
| A00121 | 3 | 3 | 0 |
| A00122 | 3 | 3 | 0 |
| A00123 | 3 | 3 | 0 |
| A00124 | 3 | 3 | 0 |
| A00125 | 3 | 3 | 0 |
| A00126 | 3 | 3 | 0 |
| A00127 | 3 | 3 | 0 |
| A00128 | 3 | 3 | 0 |
| A00129 | 3 | 2 | 1 |
| A00130 | 3 | 2 | 1 |
| A00131 | 3 | 1 | 2 |
| A00132 | 3 | 3 | 0 |
| A00133 | 3 | 2 | 1 |
| A00134 | 3 | 3 | 0 |
| A00136 | 3 | 3 | 0 |
| A00137 | 3 | 2 | 1 |
| A00138 | 3 | 3 | 0 |
| A00139 | 3 | 3 | 0 |
| A00140 | 3 | 3 | 0 |
| A00141 | 3 | 1 | 2 |
| A00142 | 3 | 3 | 0 |
| A00143 | 3 | 3 | 0 |
| A00144 | 3 | 3 | 0 |
| A00145 | 3 | 3 | 0 |
| A00146 | 3 | 3 | 0 |
| A00148 | 3 | 3 | 0 |
| A00148 | 3 | 3 | 0 |
| A00149 | 3 | 3 | 0 |
| A00152 | 3 | 2 | 1 |
| A00153 | 3 | 2 | 1 |
| A00154 | 3 | 3 | 0 |
| A00155 | 3 | 3 | 0 |
| A00156 | 3 | 3 | 0 |
| A00157 | 3 | 2 | 1 |
| A00158 | 3 | 3 | 0 |
| A00159 | 3 | 2 | 1 |
| A00161 | 3 | 2 | 1 |
| A00162 | 3 | 3 | 0 |
| A00163 | 3 | 3 | 0 |
| A00164 | 3 | 3 | 0 |
| A00165 | 3 | 3 | 0 |
| A00166 | 3 | 3 | 0 |
| A00167 | 3 | 3 | 0 |
| A00168 | 3 | 3 | 0 |
| A00169 | 3 | 3 | 0 |
| A00170 | 3 | 3 | 0 |
| A00171 | 3 | 3 | 0 |
| A00172 | 3 | 2 | 1 |
| A00173 | 3 | 3 | 0 |
| A00174 | 3 | 3 | 0 |
| A00175 | 3 | 3 | 0 |
| A00176 | 3 | 3 | 0 |
| A00178 | 3 | 3 | 0 |
| A00179 | 3 | 3 | 0 |
| A00180 | 3 | 3 | 0 |
| A00181 | 3 | 3 | 0 |
| A00182 | 3 | 2 | 1 |
| A00184 | 3 | 2 | 1 |
| A00185 | 3 | 2 | 1 |
| A00186 | 3 | 2 | 1 |
| A00187 | 3 | 3 | 0 |
| A00188 | 3 | 2 | 1 |
| A00189 | 3 | 3 | 0 |
| A00190 | 3 | 2 | 1 |
| A00192 | 3 | 2 | 1 |
| A00194 | 3 | 2 | 1 |
| A00195 | 3 | 3 | 0 |
| A00198 | 3 | 2 | 1 |
| A00199 | 3 | 3 | 0 |
| A00200 | 3 | 3 | 0 |
| A00201 | 3 | 3 | 0 |
| A00202 | 3 | 3 | 0 |
| A00203 | 3 | 3 | 0 |
| A00204 | 3 | 3 | 0 |
| A00205 | 3 | 3 | 0 |
| A00206 | 3 | 2 | 1 |
| A00207 | 3 | 1 | 2 |
| A00208 | 3 | 3 | 0 |
| A00209 | 3 | 3 | 0 |
| A00210 | 3 | 3 | 0 |
| A00211 | 3 | 3 | 0 |
| A00212 | 3 | 3 | 0 |
| A00213 | 3 | 3 | 0 |
| A00214 | 3 | 3 | 0 |
| A00215 | 3 | 3 | 0 |
| A00217 | 3 | 3 | 0 |
| A00219 | 3 | 3 | 0 |
| A00220 | 3 | 3 | 0 |
| A00221 | 3 | 3 | 0 |
| A00222 | 3 | 3 | 0 |
| A00223 | 3 | 3 | 0 |
| A00225 | 3 | 3 | 0 |
| A00227 | 3 | 3 | 0 |
| A00228 | 3 | 3 | 0 |
| A00229 | 3 | 3 | 0 |
| A00232 | 3 | 3 | 0 |
| A00235 | 3 | 3 | 0 |
| A00236 | 3 | 3 | 0 |
| A00237 | 3 | 3 | 0 |
| A00238 | 3 | 3 | 0 |
| A00239 | 3 | 3 | 0 |
| A00240 | 3 | 3 | 0 |
| A00241 | 3 | 3 | 0 |
| A00242 | 3 | 3 | 0 |
| A00243 | 3 | 3 | 0 |
| A00247 | 3 | 3 | 0 |
| A00248 | 3 | 3 | 0 |
| A00249 | 3 | 3 | 0 |
| A00251 | 3 | 3 | 0 |
| A00252 | 3 | 3 | 0 |
| A00253 | 3 | 3 | 0 |
| A00255 | 3 | 3 | 0 |
| A00256 | 3 | 3 | 0 |
| A00257 | 3 | 3 | 0 |
| A00258 | 3 | 3 | 0 |
| A00259 | 3 | 3 | 0 |
| A00260 | 3 | 3 | 0 |
| A00261 | 3 | 3 | 0 |
| A00262 | 3 | 3 | 0 |
| A00263 | 3 | 3 | 0 |
| A00265 | 3 | 2 | 1 |
| A00266 | 3 | 3 | 0 |
| A00267 | 3 | 3 | 0 |
| A00268 | 3 | 3 | 0 |
| A00269 | 3 | 3 | 0 |
| A00270 | 3 | 3 | 0 |
| A00271 | 3 | 3 | 0 |
| A00273 | 3 | 3 | 0 |
| A00274 | 3 | 2 | 1 |
| A00275 | 3 | 3 | 0 |
| A00276 | 3 | 3 | 0 |
| A00277 | 3 | 3 | 0 |
| A00278 | 3 | 3 | 0 |
| A00279 | 3 | 2 | 1 |
| A00280 | 3 | 3 | 0 |
| A00281 | 3 | 3 | 0 |
| A00282 | 3 | 3 | 0 |
| A00283 | 3 | 3 | 0 |
| A00284 | 3 | 3 | 0 |
| A00285 | 3 | 3 | 0 |
| A00286 | 3 | 3 | 0 |
| A00287 | 3 | 3 | 0 |
| A00289 | 3 | 3 | 0 |
| A00290 | 3 | 3 | 0 |
| A00291 | 3 | 3 | 0 |
| A00292 | 3 | 2 | 1 |
| A00296 | 3 | 3 | 0 |
| A00297 | 3 | 3 | 0 |
| A00298 | 3 | 3 | 0 |
| A00299 | 3 | 3 | 0 |
| A00300 | 3 | 3 | 0 |
| A00301 | 3 | 3 | 0 |
| A00304 | 3 | 3 | 0 |
| A00305 | 3 | 3 | 0 |
| A00306 | 3 | 3 | 0 |
| A00307 | 3 | 3 | 0 |
| A00308 | 3 | 1 | 2 |
| A00311 | 3 | 3 | 0 |
| A00312 | 3 | 3 | 0 |
| A00313 | 3 | 3 | 0 |
| A00314 | 3 | 3 | 0 |
| A00315 | 3 | 3 | 0 |
| A00316 | 3 | 3 | 0 |
| A00317 | 3 | 3 | 0 |
| A00318 | 3 | 3 | 0 |
| A00319 | 3 | 3 | 0 |
| A00321 | 3 | 3 | 0 |
| A00322 | 3 | 3 | 0 |
| A00326 | 3 | 2 | 1 |
| A00327 | 3 | 3 | 0 |
| A00329 | 3 | 3 | 0 |
| A00330 | 3 | 3 | 0 |
| A00331 | 3 | 3 | 0 |
| A00332 | 3 | 3 | 0 |
| A00333 | 3 | 3 | 0 |
| A00334 | 3 | 3 | 0 |
| A00335 | 3 | 3 | 0 |
| A00336 | 3 | 3 | 0 |
| A00337 | 3 | 2 | 1 |
| A00339 | 3 | 3 | 0 |
| A00340 | 3 | 2 | 1 |
| A00341 | 3 | 3 | 0 |
| A00342 | 3 | 3 | 0 |
| A00343 | 3 | 3 | 0 |
| A00344 | 3 | 3 | 0 |
| A00347 | 3 | 3 | 0 |
| A00348 | 3 | 3 | 0 |
| A00349 | 3 | 3 | 0 |
| A00350 | 3 | 3 | 0 |
| A00351 | 3 | 3 | 0 |
| A00352 | 3 | 2 | 1 |
| A00353 | 3 | 3 | 0 |
| A00355 | 3 | 3 | 0 |
| A00356 | 3 | 2 | 1 |
| A00357 | 3 | 3 | 0 |
| A00358 | 3 | 3 | 0 |
| A00359 | 3 | 3 | 0 |
| A00360 | 3 | 3 | 0 |
| A00361 | 3 | 3 | 0 |
| A00362 | 3 | 3 | 0 |
| A00363 | 3 | 3 | 0 |
| A00364 | 3 | 3 | 0 |
| A00365 | 3 | 2 | 1 |
| A00366 | 3 | 3 | 0 |
| A00367 | 3 | 3 | 0 |
| A00368 | 3 | 3 | 0 |
| A00369 | 3 | 3 | 0 |
| A00370 | 3 | 3 | 0 |
| A00371 | 3 | 3 | 0 |
| A00372 | 3 | 3 | 0 |
| A00373 | 3 | 3 | 0 |
| A00374 | 3 | 3 | 0 |
| A00375 | 3 | 3 | 0 |
| A00376 | 3 | 3 | 0 |
| A00377 | 3 | 3 | 0 |
| A00378 | 3 | 3 | 0 |
| A00379 | 3 | 3 | 0 |
| A00380 | 3 | 3 | 0 |
| A00381 | 3 | 3 | 0 |
| A00382 | 3 | 3 | 0 |
| A00383 | 3 | 3 | 0 |
| A00384 | 3 | 3 | 0 |
| A00385 | 3 | 3 | 0 |
| A00386 | 3 | 3 | 0 |
| A00387 | 3 | 3 | 0 |
| A00389 | 3 | 3 | 0 |
| A00390 | 3 | 3 | 0 |
| A00391 | 3 | 3 | 0 |
| A00393 | 3 | 3 | 0 |
| A00394 | 3 | 3 | 0 |
| A00395 | 3 | 2 | 1 |
| A00396 | 3 | 3 | 0 |
| A00397 | 3 | 3 | 0 |
| A00399 | 3 | 3 | 0 |
| A00400 | 3 | 3 | 0 |
| A00401 | 3 | 3 | 0 |
| A00402 | 3 | 3 | 0 |
| A00403 | 3 | 3 | 0 |
| A00404 | 3 | 2 | 1 |
| A00405 | 3 | 2 | 1 |
| A00406 | 3 | 2 | 1 |
| A00407 | 3 | 3 | 0 |
| A00408 | 3 | 2 | 1 |
| A00409 | 3 | 3 | 0 |
| A00410 | 3 | 3 | 0 |
| A00411 | 3 | 2 | 1 |
| A00412 | 3 | 2 | 1 |
| A00413 | 3 | 3 | 0 |
| A00414 | 3 | 3 | 0 |
| A00415 | 3 | 3 | 0 |
| A00416 | 3 | 3 | 0 |
| A00417 | 3 | 3 | 0 |
| A00418 | 3 | 3 | 0 |
| A00419 | 3 | 3 | 0 |
| A00420 | 3 | 3 | 0 |
| A00421 | 3 | 3 | 0 |
| A00422 | 3 | 3 | 0 |
| A00423 | 3 | 3 | 0 |
| A00424 | 3 | 3 | 0 |
| A00425 | 3 | 3 | 0 |
| A00426 | 3 | 3 | 0 |
| A00427 | 3 | 3 | 0 |
| A00428 | 3 | 3 | 0 |
| A00429 | 3 | 3 | 0 |
| A00430 | 3 | 3 | 0 |
| A00431 | 3 | 3 | 0 |
| A00432 | 3 | 3 | 0 |
| A00433 | 3 | 3 | 0 |
| A00434 | 3 | 3 | 0 |
| A00435 | 3 | 3 | 0 |
| A00437 | 3 | 2 | 1 |
| A00438 | 3 | 3 | 0 |
| A00439 | 3 | 2 | 1 |
| A00440 | 3 | 3 | 0 |
| A00441 | 3 | 3 | 0 |
| A00442 | 3 | 3 | 0 |
| A00443 | 3 | 3 | 0 |
| A00444 | 3 | 3 | 0 |
| A00445 | 3 | 3 | 0 |
| A00446 | 3 | 3 | 0 |
| A00447 | 3 | 3 | 0 |
| A00448 | 3 | 3 | 0 |
| A00449 | 3 | 3 | 0 |
| A00450 | 3 | 3 | 0 |
| A00451 | 3 | 3 | 0 |
| A00452 | 3 | 2 | 1 |
| A00453 | 3 | 2 | 1 |
| A00454 | 3 | 3 | 0 |
| A00455 | 3 | 3 | 0 |
| A00456 | 3 | 3 | 0 |
| A00457 | 3 | 3 | 0 |
| A00458 | 3 | 3 | 0 |
| A00459 | 3 | 3 | 0 |
| A00652 | 3 | 2 | 1 |
| A00653 | 3 | 2 | 1 |
| A00655 | 3 | 2 | 1 |
| A00777 | 3 | 2 | 1 |
| A00778 | 3 | 3 | 0 |
| A00779 | 3 | 3 | 0 |
| A00780 | 3 | 1 | 2 |
| A00781 | 3 | 1 | 2 |
| A00782 | 3 | 1 | 2 |
| A00783 | 3 | 3 | 0 |
| A00784 | 3 | 1 | 2 |
| A00785 | 3 | 3 | 0 |
| A00786 | 3 | 1 | 2 |
| A00787 | 3 | 1 | 2 |
| A00788 | 3 | 1 | 2 |
| A00790 | 3 | 3 | 0 |
| A00791 | 3 | 1 | 2 |
| A00793 | 3 | 1 | 2 |
| A00794 | 3 | 1 | 2 |
| A00795 | 3 | 1 | 2 |
| A00797 | 3 | 1 | 2 |
| A00798 | 3 | 1 | 2 |
| A00799 | 3 | 3 | 0 |
| A00800 | 3 | 1 | 2 |
| A00801 | 3 | 1 | 2 |
| A00802 | 3 | 1 | 2 |
| A00803 | 3 | 1 | 2 |
| A00804 | 3 | 1 | 2 |
| A00805 | 3 | 1 | 2 |
| A00806 | 3 | 1 | 2 |
| A00809 | 3 | 1 | 2 |
| A00810 | 3 | 1 | 2 |
| A00811 | 3 | 1 | 2 |
| A00812 | 3 | 1 | 2 |
| A00813 | 3 | 1 | 2 |
| A00814 | 3 | 1 | 2 |
| A00815 | 3 | 3 | 0 |
| A00816 | 3 | 1 | 2 |
| A00817 | 3 | 1 | 2 |
| A00819 | 3 | 2 | 1 |
| A00820 | 3 | 1 | 2 |
| A00821 | 3 | 3 | 0 |
| A00822 | 3 | 1 | 2 |
| A00823 | 3 | 1 | 2 |
| A00824 | 3 | 3 | 0 |
| A00825 | 3 | 1 | 2 |
| A00826 | 3 | 1 | 2 |
| A00827 | 3 | 1 | 2 |
| A00828 | 3 | 3 | 0 |
| A00829 | 3 | 2 | 1 |
| A00830 | 3 | 1 | 2 |
| A00832 | 3 | 1 | 2 |
| A00833 | 3 | 1 | 2 |
| A00834 | 3 | 1 | 2 |
| A00835 | 3 | 1 | 2 |
| A00836 | 3 | 1 | 2 |
| A00837 | 3 | 1 | 2 |
| A00838 | 3 | 1 | 2 |
| A00839 | 3 | 3 | 0 |
| A00840 | 3 | 1 | 2 |
| A00841 | 3 | 1 | 2 |
| A00842 | 3 | 1 | 2 |
| A00843 | 3 | 3 | 0 |
| A00844 | 3 | 3 | 0 |
| A00846 | 3 | 2 | 1 |
| A00848 | 3 | 1 | 2 |
| A00849 | 3 | 3 | 0 |
| A00850 | 3 | 3 | 0 |
| A00851 | 3 | 3 | 0 |
| A00852 | 3 | 1 | 2 |
| A00855 | 3 | 2 | 1 |
| A00856 | 3 | 2 | 1 |
| A00857 | 3 | 1 | 2 |
| A00859 | 3 | 2 | 1 |
| A00860 | 3 | 2 | 1 |
| A00861 | 3 | 2 | 1 |
| A00862 | 3 | 3 | 0 |
| A00863 | 3 | 3 | 0 |
| A00865 | 3 | 1 | 2 |
| A00867 | 3 | 1 | 2 |
| A00868 | 3 | 1 | 2 |
| A00869 | 3 | 2 | 1 |
| A00870 | 3 | 1 | 2 |
| A00872 | 3 | 1 | 2 |
| A00873 | 3 | 1 | 2 |
| A00874 | 3 | 1 | 2 |
| A00876 | 3 | 1 | 2 |
| A00877 | 3 | 1 | 2 |
| A00878 | 3 | 3 | 0 |
| A00879 | 3 | 3 | 0 |
| A00880 | 3 | 1 | 2 |
| A00881 | 3 | 1 | 2 |
| A00882 | 3 | 2 | 1 |
| A00883 | 3 | 2 | 1 |
| A00884 | 3 | 1 | 2 |
| A00885 | 3 | 3 | 0 |
| A00887 | 3 | 1 | 2 |
| A00888 | 3 | 2 | 1 |
| A00889 | 3 | 1 | 2 |
| A00890 | 3 | 1 | 2 |
| A00891 | 3 | 3 | 0 |
| A00892 | 3 | 1 | 2 |
| A00893 | 3 | 1 | 2 |
| A00894 | 3 | 1 | 2 |
| A00896 | 3 | 3 | 0 |
| A00897 | 3 | 2 | 1 |
| A00898 | 3 | 3 | 0 |
| A00899 | 3 | 1 | 2 |
| A00900 | 3 | 1 | 2 |
| A00901 | 3 | 2 | 1 |
| A00902 | 3 | 1 | 2 |
| A00903 | 3 | 1 | 2 |
| A00904 | 3 | 1 | 2 |
| A00905 | 3 | 1 | 2 |
| A00906 | 3 | 3 | 0 |
| A00907 | 3 | 1 | 2 |
| A00909 | 3 | 3 | 0 |
| A00910 | 3 | 3 | 0 |
| A00911 | 3 | 1 | 2 |
| A00912 | 3 | 3 | 0 |
| A00913 | 3 | 1 | 2 |
| A00914 | 3 | 1 | 2 |
| A00915 | 3 | 1 | 2 |
| A00916 | 3 | 1 | 2 |
| A00917 | 3 | 1 | 2 |
| A00918 | 3 | 1 | 2 |
| A00919 | 3 | 1 | 2 |
| A00920 | 3 | 1 | 2 |
| A00921 | 3 | 1 | 2 |
| A00922 | 3 | 3 | 0 |
| A00923 | 3 | 1 | 2 |
| A00924 | 3 | 3 | 0 |
| A00925 | 3 | 1 | 2 |
| A00926 | 3 | 1 | 2 |
| A00928 | 3 | 1 | 2 |
| A00929 | 3 | 1 | 2 |
| A00930 | 3 | 2 | 1 |
| A00931 | 3 | 1 | 2 |
| A00932 | 3 | 1 | 2 |
| A00933 | 3 | 3 | 0 |
| A00934 | 3 | 1 | 2 |
| A00935 | 3 | 1 | 2 |
| A00936 | 3 | 1 | 2 |
| A00937 | 3 | 1 | 2 |
| A00938 | 3 | 1 | 2 |
| A00940 | 3 | 1 | 2 |
| A00941 | 3 | 2 | 1 |
| A00942 | 3 | 3 | 0 |
| A00943 | 3 | 1 | 2 |
| A00944 | 3 | 1 | 2 |
| A00945 | 3 | 1 | 2 |
| A00946 | 3 | 1 | 2 |
| A00947 | 3 | 2 | 1 |
| A00948 | 3 | 2 | 1 |
| A00949 | 3 | 3 | 0 |
| A00950 | 3 | 2 | 1 |
| A00951 | 3 | 1 | 2 |
| A00952 | 3 | 3 | 0 |
| A00953 | 3 | 1 | 2 |
| A00954 | 3 | 2 | 1 |
| A00955 | 3 | 2 | 1 |
| A00956 | 3 | 2 | 1 |
| A00957 | 3 | 2 | 1 |
| A00958 | 3 | 2 | 1 |
| A00959 | 3 | 2 | 1 |
| A00960 | 3 | 2 | 1 |
| A00961 | 3 | 1 | 2 |
| A00962 | 3 | 2 | 1 |
| A00963 | 3 | 2 | 1 |
| A00964 | 3 | 3 | 0 |
| A00965 | 3 | 1 | 2 |
| A00966 | 3 | 1 | 2 |
| A00967 | 3 | 1 | 2 |
| A00968 | 3 | 1 | 2 |
| A00969 | 3 | 1 | 2 |
| A00970 | 3 | 3 | 0 |
| A00971 | 3 | 3 | 0 |
| A00972 | 3 | 1 | 2 |
| A00973 | 3 | 1 | 2 |
| A00974 | 3 | 3 | 0 |
| A00975 | 3 | 3 | 0 |
| A00976 | 3 | 3 | 0 |
| A00977 | 3 | 1 | 2 |
| A00978 | 3 | 1 | 2 |
| A00979 | 3 | 3 | 0 |
| A00980 | 3 | 1 | 2 |
| A00981 | 3 | 1 | 2 |
| A00982 | 3 | 3 | 0 |
| A00983 | 3 | 2 | 1 |
| A00984 | 3 | 3 | 0 |
| A00985 | 3 | 2 | 1 |
| A00986 | 3 | 2 | 1 |
| A00987 | 3 | 2 | 1 |
| A00988 | 3 | 2 | 1 |
| A00989 | 3 | 2 | 1 |
| A00990 | 3 | 3 | 0 |
| A00991 | 3 | 3 | 0 |
| A00992 | 3 | 3 | 0 |
| A00993 | 3 | 3 | 0 |
| A00994 | 3 | 1 | 2 |
| A00995 | 3 | 1 | 2 |
| A00996 | 3 | 3 | 0 |
| A00998 | 3 | 3 | 0 |
| A00999 | 3 | 2 | 1 |
| A01000 | 3 | 3 | 0 |
| A01001 | 3 | 3 | 0 |
| A01002 | 3 | 3 | 0 |
| A01003 | 3 | 3 | 0 |
| A01004 | 3 | 3 | 0 |
| A01005 | 3 | 3 | 0 |
| A01006 | 3 | 2 | 1 |
| A01007 | 3 | 3 | 0 |
| A01008 | 3 | 3 | 0 |
| A01009 | 3 | 1 | 2 |
| A01010 | 3 | 3 | 0 |
| A01011 | 3 | 3 | 0 |
| A01012 | 3 | 3 | 0 |
| A01013 | 3 | 1 | 2 |
| A01014 | 3 | 1 | 2 |
| A01015 | 3 | 2 | 1 |
| A01016 | 3 | 2 | 1 |
| A01017 | 3 | 3 | 0 |
| A01018 | 3 | 1 | 2 |
| A01019 | 3 | 3 | 0 |
| A01020 | 3 | 3 | 0 |
| A01021 | 3 | 1 | 2 |
| A01022 | 3 | 1 | 2 |
| A01023 | 3 | 1 | 2 |
| A01024 | 3 | 1 | 2 |
| A01025 | 3 | 3 | 0 |
| A01026 | 3 | 3 | 0 |
| A01027 | 3 | 1 | 2 |
| A01028 | 3 | 3 | 0 |
| A01029 | 3 | 1 | 2 |
| A01030 | 3 | 3 | 0 |
| A01031 | 3 | 1 | 2 |
| A01032 | 3 | 1 | 2 |
| A01034 | 3 | 1 | 2 |
| A01035 | 3 | 3 | 0 |
| A01038 | 3 | 1 | 2 |
| A01039 | 3 | 1 | 2 |
| A01040 | 3 | 3 | 0 |
| A01041 | 3 | 2 | 1 |
| A01042 | 3 | 3 | 0 |
| A01043 | 3 | 1 | 2 |
| A01044 | 3 | 2 | 1 |
| A01045 | 3 | 1 | 2 |
| A01046 | 3 | 1 | 2 |
| A01047 | 3 | 1 | 2 |
| A01049 | 3 | 1 | 2 |
| A01051 | 3 | 1 | 2 |
| A01052 | 3 | 3 | 0 |
| A01053 | 3 | 2 | 1 |
| A01054 | 3 | 2 | 1 |
| A01055 | 3 | 2 | 1 |
| A01056 | 3 | 2 | 1 |
| A01057 | 3 | 1 | 2 |
| A01058 | 3 | 1 | 2 |
| A01063 | 3 | 3 | 0 |
| A01064 | 3 | 1 | 2 |
| A01065 | 3 | 2 | 1 |
| A01066 | 3 | 3 | 0 |
| A01067 | 3 | 1 | 2 |
| A01068 | 3 | 1 | 2 |
| A01069 | 3 | 1 | 2 |
| A01070 | 3 | 3 | 0 |
| A01071 | 3 | 1 | 2 |
| A01072 | 3 | 1 | 2 |
| A01073 | 3 | 3 | 0 |
| A01074 | 3 | 1 | 2 |
| A01075 | 3 | 1 | 2 |
| A01076 | 3 | 1 | 2 |
| A01077 | 3 | 3 | 0 |
| A01078 | 3 | 1 | 2 |
| A01079 | 3 | 3 | 0 |
| A01080 | 3 | 1 | 2 |
| A01081 | 3 | 2 | 1 |
| A01082 | 3 | 2 | 1 |
| A01083 | 3 | 3 | 0 |
| A01084 | 3 | 3 | 0 |
| A01085 | 3 | 1 | 2 |
| A01086 | 3 | 1 | 2 |
| A01087 | 3 | 3 | 0 |
| A01088 | 3 | 3 | 0 |
| A01089 | 3 | 1 | 2 |
| A01090 | 3 | 1 | 2 |
| A01091 | 3 | 1 | 2 |
| A01092 | 3 | 1 | 2 |
| A01093 | 3 | 1 | 2 |
| A01094 | 3 | 3 | 0 |
| A01095 | 3 | 1 | 2 |
| A01096 | 3 | 3 | 0 |
| A01097 | 3 | 3 | 0 |
| A01098 | 3 | 1 | 2 |
| A01101 | 3 | 1 | 2 |
| A01102 | 3 | 1 | 2 |
| A01104 | 3 | 1 | 2 |
| A01105 | 3 | 1 | 2 |
| A01106 | 3 | 1 | 2 |
| A01107 | 3 | 3 | 0 |
| A01108 | 3 | 2 | 1 |
| A01109 | 3 | 2 | 1 |
| A01110 | 3 | 1 | 2 |
| A01111 | 3 | 1 | 2 |
| A01113 | 3 | 1 | 2 |
| A01114 | 3 | 1 | 2 |
| A01115 | 3 | 1 | 2 |
| A01116 | 3 | 3 | 0 |
| A01117 | 3 | 1 | 2 |
| A01118 | 3 | 3 | 0 |
| A01119 | 3 | 3 | 0 |
| A01120 | 3 | 1 | 2 |
| A01121 | 3 | 2 | 1 |
| A01122 | 3 | 1 | 2 |
| A01123 | 3 | 3 | 0 |
| A01124 | 3 | 3 | 0 |
| A01125 | 3 | 3 | 0 |
| A01126 | 3 | 1 | 2 |
| A01127 | 3 | 2 | 1 |
| A01128 | 3 | 2 | 1 |
| A01129 | 3 | 3 | 0 |
| A01130 | 3 | 2 | 1 |
| A01131 | 3 | 2 | 1 |
| A01132 | 3 | 1 | 2 |
| A01133 | 3 | 1 | 2 |
| A01134 | 3 | 1 | 2 |
| A01135 | 3 | 1 | 2 |
| A01136 | 3 | 2 | 1 |
| A01137 | 3 | 1 | 2 |
| A01138 | 3 | 1 | 2 |
| A01139 | 3 | 1 | 2 |
| A01140 | 3 | 1 | 2 |
| A01141 | 3 | 1 | 2 |
| A01142 | 3 | 1 | 2 |
| A01143 | 3 | 3 | 0 |
| A01144 | 3 | 3 | 0 |
| A01145 | 3 | 1 | 2 |
| A01146 | 3 | 1 | 2 |
| A01147 | 3 | 3 | 0 |
| A01148 | 3 | 3 | 0 |
| A01149 | 3 | 3 | 0 |
| A01150 | 3 | 3 | 0 |
| A01151 | 3 | 1 | 2 |
| A01152 | 3 | 1 | 2 |
| A01153 | 3 | 3 | 0 |
| A01154 | 3 | 1 | 2 |
| A01155 | 3 | 1 | 2 |
| A01156 | 3 | 1 | 2 |
| A01157 | 3 | 1 | 2 |
| A01158 | 3 | 1 | 2 |
| A01159 | 3 | 1 | 2 |
| A01160 | 3 | 1 | 2 |
| A01161 | 3 | 1 | 2 |
| A01162 | 3 | 1 | 2 |
| A01163 | 3 | 1 | 2 |
| A01164 | 3 | 1 | 2 |
| A01165 | 3 | 3 | 0 |
| A01166 | 3 | 1 | 2 |
| A01167 | 3 | 3 | 0 |
| A01168 | 3 | 3 | 0 |
| A01169 | 3 | 1 | 2 |
| A01170 | 3 | 1 | 2 |
| A01171 | 3 | 1 | 2 |
| A01172 | 3 | 1 | 2 |
| A01174 | 3 | 3 | 0 |
| A01175 | 3 | 1 | 2 |
| A01176 | 3 | 1 | 2 |
| A01177 | 3 | 1 | 2 |
| A01178 | 3 | 1 | 2 |
| A01179 | 3 | 3 | 0 |
| A01180 | 3 | 1 | 2 |
| A01181 | 3 | 1 | 2 |
| A01182 | 3 | 1 | 2 |
| A01184 | 3 | 3 | 0 |
| A01185 | 3 | 1 | 2 |
| A01186 | 3 | 1 | 2 |
| A01187 | 3 | 3 | 0 |
| A01188 | 3 | 3 | 0 |
| A01189 | 3 | 3 | 0 |
| A01190 | 3 | 3 | 0 |
| A01191 | 3 | 3 | 0 |
| A01192 | 3 | 1 | 2 |
| A01193 | 3 | 3 | 0 |
| A01196 | 3 | 1 | 2 |
| A01197 | 3 | 1 | 2 |
| A01199 | 3 | 1 | 2 |
| A01200 | 3 | 3 | 0 |
| A01201 | 3 | 1 | 2 |
| A01202 | 3 | 1 | 2 |
| A01205 | 3 | 1 | 2 |
| A01207 | 3 | 2 | 1 |
| A01208 | 3 | 1 | 2 |
| A01209 | 3 | 1 | 2 |
| A01210 | 3 | 1 | 2 |
| A01211 | 3 | 1 | 2 |
| A01212 | 3 | 1 | 2 |
| A01214 | 3 | 3 | 0 |
| A01215 | 3 | 1 | 2 |
| A01216 | 3 | 2 | 1 |
| A01217 | 3 | 1 | 2 |
| A01218 | 3 | 2 | 1 |
| A01219 | 3 | 1 | 2 |
| A01220 | 3 | 1 | 2 |
| A01221 | 3 | 2 | 1 |
| A01222 | 3 | 1 | 2 |
| A01223 | 3 | 1 | 2 |
| A01224 | 3 | 1 | 2 |
| A01225 | 3 | 1 | 2 |
| A01226 | 3 | 1 | 2 |
| A01227 | 3 | 2 | 1 |
| A01228 | 3 | 3 | 0 |
| A01229 | 3 | 2 | 1 |
| A01230 | 3 | 1 | 2 |
| A01231 | 3 | 3 | 0 |
| A01232 | 3 | 1 | 2 |
| A01233 | 3 | 1 | 2 |
| A01234 | 3 | 1 | 2 |
| A01235 | 3 | 1 | 2 |
| A01236 | 3 | 1 | 2 |
| A01237 | 3 | 1 | 2 |
| A01238 | 3 | 3 | 0 |
| A01239 | 3 | 3 | 0 |
| A01240 | 3 | 3 | 0 |
| A01241 | 3 | 2 | 1 |
| A01242 | 3 | 1 | 2 |
| A01243 | 3 | 1 | 2 |
| A01244 | 3 | 3 | 0 |
| A01245 | 3 | 1 | 2 |
| A01246 | 3 | 1 | 2 |
| A01247 | 3 | 2 | 1 |
| A01248 | 3 | 1 | 2 |
| A01249 | 3 | 1 | 2 |
| A01250 | 3 | 1 | 2 |
| A01251 | 3 | 1 | 2 |
| A01252 | 3 | 2 | 1 |
| A01253 | 3 | 1 | 2 |
| A01254 | 3 | 2 | 1 |
| A01255 | 3 | 1 | 2 |
| A01256 | 3 | 1 | 2 |
| A01258 | 3 | 1 | 2 |
| A01259 | 3 | 2 | 1 |
| A01260 | 3 | 1 | 2 |
| A01261 | 3 | 1 | 2 |
| A01262 | 3 | 1 | 2 |
| A01263 | 3 | 1 | 2 |
| A01264 | 3 | 1 | 2 |
| A01265 | 3 | 1 | 2 |
| A01266 | 3 | 2 | 1 |
| A01267 | 3 | 1 | 2 |
| A01268 | 3 | 1 | 2 |
| A01269 | 3 | 1 | 2 |
| A01270 | 3 | 3 | 0 |
| A01271 | 3 | 1 | 2 |
| A01273 | 3 | 3 | 0 |
| A01274 | 3 | 1 | 2 |
| A01275 | 3 | 2 | 1 |
| A01276 | 3 | 2 | 1 |
| A01278 | 3 | 1 | 2 |
| A01280 | 3 | 1 | 2 |
| A01282 | 3 | 3 | 0 |
| A01283 | 3 | 3 | 0 |
| A01284 | 3 | 1 | 2 |
| A01286 | 3 | 1 | 2 |
| A01287 | 3 | 3 | 0 |
| A01288 | 3 | 1 | 2 |
| A01289 | 3 | 1 | 2 |
| A01290 | 3 | 2 | 1 |
| A01293 | 3 | 1 | 2 |
| A01294 | 3 | 1 | 2 |
| A01295 | 3 | 2 | 1 |
| A01296 | 3 | 1 | 2 |
| A01297 | 3 | 3 | 0 |
| A01298 | 3 | 1 | 2 |
| A01299 | 3 | 1 | 2 |
| A01302 | 3 | 1 | 2 |
| A01303 | 3 | 2 | 1 |
| A01304 | 3 | 3 | 0 |
| A01305 | 3 | 1 | 2 |
| A01306 | 3 | 3 | 0 |
| A01307 | 3 | 3 | 0 |
| A01308 | 3 | 2 | 1 |
| A01309 | 3 | 2 | 1 |
| A01310 | 3 | 1 | 2 |
| A01311 | 3 | 1 | 2 |
| A01312 | 3 | 2 | 1 |
| A01313 | 3 | 2 | 1 |
| A01316 | 3 | 1 | 2 |
| A01317 | 3 | 3 | 0 |
| A01318 | 3 | 1 | 2 |
| A01319 | 3 | 1 | 2 |
| A01320 | 3 | 1 | 2 |
| A01321 | 3 | 1 | 2 |
| A01322 | 3 | 1 | 2 |
| A01323 | 3 | 1 | 2 |
| A01324 | 3 | 1 | 2 |
| A01325 | 3 | 3 | 0 |
| A01326 | 3 | 3 | 0 |
| A01327 | 3 | 1 | 2 |
| A01328 | 3 | 2 | 1 |
| A01329 | 3 | 1 | 2 |
| A01330 | 3 | 1 | 2 |
| A01331 | 3 | 1 | 2 |
| A01332 | 3 | 3 | 0 |
| A01333 | 3 | 1 | 2 |
| A01334 | 3 | 1 | 2 |
| A01335 | 3 | 1 | 2 |
| A01336 | 3 | 1 | 2 |
| A01337 | 3 | 3 | 0 |
| A01340 | 3 | 3 | 0 |
| A01341 | 3 | 1 | 2 |
| A01342 | 3 | 3 | 0 |
| A01343 | 3 | 1 | 2 |
| A01344 | 3 | 3 | 0 |
| A01345 | 3 | 2 | 1 |
| A01346 | 3 | 3 | 0 |
| A01347 | 3 | 1 | 2 |
| A01348 | 3 | 1 | 2 |
| A01349 | 3 | 3 | 0 |
| A01350 | 3 | 1 | 2 |
| A01351 | 3 | 1 | 2 |
| A01352 | 3 | 1 | 2 |
| A01353 | 3 | 1 | 2 |
| A01354 | 3 | 1 | 2 |
| A01355 | 3 | 1 | 2 |
| A01356 | 3 | 3 | 0 |
| A01357 | 3 | 1 | 2 |
| A01358 | 3 | 1 | 2 |
| A01361 | 3 | 2 | 1 |
| A01362 | 3 | 1 | 2 |
| A01363 | 3 | 1 | 2 |
| A01364 | 3 | 3 | 0 |
| A01365 | 3 | 1 | 2 |
| A01366 | 3 | 1 | 2 |
| A01367 | 3 | 1 | 2 |
| A01368 | 3 | 2 | 1 |
| A01369 | 3 | 1 | 2 |
| A01373 | 3 | 1 | 2 |
| A01375 | 3 | 1 | 2 |
| A01376 | 3 | 3 | 0 |
| A01377 | 3 | 1 | 2 |
| A01378 | 3 | 3 | 0 |
| A01379 | 3 | 1 | 2 |
| A01380 | 3 | 3 | 0 |
| A01381 | 3 | 3 | 0 |
| A01382 | 3 | 1 | 2 |
| A01383 | 3 | 3 | 0 |
| A01385 | 3 | 3 | 0 |
| A01386 | 3 | 3 | 0 |
| A01387 | 3 | 2 | 1 |
| A01388 | 3 | 1 | 2 |
| A01389 | 3 | 1 | 2 |
| A01390 | 3 | 1 | 2 |
| A01392 | 3 | 3 | 0 |
| A01393 | 3 | 3 | 0 |
| A01394 | 3 | 1 | 2 |
| A01395 | 3 | 1 | 2 |
| A01396 | 3 | 3 | 0 |
| A01397 | 3 | 1 | 2 |
| A01398 | 3 | 3 | 0 |
| A01400 | 3 | 1 | 2 |
| A01401 | 3 | 1 | 2 |
| A01402 | 3 | 1 | 2 |
| A01403 | 3 | 1 | 2 |
| A01404 | 3 | 1 | 2 |
| A01408 | 3 | 1 | 2 |
| A01409 | 3 | 3 | 0 |
| A01410 | 3 | 2 | 1 |
| A01411 | 3 | 1 | 2 |
| A01412 | 3 | 1 | 2 |
| A01413 | 3 | 1 | 2 |
| A01414 | 3 | 3 | 0 |
| A01416 | 3 | 3 | 0 |
| A01417 | 3 | 1 | 2 |
| A01418 | 3 | 1 | 2 |
| A01419 | 3 | 1 | 2 |
| A01420 | 3 | 3 | 0 |
| A01421 | 3 | 1 | 2 |
| A01422 | 3 | 1 | 2 |
| A01423 | 3 | 1 | 2 |
| A01424 | 3 | 1 | 2 |
| A01425 | 3 | 3 | 0 |
| A01426 | 3 | 1 | 2 |
| A01427 | 3 | 3 | 0 |
| A01428 | 3 | 1 | 2 |
| A01429 | 3 | 3 | 0 |
| A01430 | 3 | 1 | 2 |
| A01431 | 3 | 3 | 0 |
| A01432 | 3 | 1 | 2 |
| A01433 | 3 | 3 | 0 |
| A01434 | 3 | 1 | 2 |
| A01435 | 3 | 1 | 2 |
| A01436 | 3 | 1 | 2 |
| A01437 | 3 | 3 | 0 |
| A01438 | 3 | 1 | 2 |
| A01440 | 3 | 1 | 2 |
| A01441 | 3 | 2 | 1 |
| A01442 | 3 | 2 | 1 |
| A01443 | 3 | 3 | 0 |
| A01444 | 3 | 1 | 2 |
| A01445 | 3 | 1 | 2 |
| A01446 | 3 | 1 | 2 |
| A01447 | 3 | 1 | 2 |
| A01448 | 3 | 1 | 2 |
| A01449 | 3 | 1 | 2 |
| A01450 | 3 | 1 | 2 |
| A01451 | 3 | 1 | 2 |
| A01452 | 3 | 1 | 2 |
| A01453 | 3 | 2 | 1 |
| A01454 | 3 | 2 | 1 |
| A01455 | 3 | 1 | 2 |
| A01456 | 3 | 1 | 2 |
| A01457 | 3 | 1 | 2 |
| A01458 | 3 | 1 | 2 |
| A01459 | 3 | 1 | 2 |
| A01460 | 3 | 1 | 2 |
| A01461 | 3 | 2 | 1 |
| A01462 | 3 | 3 | 0 |
| A01463 | 3 | 1 | 2 |
| A01464 | 3 | 1 | 2 |
| A01465 | 3 | 1 | 2 |
| A01466 | 3 | 2 | 1 |
| A01467 | 3 | 3 | 0 |
| A01468 | 3 | 3 | 0 |
| A01469 | 3 | 1 | 2 |
| A01470 | 3 | 3 | 0 |
| A01471 | 3 | 3 | 0 |
| A01472 | 3 | 1 | 2 |
| A01473 | 3 | 2 | 1 |
| A01474 | 3 | 2 | 1 |
| A01475 | 3 | 1 | 2 |
| A01476 | 3 | 3 | 0 |
| A01478 | 3 | 2 | 1 |
| A01480 | 3 | 1 | 2 |
| A01481 | 3 | 3 | 0 |
| A01482 | 3 | 1 | 2 |
| A01483 | 3 | 1 | 2 |
| A01484 | 3 | 3 | 0 |
| A01485 | 3 | 2 | 1 |
| A01486 | 3 | 3 | 0 |
| A01487 | 3 | 1 | 2 |
| A01488 | 3 | 2 | 1 |
| A01489 | 3 | 1 | 2 |
| A01491 | 3 | 1 | 2 |
| A01492 | 3 | 2 | 1 |
| A01493 | 3 | 2 | 1 |
| A01494 | 3 | 2 | 1 |
| A01496 | 3 | 1 | 2 |
| A01497 | 3 | 3 | 0 |
| A01498 | 3 | 1 | 2 |
| A01499 | 3 | 1 | 2 |
| A01500 | 3 | 1 | 2 |
| A01501 | 3 | 1 | 2 |
| A01502 | 3 | 1 | 2 |
| A01503 | 3 | 1 | 2 |
| A01504 | 3 | 3 | 0 |
| A01505 | 3 | 3 | 0 |
| A01506 | 3 | 1 | 2 |
| A01507 | 3 | 3 | 0 |
| A01508 | 3 | 2 | 1 |
| A01509 | 3 | 2 | 1 |
| A01510 | 3 | 3 | 0 |
| A01511 | 3 | 2 | 1 |
| A01512 | 3 | 3 | 0 |
| A01513 | 3 | 3 | 0 |
| A01514 | 3 | 3 | 0 |
| A01515 | 3 | 2 | 1 |
| A01516 | 3 | 1 | 2 |
| A01517 | 3 | 3 | 0 |
| A01518 | 3 | 3 | 0 |
| A01519 | 3 | 3 | 0 |
| A01520 | 3 | 3 | 0 |
| A01521 | 3 | 3 | 0 |
| A01522 | 3 | 1 | 2 |
| A01523 | 3 | 1 | 2 |
| A01524 | 3 | 1 | 2 |
| A01525 | 3 | 3 | 0 |
| A01526 | 3 | 2 | 1 |
| A01527 | 3 | 2 | 1 |
| A01528 | 3 | 3 | 0 |
| A01529 | 3 | 3 | 0 |
| A01530 | 3 | 1 | 2 |
| A01531 | 3 | 1 | 2 |
| A01532 | 3 | 1 | 2 |
| A01535 | 3 | 3 | 0 |
| A01536 | 3 | 1 | 2 |
| A01537 | 3 | 1 | 2 |
| A01538 | 3 | 1 | 2 |
| A01539 | 3 | 1 | 2 |
| A01545 | 3 | 1 | 2 |
| A01546 | 3 | 1 | 2 |
| A01547 | 3 | 1 | 2 |
| A01548 | 3 | 3 | 0 |
| A01549 | 3 | 1 | 2 |
| A01550 | 3 | 2 | 1 |
| A01551 | 3 | 3 | 0 |
| A01552 | 3 | 1 | 2 |
| A01553 | 3 | 1 | 2 |
| A01554 | 3 | 2 | 1 |
| A01555 | 3 | 1 | 2 |
| A01556 | 3 | 1 | 2 |
| A01558 | 3 | 3 | 0 |
| A01559 | 3 | 1 | 2 |
| A01562 | 3 | 1 | 2 |
| A01563 | 3 | 1 | 2 |
| A01564 | 3 | 1 | 2 |
| A01565 | 3 | 1 | 2 |
| A01566 | 3 | 1 | 2 |
| A01567 | 3 | 1 | 2 |
| A01568 | 3 | 3 | 0 |
| A01569 | 3 | 1 | 2 |
| A01570 | 3 | 3 | 0 |
| A01571 | 3 | 1 | 2 |
| A01572 | 3 | 1 | 2 |
| A01574 | 3 | 1 | 2 |
| A01575 | 3 | 3 | 0 |
| A01576 | 3 | 3 | 0 |
| A01580 | 3 | 1 | 2 |
| A01581 | 3 | 3 | 0 |
| A01586 | 3 | 3 | 0 |
| A01587 | 3 | 1 | 2 |
| A01588 | 3 | 1 | 2 |
| A01589 | 3 | 1 | 2 |
| A01591 | 3 | 1 | 2 |
| A01593 | 3 | 1 | 2 |
| A01594 | 3 | 3 | 0 |
| A01595 | 3 | 1 | 2 |
| A01596 | 3 | 3 | 0 |
| A01597 | 3 | 1 | 2 |
| A01598 | 3 | 3 | 0 |
| A01599 | 3 | 3 | 0 |
| A01602 | 3 | 1 | 2 |
| A01603 | 3 | 1 | 2 |
| A01604 | 3 | 3 | 0 |
| A01605 | 3 | 1 | 2 |
| A01606 | 3 | 1 | 2 |
| A01607 | 3 | 1 | 2 |
| A01608 | 3 | 1 | 2 |
| A01609 | 3 | 1 | 2 |
| A01610 | 3 | 3 | 0 |
| A01611 | 3 | 3 | 0 |
| A01612 | 3 | 3 | 0 |
| A01613 | 3 | 3 | 0 |
| A01614 | 3 | 1 | 2 |
| A01616 | 3 | 1 | 2 |
| A01617 | 3 | 1 | 2 |
| A01618 | 3 | 1 | 2 |
| A01619 | 3 | 1 | 2 |
| A01620 | 3 | 3 | 0 |
| A01621 | 3 | 1 | 2 |
| A01623 | 3 | 1 | 2 |
| A01624 | 3 | 1 | 2 |
| A01625 | 3 | 1 | 2 |
| A01626 | 3 | 1 | 2 |
| A01629 | 3 | 1 | 2 |
| A01632 | 3 | 3 | 0 |
| A01633 | 3 | 1 | 2 |
| A01634 | 3 | 1 | 2 |
| A01635 | 3 | 3 | 0 |
| A01636 | 3 | 1 | 2 |
| A01637 | 3 | 1 | 2 |
| A01638 | 3 | 1 | 2 |
| A01639 | 3 | 3 | 0 |
| A01640 | 3 | 3 | 0 |
| A01641 | 3 | 1 | 2 |
| A01642 | 3 | 1 | 2 |
| A01644 | 3 | 1 | 2 |
| A01646 | 3 | 2 | 1 |
| A01647 | 3 | 3 | 0 |
| A01648 | 3 | 1 | 2 |
| A01649 | 3 | 1 | 2 |
| A01650 | 3 | 1 | 2 |
| A01651 | 3 | 1 | 2 |
| A01652 | 3 | 1 | 2 |
| A01653 | 3 | 3 | 0 |
| A01654 | 3 | 1 | 2 |
| A01655 | 3 | 1 | 2 |
| A01656 | 3 | 1 | 2 |
| A01657 | 3 | 1 | 2 |
| A01658 | 3 | 1 | 2 |
| A01659 | 3 | 1 | 2 |
| A01660 | 3 | 2 | 1 |
| A01661 | 3 | 3 | 0 |
| A01662 | 3 | 3 | 0 |
| A01663 | 3 | 3 | 0 |
| A01664 | 3 | 1 | 2 |
| A01665 | 3 | 1 | 2 |
| A01666 | 3 | 1 | 2 |
| A01667 | 3 | 1 | 2 |
| A01668 | 3 | 1 | 2 |
| A01669 | 3 | 1 | 2 |
| A01670 | 3 | 1 | 2 |
| A01671 | 3 | 1 | 2 |
| A01672 | 3 | 2 | 1 |
| A01673 | 3 | 1 | 2 |
| A01674 | 3 | 1 | 2 |
| A01675 | 3 | 1 | 2 |
| A01677 | 3 | 1 | 2 |
| A01678 | 3 | 1 | 2 |
| A01679 | 3 | 2 | 1 |
| A01680 | 3 | 2 | 1 |
| A01681 | 3 | 1 | 2 |
| A01682 | 3 | 1 | 2 |
| A01683 | 3 | 2 | 1 |
| A01684 | 3 | 1 | 2 |
| A01685 | 3 | 1 | 2 |
| A01687 | 3 | 2 | 1 |
| A01688 | 3 | 1 | 2 |
| A01689 | 3 | 1 | 2 |
| A01690 | 3 | 1 | 2 |
| A01691 | 3 | 1 | 2 |
| A01692 | 3 | 1 | 2 |
| A01693 | 3 | 1 | 2 |
| A01694 | 3 | 1 | 2 |
| A01695 | 3 | 1 | 2 |
| A01696 | 3 | 1 | 2 |
| A01697 | 3 | 2 | 1 |
| A01698 | 3 | 2 | 1 |
| A01699 | 3 | 1 | 2 |
| A01700 | 3 | 2 | 1 |
| A01701 | 3 | 1 | 2 |
| A01702 | 3 | 1 | 2 |
| A01703 | 3 | 1 | 2 |
| A01704 | 3 | 1 | 2 |
| A01705 | 3 | 1 | 2 |
| A01706 | 3 | 1 | 2 |
| A01707 | 3 | 1 | 2 |
| A01709 | 3 | 1 | 2 |
| A01710 | 3 | 1 | 2 |
| A01711 | 3 | 1 | 2 |
| A01713 | 3 | 1 | 2 |
| A01715 | 3 | 1 | 2 |
| A01716 | 3 | 1 | 2 |
| A01722 | 3 | 1 | 2 |
| A01723 | 3 | 1 | 2 |
| A01724 | 3 | 1 | 2 |
| A01725 | 3 | 1 | 2 |
| A01726 | 3 | 1 | 2 |
| A01727 | 3 | 1 | 2 |
| A01729 | 3 | 1 | 2 |
| A01730 | 3 | 1 | 2 |
| A01732 | 3 | 1 | 2 |
| A01733 | 3 | 1 | 2 |
| A01734 | 3 | 1 | 2 |
| A01735 | 3 | 1 | 2 |
| A01736 | 3 | 1 | 2 |
| A01737 | 3 | 2 | 1 |
| A01740 | 3 | 1 | 2 |
| A01741 | 3 | 1 | 2 |
| A01742 | 3 | 1 | 2 |
| A01743 | 3 | 1 | 2 |
| A01744 | 3 | 1 | 2 |
| A01745 | 3 | 1 | 2 |
| A01746 | 3 | 1 | 2 |
| A01747 | 3 | 1 | 2 |
| A01748 | 3 | 1 | 2 |
| A01749 | 3 | 1 | 2 |
| A01750 | 3 | 1 | 2 |
| A01751 | 3 | 1 | 2 |
| A01752 | 3 | 1 | 2 |
| A01753 | 3 | 1 | 2 |
| A01754 | 3 | 1 | 2 |
| A01763 | 3 | 1 | 2 |
| A01765 | 3 | 1 | 2 |
| A01774 | 3 | 2 | 1 |
| A01776 | 3 | 1 | 2 |
| A01777 | 3 | 1 | 2 |
| A01778 | 3 | 1 | 2 |
| A01779 | 3 | 1 | 2 |
| A01780 | 3 | 1 | 2 |
| A01781 | 3 | 1 | 2 |
| A01784 | 3 | 1 | 2 |
| A01785 | 3 | 1 | 2 |
| A01786 | 3 | 1 | 2 |
| A01787 | 3 | 1 | 2 |
| A01788 | 3 | 1 | 2 |
| A01789 | 3 | 1 | 2 |
| A01791 | 3 | 1 | 2 |
| A01792 | 3 | 1 | 2 |
| A01794 | 3 | 1 | 2 |
| A01797 | 3 | 1 | 2 |
| A01799 | 3 | 3 | 0 |
| A01800 | 3 | 1 | 2 |
| A01801 | 3 | 1 | 2 |
| A01802 | 3 | 1 | 2 |
| A01803 | 3 | 1 | 2 |
| A01805 | 3 | 1 | 2 |
| A01806 | 3 | 3 | 0 |
| A01807 | 3 | 1 | 2 |
| A01809 | 3 | 1 | 2 |
| A01810 | 3 | 1 | 2 |
| A01811 | 3 | 1 | 2 |
| A01812 | 3 | 1 | 2 |
| A01814 | 3 | 1 | 2 |
| A01815 | 3 | 1 | 2 |
| A01816 | 3 | 1 | 2 |
| A01817 | 3 | 1 | 2 |
| A01819 | 3 | 3 | 0 |
| A01822 | 3 | 1 | 2 |
| A01824 | 3 | 1 | 2 |
| A01826 | 3 | 1 | 2 |
| A01827 | 3 | 2 | 1 |
| A01828 | 3 | 1 | 2 |
| A01829 | 3 | 1 | 2 |
| A01830 | 3 | 1 | 2 |
| A01832 | 3 | 1 | 2 |
| A01849 | 3 | 1 | 2 |
| A01850 | 3 | 1 | 2 |
| A01851 | 3 | 1 | 2 |
| A01852 | 3 | 1 | 2 |
| A01854 | 3 | 3 | 0 |
| A01855 | 3 | 3 | 0 |
| A01856 | 3 | 1 | 2 |
| A01857 | 3 | 1 | 2 |
| A01858 | 3 | 3 | 0 |
| A01859 | 3 | 3 | 0 |
| A01860 | 3 | 3 | 0 |
| A01861 | 3 | 2 | 1 |
| A01862 | 3 | 1 | 2 |
| A01863 | 3 | 1 | 2 |
| A01864 | 3 | 3 | 0 |
| A01865 | 3 | 3 | 0 |
| A01866 | 3 | 1 | 2 |
| A01867 | 3 | 1 | 2 |
| A01868 | 3 | 3 | 0 |
| A01869 | 3 | 3 | 0 |
| A01870 | 3 | 1 | 2 |
| A01871 | 3 | 1 | 2 |
| A01873 | 3 | 1 | 2 |
| A01874 | 3 | 3 | 0 |
| A01875 | 3 | 3 | 0 |
| A01876 | 3 | 2 | 1 |
| A01877 | 3 | 3 | 0 |
| A01884 | 3 | 1 | 2 |
| A01885 | 3 | 1 | 2 |
| A01886 | 3 | 1 | 2 |
| A01887 | 3 | 2 | 1 |
| A01888 | 3 | 3 | 0 |
| A01889 | 3 | 1 | 2 |
| A01890 | 3 | 1 | 2 |
| A01892 | 3 | 1 | 2 |
| A01893 | 3 | 3 | 0 |
| A01894 | 3 | 3 | 0 |
| A01895 | 3 | 3 | 0 |
| A01896 | 3 | 1 | 2 |
| A01897 | 3 | 1 | 2 |
| A01898 | 3 | 1 | 2 |
| A01899 | 3 | 1 | 2 |
| A01900 | 3 | 1 | 2 |
| A01901 | 3 | 3 | 0 |
| A01902 | 3 | 3 | 0 |
| A01904 | 3 | 3 | 0 |
| A01905 | 3 | 3 | 0 |
| A01906 | 3 | 1 | 2 |
| A01907 | 3 | 1 | 2 |
| A01908 | 3 | 1 | 2 |
| A01909 | 3 | 1 | 2 |
| A01910 | 3 | 2 | 1 |
| A01911 | 3 | 1 | 2 |
| A01912 | 3 | 1 | 2 |
| A01913 | 3 | 1 | 2 |
| A01914 | 3 | 1 | 2 |
| A01915 | 3 | 1 | 2 |
| A01916 | 3 | 2 | 1 |
| A01917 | 3 | 1 | 2 |
| A01918 | 3 | 3 | 0 |
| A01919 | 3 | 3 | 0 |
| A01920 | 3 | 3 | 0 |
| A01921 | 3 | 1 | 2 |
| A01922 | 3 | 3 | 0 |
| A01923 | 3 | 1 | 2 |
| A01924 | 3 | 3 | 0 |
| A01925 | 3 | 3 | 0 |
| A01926 | 3 | 2 | 1 |
| A01927 | 3 | 1 | 2 |
| A01928 | 3 | 1 | 2 |
| A01929 | 3 | 3 | 0 |
| A01930 | 3 | 3 | 0 |
| A01932 | 3 | 2 | 1 |
| A01933 | 3 | 3 | 0 |
| A01934 | 3 | 3 | 0 |
| A01935 | 3 | 3 | 0 |
| A01936 | 3 | 3 | 0 |
| A01937 | 3 | 3 | 0 |
| A01938 | 3 | 3 | 0 |
| A01939 | 3 | 3 | 0 |
| A01940 | 3 | 3 | 0 |
| A01941 | 3 | 3 | 0 |
| A01943 | 3 | 1 | 2 |
| A01944 | 3 | 3 | 0 |
| A01945 | 3 | 1 | 2 |
| A01946 | 3 | 3 | 0 |
| A01947 | 3 | 3 | 0 |
| A01948 | 3 | 2 | 1 |
| A01949 | 3 | 3 | 0 |
| A01950 | 3 | 3 | 0 |
| A01951 | 3 | 3 | 0 |
| A01952 | 3 | 3 | 0 |
| A01953 | 3 | 3 | 0 |
| A01954 | 3 | 3 | 0 |
| A01955 | 3 | 3 | 0 |
| A01956 | 3 | 3 | 0 |
| A01957 | 3 | 1 | 2 |
| A01958 | 3 | 3 | 0 |
| A01959 | 3 | 1 | 2 |
| A01960 | 3 | 3 | 0 |
| A01962 | 3 | 1 | 2 |
| A01963 | 3 | 1 | 2 |
| A01964 | 3 | 1 | 2 |
| A01966 | 3 | 3 | 0 |
| A01967 | 3 | 1 | 2 |
| A01968 | 3 | 3 | 0 |
| A01969 | 3 | 1 | 2 |
| A01970 | 3 | 1 | 2 |
| A01971 | 3 | 1 | 2 |
| A01972 | 3 | 1 | 2 |
| A01973 | 3 | 1 | 2 |
| A01974 | 3 | 1 | 2 |
| A01975 | 3 | 3 | 0 |
| A01976 | 3 | 3 | 0 |
| A01977 | 3 | 2 | 1 |
| A01978 | 3 | 3 | 0 |
| A01979 | 3 | 3 | 0 |
| A01980 | 3 | 3 | 0 |
| A01981 | 3 | 3 | 0 |
| A01982 | 3 | 1 | 2 |
| A01984 | 3 | 2 | 1 |
| A01985 | 3 | 3 | 0 |
| A01986 | 3 | 3 | 0 |
| A01987 | 3 | 1 | 2 |
| A01988 | 3 | 1 | 2 |
| A01989 | 3 | 1 | 2 |
| A01991 | 3 | 2 | 1 |
| A01992 | 3 | 3 | 0 |
| A01993 | 3 | 2 | 1 |
| A01994 | 3 | 3 | 0 |
| A01995 | 3 | 3 | 0 |
| A01997 | 3 | 3 | 0 |
| A01998 | 3 | 2 | 1 |
| A02000 | 3 | 1 | 2 |
| A02001 | 3 | 3 | 0 |
| A02002 | 3 | 2 | 1 |
| A02003 | 3 | 3 | 0 |
| A02004 | 3 | 2 | 1 |
| A02005 | 3 | 2 | 1 |
| A02006 | 3 | 3 | 0 |
| A02007 | 3 | 2 | 1 |
| A02008 | 3 | 3 | 0 |
| A02009 | 3 | 2 | 1 |
| A02010 | 3 | 3 | 0 |
| A02011 | 3 | 3 | 0 |
| A02012 | 3 | 3 | 0 |
| A02013 | 3 | 2 | 1 |
| A02014 | 3 | 3 | 0 |
| A02015 | 3 | 3 | 0 |
| A02016 | 3 | 3 | 0 |
| A02017 | 3 | 3 | 0 |
| A02018 | 3 | 3 | 0 |
| A02019 | 3 | 2 | 1 |
| A02020 | 3 | 2 | 1 |
| A02021 | 3 | 1 | 2 |
| A02022 | 3 | 3 | 0 |
| A02023 | 3 | 2 | 1 |
| A02024 | 3 | 3 | 0 |
| A02025 | 3 | 2 | 1 |
| A02026 | 3 | 2 | 1 |
| A02027 | 3 | 2 | 1 |
| A02028 | 3 | 3 | 0 |
| A02029 | 3 | 2 | 1 |
| A02030 | 3 | 1 | 2 |
| A02031 | 3 | 1 | 2 |
| A02032 | 3 | 3 | 0 |
| A02034 | 3 | 3 | 0 |
| A02035 | 3 | 3 | 0 |
| A02036 | 3 | 3 | 0 |
| A02037 | 3 | 2 | 1 |
| A02038 | 3 | 3 | 0 |
| A02039 | 3 | 2 | 1 |
| A02040 | 3 | 3 | 0 |
| A02041 | 3 | 2 | 1 |
| A02042 | 3 | 1 | 2 |
| A02043 | 3 | 3 | 0 |
| A02044 | 3 | 2 | 1 |
| A02045 | 3 | 3 | 0 |
| A02046 | 3 | 3 | 0 |
| A02047 | 3 | 1 | 2 |
| A02048 | 3 | 3 | 0 |
| A02049 | 3 | 3 | 0 |
| A02050 | 3 | 3 | 0 |
| A02051 | 3 | 1 | 2 |
| A02052 | 3 | 3 | 0 |
| A02053 | 3 | 3 | 0 |
| A02054 | 3 | 3 | 0 |
| A02055 | 3 | 1 | 2 |
| A02056 | 3 | 3 | 0 |
| A02057 | 3 | 3 | 0 |
| A02058 | 3 | 2 | 1 |
| A02059 | 3 | 3 | 0 |
| A02060 | 3 | 3 | 0 |
| A02061 | 3 | 3 | 0 |
| A02062 | 3 | 1 | 2 |
| A02063 | 3 | 3 | 0 |
| A02064 | 3 | 3 | 0 |
| A02065 | 3 | 3 | 0 |
| A02066 | 3 | 3 | 0 |
| A02067 | 3 | 1 | 2 |
| A02068 | 3 | 3 | 0 |
| A02069 | 3 | 1 | 2 |
| A02070 | 3 | 3 | 0 |
| A02071 | 3 | 2 | 1 |
| A02072 | 3 | 1 | 2 |
| A02073 | 3 | 3 | 0 |
| A02074 | 3 | 1 | 2 |
| A02075 | 3 | 1 | 2 |
| A02076 | 3 | 3 | 0 |
| A02077 | 3 | 3 | 0 |
| A02078 | 3 | 1 | 2 |
| A02079 | 3 | 3 | 0 |
| A02080 | 3 | 3 | 0 |
| A02081 | 3 | 1 | 2 |
| A02082 | 3 | 3 | 0 |
| A02083 | 3 | 1 | 2 |
| A02084 | 3 | 1 | 2 |
| A02085 | 3 | 1 | 2 |
| A02086 | 3 | 1 | 2 |
| A02088 | 3 | 1 | 2 |
| A02090 | 3 | 1 | 2 |
| A02091 | 3 | 1 | 2 |
| A02092 | 3 | 1 | 2 |
| A02093 | 3 | 1 | 2 |
| A02094 | 3 | 2 | 1 |
| A02095 | 3 | 1 | 2 |
| A02096 | 3 | 3 | 0 |
| A02097 | 3 | 3 | 0 |
| A02098 | 3 | 3 | 0 |
| A02099 | 3 | 3 | 0 |
| A02100 | 3 | 2 | 1 |
| A02101 | 3 | 3 | 0 |
| A02102 | 3 | 1 | 2 |
| A02103 | 3 | 3 | 0 |
| A02104 | 3 | 3 | 0 |
| A02105 | 3 | 1 | 2 |
| A02107 | 3 | 1 | 2 |
| A02108 | 3 | 1 | 2 |
| A02109 | 3 | 2 | 1 |
| A02110 | 3 | 3 | 0 |
| A02111 | 3 | 2 | 1 |
| A02112 | 3 | 1 | 2 |
| A02113 | 3 | 3 | 0 |
| A02114 | 3 | 2 | 1 |
| A02115 | 3 | 2 | 1 |
| A02116 | 3 | 1 | 2 |
| A02117 | 3 | 2 | 1 |
| A02118 | 3 | 3 | 0 |
| A02119 | 3 | 3 | 0 |
| A02120 | 3 | 3 | 0 |
| A02121 | 3 | 3 | 0 |
| A02122 | 3 | 3 | 0 |
| A02123 | 3 | 3 | 0 |
| A02124 | 3 | 1 | 2 |
| A02125 | 3 | 2 | 1 |
| A02126 | 3 | 2 | 1 |
| A02127 | 3 | 1 | 2 |
| A02128 | 3 | 2 | 1 |
| A02129 | 3 | 3 | 0 |
| A02130 | 3 | 3 | 0 |
| A02131 | 3 | 3 | 0 |
| A02132 | 3 | 1 | 2 |
| A02133 | 3 | 1 | 2 |
| A02134 | 3 | 1 | 2 |
| A02135 | 3 | 2 | 1 |
| A02136 | 3 | 2 | 1 |
| A02137 | 3 | 1 | 2 |
| A02139 | 3 | 2 | 1 |
| A02142 | 3 | 1 | 2 |
| A02143 | 3 | 2 | 1 |
| A02144 | 3 | 2 | 1 |
| A02145 | 3 | 3 | 0 |
| A02146 | 3 | 1 | 2 |
| A02147 | 3 | 2 | 1 |
| A02148 | 3 | 2 | 1 |
| A02149 | 3 | 3 | 0 |
| A02150 | 3 | 2 | 1 |
| A02151 | 3 | 1 | 2 |
| A02152 | 3 | 2 | 1 |
| A02153 | 3 | 2 | 1 |
| A02154 | 3 | 2 | 1 |
| A02162 | 3 | 3 | 0 |
| A02163 | 3 | 3 | 0 |
| A02164 | 3 | 3 | 0 |
| A02165 | 3 | 3 | 0 |
| A02166 | 3 | 3 | 0 |
| A02167 | 3 | 3 | 0 |
| A02168 | 3 | 3 | 0 |
| A02169 | 3 | 1 | 2 |
| A02170 | 3 | 2 | 1 |
| A02171 | 3 | 1 | 2 |
| A02172 | 3 | 1 | 2 |
| A02173 | 3 | 2 | 1 |
| A02174 | 3 | 3 | 0 |
| A02175 | 3 | 1 | 2 |
| A02176 | 3 | 1 | 2 |
| A02177 | 3 | 1 | 2 |
| A02178 | 3 | 3 | 0 |
| A02179 | 3 | 1 | 2 |
| A02180 | 3 | 2 | 1 |
| A02181 | 3 | 2 | 1 |
| A02182 | 3 | 1 | 2 |
| A02183 | 3 | 2 | 1 |
| A02184 | 3 | 3 | 0 |
| A02185 | 3 | 3 | 0 |
| A02186 | 3 | 3 | 0 |
| A02187 | 3 | 3 | 0 |
| A02191 | 3 | 3 | 0 |
| A02192 | 3 | 3 | 0 |
| A02193 | 3 | 2 | 1 |
| A02196 | 3 | 3 | 0 |
| A02197 | 3 | 2 | 1 |
| A02198 | 3 | 1 | 2 |
| A02199 | 3 | 1 | 2 |
| A02202 | 3 | 3 | 0 |
| A02203 | 3 | 1 | 2 |
| A02204 | 3 | 3 | 0 |
| A02205 | 3 | 1 | 2 |
| A02206 | 3 | 1 | 2 |
| A02207 | 3 | 2 | 1 |
| A02208 | 3 | 1 | 2 |
| A02209 | 3 | 3 | 0 |
| A02210 | 3 | 2 | 1 |
| A02211 | 3 | 3 | 0 |
| A02212 | 3 | 3 | 0 |
| A02213 | 3 | 3 | 0 |
| A02214 | 3 | 2 | 1 |
| A02216 | 3 | 2 | 1 |
| A02217 | 3 | 1 | 2 |
| A02218 | 3 | 1 | 2 |
| A02219 | 3 | 3 | 0 |
| A02221 | 3 | 3 | 0 |
| A02222 | 3 | 1 | 2 |
| A02223 | 3 | 1 | 2 |
| A02224 | 3 | 2 | 1 |
| A02225 | 3 | 1 | 2 |
| A02226 | 3 | 2 | 1 |
| A02227 | 3 | 2 | 1 |
| A02228 | 3 | 1 | 2 |
| A02229 | 3 | 1 | 2 |
| A02230 | 3 | 1 | 2 |
| A02231 | 3 | 2 | 1 |
| A02232 | 3 | 2 | 1 |
| A02233 | 3 | 1 | 2 |
| A02235 | 3 | 1 | 2 |
| A02236 | 3 | 2 | 1 |
| A02237 | 3 | 2 | 1 |
| A02238 | 3 | 1 | 2 |
| A02239 | 3 | 2 | 1 |
| A02240 | 3 | 1 | 2 |
| A02242 | 3 | 2 | 1 |
| A02243 | 3 | 2 | 1 |
| A02244 | 3 | 1 | 2 |
| A02245 | 3 | 1 | 2 |
| A02246 | 3 | 2 | 1 |
| A02247 | 3 | 2 | 1 |
| A02248 | 3 | 1 | 2 |
| A02249 | 3 | 2 | 1 |
| A02250 | 3 | 1 | 2 |
| A02251 | 3 | 1 | 2 |
| A02252 | 3 | 1 | 2 |
| A02253 | 3 | 1 | 2 |
| A02254 | 3 | 1 | 2 |
| A02255 | 3 | 1 | 2 |
| A02256 | 3 | 1 | 2 |
| A02257 | 3 | 1 | 2 |
| A02258 | 3 | 1 | 2 |
| A02259 | 3 | 1 | 2 |
| A02260 | 3 | 1 | 2 |
| A02261 | 3 | 2 | 1 |
| A02262 | 3 | 2 | 1 |
| A02263 | 3 | 1 | 2 |
| A02264 | 3 | 1 | 2 |
| A02265 | 3 | 2 | 1 |
| A02266 | 3 | 1 | 2 |
| A02267 | 3 | 1 | 2 |
| A02268 | 3 | 1 | 2 |
| A02269 | 3 | 3 | 0 |
| A02270 | 3 | 3 | 0 |
| A02271 | 3 | 3 | 0 |
| A02273 | 3 | 1 | 2 |
| A02274 | 3 | 1 | 2 |
| A02275 | 3 | 3 | 0 |
| A02276 | 3 | 1 | 2 |
| A02277 | 3 | 1 | 2 |
| A02278 | 3 | 2 | 1 |
| A02279 | 3 | 1 | 2 |
| A02280 | 3 | 2 | 1 |
| A02281 | 3 | 1 | 2 |
| A02282 | 3 | 2 | 1 |
| A02283 | 3 | 1 | 2 |
| A02284 | 3 | 1 | 2 |
| A02285 | 3 | 2 | 1 |
| A02286 | 3 | 2 | 1 |
| A02287 | 3 | 1 | 2 |
| A02290 | 3 | 2 | 1 |
| A02334 | 3 | 2 | 1 |
| A02335 | 3 | 2 | 1 |
| A02336 | 3 | 3 | 0 |
| A02337 | 3 | 3 | 0 |
| A02338 | 3 | 2 | 1 |
| A02339 | 3 | 1 | 2 |
| A02341 | 3 | 3 | 0 |
| A02343 | 3 | 1 | 2 |
| A02344 | 3 | 2 | 1 |
| A02345 | 3 | 3 | 0 |
| A02346 | 3 | 1 | 2 |
| A02347 | 3 | 3 | 0 |
| A02348 | 3 | 2 | 1 |
| A02349 | 3 | 1 | 2 |
| A02350 | 3 | 1 | 2 |
| A02351 | 3 | 2 | 1 |
| A02352 | 3 | 2 | 1 |
| A02353 | 3 | 3 | 0 |
| A02354 | 3 | 2 | 1 |
| A02355 | 3 | 2 | 1 |
| A02356 | 3 | 2 | 1 |
| A02359 | 3 | 1 | 2 |
| A02360 | 3 | 1 | 2 |
| A02361 | 3 | 2 | 1 |
| A02362 | 3 | 1 | 2 |
| A02363 | 3 | 2 | 1 |
| A02364 | 3 | 1 | 2 |
| A02365 | 3 | 1 | 2 |
| A02367 | 3 | 1 | 2 |
| A02368 | 3 | 2 | 1 |
| A02369 | 3 | 3 | 0 |
| A02370 | 3 | 1 | 2 |
| A02371 | 3 | 3 | 0 |
| A02372 | 3 | 3 | 0 |
| A02373 | 3 | 3 | 0 |
| A02375 | 3 | 3 | 0 |
| A02376 | 3 | 1 | 2 |
| A02377 | 3 | 2 | 1 |
| A02378 | 3 | 2 | 1 |
| A02379 | 3 | 2 | 1 |
| A02381 | 3 | 1 | 2 |
| A02383 | 3 | 2 | 1 |
| A02384 | 3 | 3 | 0 |
| A02385 | 3 | 3 | 0 |
| A02387 | 3 | 1 | 2 |
| A02388 | 3 | 1 | 2 |
| A02389 | 3 | 3 | 0 |
| A02391 | 3 | 3 | 0 |
| A02392 | 3 | 3 | 0 |
| A02393 | 3 | 1 | 2 |
| A02394 | 3 | 2 | 1 |
| A02395 | 3 | 1 | 2 |
| A02396 | 3 | 2 | 1 |
| A02397 | 3 | 1 | 2 |
| A02398 | 3 | 3 | 0 |
| A02399 | 3 | 1 | 2 |
| A02400 | 3 | 1 | 2 |
| A02401 | 3 | 3 | 0 |
| A02402 | 3 | 1 | 2 |
| A02403 | 3 | 1 | 2 |
| A02404 | 3 | 3 | 0 |
| A02405 | 3 | 1 | 2 |
| A02406 | 3 | 1 | 2 |
| A02408 | 3 | 1 | 2 |
| A02409 | 3 | 1 | 2 |
| A02411 | 3 | 1 | 2 |
| A02412 | 3 | 3 | 0 |
| A02413 | 3 | 2 | 1 |
| A02414 | 3 | 3 | 0 |
| A02416 | 3 | 2 | 1 |
| A02417 | 3 | 2 | 1 |
| A02418 | 3 | 2 | 1 |
| A02420 | 3 | 3 | 0 |
| A02421 | 3 | 1 | 2 |
| A02422 | 3 | 3 | 0 |
| A02423 | 3 | 2 | 1 |
| A02424 | 3 | 1 | 2 |
| A02425 | 3 | 1 | 2 |
| A02426 | 3 | 1 | 2 |
| A02427 | 3 | 1 | 2 |
| A02428 | 3 | 3 | 0 |
| A02429 | 3 | 1 | 2 |
| A02430 | 3 | 1 | 2 |
| A02431 | 3 | 1 | 2 |
| A02432 | 3 | 1 | 2 |
| A02433 | 3 | 1 | 2 |
| A02434 | 3 | 1 | 2 |
| A02435 | 3 | 1 | 2 |
| A02436 | 3 | 1 | 2 |
| A02437 | 3 | 1 | 2 |
| A02438 | 3 | 3 | 0 |
| A02439 | 3 | 1 | 2 |
| A02440 | 3 | 1 | 2 |
| A02441 | 3 | 1 | 2 |
| A02442 | 3 | 1 | 2 |
| A02443 | 3 | 2 | 1 |
| A02444 | 3 | 1 | 2 |
| A02445 | 3 | 1 | 2 |
| A02446 | 3 | 3 | 0 |
| A02447 | 3 | 1 | 2 |
| A02448 | 3 | 1 | 2 |
| A02449 | 3 | 1 | 2 |
| A02450 | 3 | 3 | 0 |
| A02451 | 3 | 1 | 2 |
| A02452 | 3 | 1 | 2 |
| A02453 | 3 | 2 | 1 |
| A02454 | 3 | 1 | 2 |
| A02455 | 3 | 1 | 2 |
| A02456 | 3 | 1 | 2 |
| A02457 | 3 | 1 | 2 |
| A02458 | 3 | 2 | 1 |
| A02459 | 3 | 2 | 1 |
| A02460 | 3 | 1 | 2 |
| A02461 | 3 | 1 | 2 |
| A02462 | 3 | 1 | 2 |
| A02463 | 3 | 2 | 1 |
| A02464 | 3 | 2 | 1 |
| A02465 | 3 | 3 | 0 |
| A02466 | 3 | 1 | 2 |
| A02467 | 3 | 3 | 0 |
| A02468 | 3 | 1 | 2 |
| A02469 | 3 | 1 | 2 |
| A02470 | 3 | 2 | 1 |
| A02471 | 3 | 1 | 2 |
| A02472 | 3 | 1 | 2 |
| A02473 | 3 | 2 | 1 |
| A02474 | 3 | 3 | 0 |
| A02475 | 3 | 3 | 0 |
| A02476 | 3 | 3 | 0 |
| A02477 | 3 | 3 | 0 |
| A02478 | 3 | 1 | 2 |
| A02483 | 3 | 1 | 2 |
| A02484 | 3 | 1 | 2 |
| A02485 | 3 | 1 | 2 |
| A02486 | 3 | 2 | 1 |
| A02487 | 3 | 2 | 1 |
| A02488 | 3 | 1 | 2 |
| A02489 | 3 | 1 | 2 |
| A02490 | 3 | 2 | 1 |
| A02491 | 3 | 1 | 2 |
| A02492 | 3 | 1 | 2 |
| A02493 | 3 | 1 | 2 |
| A02494 | 3 | 1 | 2 |
| A02495 | 3 | 2 | 1 |
| A02496 | 3 | 1 | 2 |
| A02497 | 3 | 1 | 2 |
| A02498 | 3 | 1 | 2 |
| A02499 | 3 | 2 | 1 |
| A02501 | 3 | 1 | 2 |
| A02502 | 3 | 1 | 2 |
| A02504 | 3 | 1 | 2 |
| A02505 | 3 | 1 | 2 |
| A02506 | 3 | 3 | 0 |
| A02507 | 3 | 1 | 2 |
| A02509 | 3 | 1 | 2 |
| A02510 | 3 | 2 | 1 |
| A02511 | 3 | 2 | 1 |
| A02512 | 3 | 3 | 0 |
| A02513 | 3 | 1 | 2 |
| A02514 | 3 | 1 | 2 |
| A02515 | 3 | 1 | 2 |
| A02516 | 3 | 3 | 0 |
| A02517 | 3 | 1 | 2 |
| A02518 | 3 | 1 | 2 |
| A02519 | 3 | 3 | 0 |
| A02520 | 3 | 2 | 1 |
| A02522 | 3 | 1 | 2 |
| A02523 | 3 | 1 | 2 |
| A02524 | 3 | 2 | 1 |
| A02525 | 3 | 2 | 1 |
| A02526 | 3 | 1 | 2 |
| A02527 | 3 | 1 | 2 |
| A02528 | 3 | 1 | 2 |
| A02529 | 3 | 1 | 2 |
| A02530 | 3 | 3 | 0 |
| A02531 | 3 | 3 | 0 |
| A02532 | 3 | 1 | 2 |
| A02533 | 3 | 1 | 2 |
| A02534 | 3 | 1 | 2 |
| A02535 | 3 | 1 | 2 |
| A02536 | 3 | 1 | 2 |
| A02538 | 3 | 3 | 0 |
| A02539 | 3 | 3 | 0 |
| A02540 | 3 | 1 | 2 |
| A02541 | 3 | 3 | 0 |
| A02542 | 3 | 1 | 2 |
| A02543 | 3 | 1 | 2 |
| A02544 | 3 | 1 | 2 |
| A02545 | 3 | 3 | 0 |
| A02546 | 3 | 3 | 0 |
| A02548 | 3 | 3 | 0 |
| A02549 | 3 | 1 | 2 |
| A02550 | 3 | 1 | 2 |
| A02551 | 3 | 3 | 0 |
| A02552 | 3 | 3 | 0 |
| A02553 | 3 | 3 | 0 |
| A02554 | 3 | 3 | 0 |
| A02555 | 3 | 1 | 2 |
| A02556 | 3 | 1 | 2 |
| A02557 | 3 | 2 | 1 |
| A02558 | 3 | 3 | 0 |
| A02559 | 3 | 1 | 2 |
| A02560 | 3 | 1 | 2 |
| A02562 | 3 | 1 | 2 |
| A02563 | 3 | 1 | 2 |
| A02564 | 3 | 1 | 2 |
| A02565 | 3 | 1 | 2 |
| A02566 | 3 | 1 | 2 |
| A02567 | 3 | 1 | 2 |
| A02568 | 3 | 3 | 0 |
| A02569 | 3 | 3 | 0 |
| A02570 | 3 | 1 | 2 |
| A02571 | 3 | 1 | 2 |
| A02572 | 3 | 3 | 0 |
| A02573 | 3 | 1 | 2 |
| A02574 | 3 | 1 | 2 |
| A02575 | 3 | 2 | 1 |
| A02576 | 3 | 1 | 2 |
| A02577 | 3 | 3 | 0 |
| A02578 | 3 | 2 | 1 |
| A02579 | 3 | 1 | 2 |
| A02580 | 3 | 1 | 2 |
| A02581 | 3 | 3 | 0 |
| A02583 | 3 | 1 | 2 |
| A02584 | 3 | 3 | 0 |
| A02585 | 3 | 1 | 2 |
| A02586 | 3 | 3 | 0 |
| A02587 | 3 | 1 | 2 |
| A02588 | 3 | 1 | 2 |
| A02589 | 3 | 3 | 0 |
| A02591 | 3 | 1 | 2 |
| A02592 | 3 | 3 | 0 |
| A02594 | 3 | 3 | 0 |
| A02595 | 3 | 2 | 1 |
| A02596 | 3 | 1 | 2 |
| A02597 | 3 | 3 | 0 |
| A02598 | 3 | 3 | 0 |
| A02599 | 3 | 2 | 1 |
| A02600 | 3 | 3 | 0 |
| A02601 | 3 | 2 | 1 |
| A02602 | 3 | 3 | 0 |
| A02603 | 3 | 3 | 0 |
| A02604 | 3 | 3 | 0 |
| A02605 | 3 | 3 | 0 |
| A02606 | 3 | 3 | 0 |
| A02607 | 3 | 3 | 0 |
| A02608 | 3 | 3 | 0 |
| A02609 | 3 | 3 | 0 |
| A02610 | 3 | 3 | 0 |
| A02611 | 3 | 1 | 2 |
| A02612 | 3 | 1 | 2 |
| A02613 | 3 | 3 | 0 |
| A02614 | 3 | 3 | 0 |
| A02615 | 3 | 3 | 0 |
| A02616 | 3 | 3 | 0 |
| A02617 | 3 | 1 | 2 |
| A02618 | 3 | 2 | 1 |
| A02619 | 3 | 2 | 1 |
| A02620 | 3 | 1 | 2 |
| A02621 | 3 | 1 | 2 |
| A02622 | 3 | 2 | 1 |
| A02623 | 3 | 2 | 1 |
| A02624 | 3 | 2 | 1 |
| A02625 | 3 | 2 | 1 |
| A02626 | 3 | 2 | 1 |
| A02628 | 3 | 2 | 1 |
| A02629 | 3 | 1 | 2 |
| A02630 | 3 | 1 | 2 |
| A02634 | 3 | 2 | 1 |
| A02635 | 3 | 2 | 1 |
| A02637 | 3 | 2 | 1 |
| A02638 | 3 | 3 | 0 |
| A02639 | 3 | 3 | 0 |
| A02640 | 3 | 1 | 2 |
| A02641 | 3 | 2 | 1 |
| A02642 | 3 | 2 | 1 |
| A02643 | 3 | 2 | 1 |
| A02644 | 3 | 2 | 1 |
| A02645 | 3 | 2 | 1 |
| A02646 | 3 | 2 | 1 |
| A02647 | 3 | 1 | 2 |
| A02648 | 3 | 2 | 1 |
| A02649 | 3 | 2 | 1 |
| A02650 | 3 | 2 | 1 |
| A02651 | 3 | 2 | 1 |
| A02654 | 3 | 1 | 2 |
| A02655 | 3 | 1 | 2 |
| A02656 | 3 | 3 | 0 |
| A02657 | 3 | 3 | 0 |
| A02658 | 3 | 3 | 0 |
| A02659 | 3 | 3 | 0 |
| A02660 | 3 | 3 | 0 |
| A02661 | 3 | 2 | 1 |
| A02662 | 3 | 1 | 2 |
| A02663 | 3 | 1 | 2 |
| A02664 | 3 | 3 | 0 |
| A02665 | 3 | 2 | 1 |
| A02666 | 3 | 2 | 1 |
| A02667 | 3 | 3 | 0 |
| A02668 | 3 | 2 | 1 |
| A02669 | 3 | 3 | 0 |
| A02670 | 3 | 2 | 1 |
| A02671 | 3 | 2 | 1 |
| A02672 | 3 | 1 | 2 |
| A02674 | 3 | 2 | 1 |
| A02675 | 3 | 2 | 1 |
| A02676 | 3 | 1 | 2 |
| A02678 | 3 | 1 | 2 |
| A02679 | 3 | 3 | 0 |
| A02680 | 3 | 3 | 0 |
| A02681 | 3 | 3 | 0 |
| A02682 | 3 | 2 | 1 |
| A02683 | 3 | 3 | 0 |
| A02684 | 3 | 3 | 0 |
| A02685 | 3 | 1 | 2 |
| A02686 | 3 | 2 | 1 |
| A02687 | 3 | 1 | 2 |
| A02688 | 3 | 1 | 2 |
| A02689 | 3 | 1 | 2 |
| A02690 | 3 | 3 | 0 |
| A02691 | 3 | 3 | 0 |
| A02692 | 3 | 1 | 2 |
| A02693 | 3 | 1 | 2 |
| A02694 | 3 | 2 | 1 |
| A02695 | 3 | 1 | 2 |
| A02696 | 3 | 1 | 2 |
| A02697 | 3 | 2 | 1 |
| A02698 | 3 | 2 | 1 |
| A02701 | 3 | 2 | 1 |
| A02702 | 3 | 3 | 0 |
| A02704 | 3 | 1 | 2 |
| A02705 | 3 | 2 | 1 |
| A02706 | 3 | 1 | 2 |
| A02707 | 3 | 1 | 2 |
| A02708 | 3 | 1 | 2 |
| A02709 | 3 | 1 | 2 |
| A02710 | 3 | 2 | 1 |
| A02712 | 3 | 1 | 2 |
| A02713 | 3 | 2 | 1 |
| A02714 | 3 | 1 | 2 |
| A02718 | 3 | 1 | 2 |
| A02719 | 3 | 1 | 2 |
| A02720 | 3 | 2 | 1 |
| A02721 | 3 | 3 | 0 |
| A02722 | 3 | 1 | 2 |
| A02723 | 3 | 1 | 2 |
| A02724 | 3 | 1 | 2 |
| A02725 | 3 | 1 | 2 |
| A02726 | 3 | 3 | 0 |
| A02727 | 3 | 2 | 1 |
| A02729 | 3 | 3 | 0 |
| A02730 | 3 | 2 | 1 |
| A02731 | 3 | 3 | 0 |
| A02732 | 3 | 3 | 0 |
| A02733 | 3 | 3 | 0 |
| A02734 | 3 | 3 | 0 |
| A02735 | 3 | 3 | 0 |
| A02736 | 3 | 1 | 2 |
| A02737 | 3 | 1 | 2 |
| A02738 | 3 | 1 | 2 |
| A02739 | 3 | 3 | 0 |
| A02740 | 3 | 3 | 0 |
| A02741 | 3 | 3 | 0 |
| A02742 | 3 | 3 | 0 |
| A02743 | 3 | 1 | 2 |
| A02744 | 3 | 2 | 1 |
| A02745 | 3 | 3 | 0 |
| A02746 | 3 | 3 | 0 |
| A02747 | 3 | 1 | 2 |
| A02748 | 3 | 3 | 0 |
| A02749 | 3 | 2 | 1 |
| A02750 | 3 | 3 | 0 |
| A02751 | 3 | 2 | 1 |
| A02752 | 3 | 3 | 0 |
| A02753 | 3 | 2 | 1 |
| A02754 | 3 | 1 | 2 |
| A02755 | 3 | 3 | 0 |
| A02756 | 3 | 3 | 0 |
| A02757 | 3 | 3 | 0 |
| A02758 | 3 | 1 | 2 |
| A02759 | 3 | 2 | 1 |
| A02760 | 3 | 2 | 1 |
| A02761 | 3 | 2 | 1 |
| A02762 | 3 | 2 | 1 |
| A02763 | 3 | 1 | 2 |
| A02764 | 3 | 1 | 2 |
| A02765 | 3 | 1 | 2 |
| A02766 | 3 | 3 | 0 |
| A02767 | 3 | 3 | 0 |
| A02768 | 3 | 1 | 2 |
| A02769 | 3 | 3 | 0 |
| A02770 | 3 | 3 | 0 |
| A02771 | 3 | 3 | 0 |
| A02772 | 3 | 1 | 2 |
| A02773 | 3 | 1 | 2 |
| A02774 | 3 | 1 | 2 |
| A02775 | 3 | 1 | 2 |
| A02778 | 3 | 1 | 2 |
| A02780 | 3 | 1 | 2 |
| A02781 | 3 | 1 | 2 |
| A02782 | 3 | 1 | 2 |
| A02783 | 3 | 1 | 2 |
| A02784 | 3 | 1 | 2 |
| A02786 | 3 | 1 | 2 |
| A02787 | 3 | 3 | 0 |
| A02788 | 3 | 1 | 2 |
| A02789 | 3 | 1 | 2 |
| A02791 | 3 | 1 | 2 |
| A02792 | 3 | 3 | 0 |
| A02793 | 3 | 1 | 2 |
| A02794 | 3 | 2 | 1 |
| A02795 | 3 | 3 | 0 |
| A02796 | 3 | 3 | 0 |
| A02797 | 3 | 1 | 2 |
| A02798 | 3 | 1 | 2 |
| A02799 | 3 | 1 | 2 |
| A02801 | 3 | 1 | 2 |
| A02802 | 3 | 2 | 1 |
| A02803 | 3 | 3 | 0 |
| A02804 | 3 | 2 | 1 |
| A02805 | 3 | 1 | 2 |
| A02806 | 3 | 2 | 1 |
| A02807 | 3 | 3 | 0 |
| A02808 | 3 | 3 | 0 |
| A02809 | 3 | 3 | 0 |
| A02810 | 3 | 1 | 2 |
| A02811 | 3 | 1 | 2 |
| A02812 | 3 | 1 | 2 |
| A02813 | 3 | 3 | 0 |
| A02814 | 3 | 3 | 0 |
| A02815 | 3 | 2 | 1 |
| A02816 | 3 | 2 | 1 |
| A02817 | 3 | 1 | 2 |
| A02818 | 3 | 3 | 0 |
| A02819 | 3 | 3 | 0 |
| A02820 | 3 | 3 | 0 |
| A02821 | 3 | 3 | 0 |
| A02822 | 3 | 2 | 1 |
| A02823 | 3 | 2 | 1 |
| A02824 | 3 | 3 | 0 |
| A02825 | 3 | 1 | 2 |
| A02826 | 3 | 3 | 0 |
| A02827 | 3 | 1 | 2 |
| A02828 | 3 | 1 | 2 |
| A02829 | 3 | 1 | 2 |
| A02830 | 3 | 1 | 2 |
| A02832 | 3 | 3 | 0 |
| A02833 | 3 | 1 | 2 |
| A02834 | 3 | 1 | 2 |
| A02836 | 3 | 3 | 0 |
| A02837 | 3 | 1 | 2 |
| A02838 | 3 | 1 | 2 |
| A02839 | 3 | 1 | 2 |
| A02840 | 3 | 1 | 2 |
| A02841 | 3 | 2 | 1 |
| A02842 | 3 | 1 | 2 |
| A02843 | 3 | 1 | 2 |
| A02844 | 3 | 1 | 2 |
| A02845 | 3 | 1 | 2 |
| A02846 | 3 | 2 | 1 |
| A02847 | 3 | 1 | 2 |
| A02848 | 3 | 1 | 2 |
| A02851 | 3 | 1 | 2 |
| A02853 | 3 | 3 | 0 |
| A02855 | 3 | 1 | 2 |
| A02856 | 3 | 1 | 2 |
| A02857 | 3 | 1 | 2 |
| A02859 | 3 | 3 | 0 |
| A02860 | 3 | 2 | 1 |
| A02861 | 3 | 2 | 1 |
| A02862 | 3 | 3 | 0 |
| A02864 | 3 | 1 | 2 |
| A02866 | 3 | 3 | 0 |
| A02867 | 3 | 1 | 2 |
| A02868 | 3 | 1 | 2 |
| A02869 | 3 | 1 | 2 |
| A02870 | 3 | 3 | 0 |
| A02871 | 3 | 3 | 0 |
| A02872 | 3 | 3 | 0 |
| A02873 | 3 | 1 | 2 |
| A02874 | 3 | 3 | 0 |
| A02875 | 3 | 1 | 2 |
| A02876 | 3 | 1 | 2 |
| A02877 | 3 | 3 | 0 |
| A02878 | 3 | 1 | 2 |
| A02879 | 3 | 1 | 2 |
| A02880 | 3 | 1 | 2 |
| A02881 | 3 | 1 | 2 |
| A02882 | 3 | 3 | 0 |
| A02883 | 3 | 3 | 0 |
| A02884 | 3 | 3 | 0 |
| A02885 | 3 | 1 | 2 |
| A02886 | 3 | 3 | 0 |
| A02887 | 3 | 3 | 0 |
| A02888 | 3 | 1 | 2 |
| A02889 | 3 | 3 | 0 |
| A02890 | 3 | 1 | 2 |
| A02891 | 3 | 1 | 2 |
| A02892 | 3 | 3 | 0 |
| A02893 | 3 | 1 | 2 |
| A02894 | 3 | 1 | 2 |
| A02895 | 3 | 3 | 0 |
| A02896 | 3 | 1 | 2 |
| A02897 | 3 | 1 | 2 |
| A02898 | 3 | 3 | 0 |
| A02899 | 3 | 3 | 0 |
| A02900 | 3 | 2 | 1 |
| A02901 | 3 | 3 | 0 |
| A02902 | 3 | 1 | 2 |
| A02903 | 3 | 1 | 2 |
| A02904 | 3 | 1 | 2 |
| A02905 | 3 | 1 | 2 |
| A02906 | 3 | 1 | 2 |
| A02907 | 3 | 1 | 2 |
| A02909 | 3 | 1 | 2 |
| A02910 | 3 | 2 | 1 |
| A02911 | 3 | 2 | 1 |
| A02913 | 3 | 2 | 1 |
| A02921 | 3 | 2 | 1 |
| A02922 | 3 | 2 | 1 |
| A02923 | 3 | 2 | 1 |
| A02924 | 3 | 2 | 1 |
| A02925 | 3 | 2 | 1 |
| A02926 | 3 | 2 | 1 |
| A02927 | 3 | 2 | 1 |
| A02928 | 3 | 2 | 1 |
| A02930 | 3 | 1 | 2 |
| A02931 | 3 | 2 | 1 |
| A02933 | 3 | 1 | 2 |
| A02935 | 3 | 2 | 1 |
| A02938 | 3 | 2 | 1 |
| A02939 | 3 | 2 | 1 |
| A02940 | 3 | 2 | 1 |
| A02941 | 3 | 2 | 1 |
| A02944 | 3 | 2 | 1 |
| A02945 | 3 | 2 | 1 |
| A02948 | 3 | 1 | 2 |
| A02950 | 3 | 1 | 2 |
| A02951 | 3 | 2 | 1 |
| A02957 | 3 | 2 | 1 |
| A02958 | 3 | 1 | 2 |
| A02962 | 3 | 2 | 1 |
| A02964 | 3 | 1 | 2 |
| A02966 | 3 | 2 | 1 |
| A02967 | 3 | 2 | 1 |
| A02969 | 3 | 2 | 1 |
| A02972 | 3 | 1 | 2 |
| A02973 | 3 | 2 | 1 |
| A02975 | 3 | 2 | 1 |
| A02976 | 3 | 2 | 1 |
| A02982 | 3 | 2 | 1 |
| A02983 | 3 | 2 | 1 |
| A02986 | 3 | 2 | 1 |
| A02987 | 3 | 1 | 2 |
| A02988 | 3 | 2 | 1 |
| A02990 | 3 | 2 | 1 |
| A02991 | 3 | 2 | 1 |
| A02994 | 3 | 2 | 1 |
| A02995 | 3 | 2 | 1 |
| A02998 | 3 | 2 | 1 |
| A02999 | 3 | 2 | 1 |
| A03000 | 3 | 1 | 2 |
| A03001 | 3 | 3 | 0 |
| A03002 | 3 | 3 | 0 |
| A03003 | 3 | 1 | 2 |
| A03004 | 3 | 3 | 0 |
| A03005 | 3 | 3 | 0 |
| A03006 | 3 | 1 | 2 |
| A03007 | 3 | 1 | 2 |
| A03008 | 3 | 2 | 1 |
| A03009 | 3 | 1 | 2 |
| A03010 | 3 | 1 | 2 |
| A03011 | 3 | 1 | 2 |
| A03012 | 3 | 1 | 2 |
| A03013 | 3 | 1 | 2 |
| A03014 | 3 | 3 | 0 |
| A03015 | 3 | 3 | 0 |
| A03016 | 3 | 3 | 0 |
| A03017 | 3 | 1 | 2 |
| A03018 | 3 | 1 | 2 |
| A03019 | 3 | 1 | 2 |
| A03020 | 3 | 1 | 2 |
| A03021 | 3 | 1 | 2 |
| A03022 | 3 | 1 | 2 |
| A03023 | 3 | 2 | 1 |
| A03024 | 3 | 1 | 2 |
| A03025 | 3 | 1 | 2 |
| A03026 | 3 | 2 | 1 |
| A03027 | 3 | 3 | 0 |
| A03028 | 3 | 2 | 1 |
| A03029 | 3 | 3 | 0 |
| A03030 | 3 | 3 | 0 |
| A03031 | 3 | 3 | 0 |
| A03032 | 3 | 3 | 0 |
| A03033 | 3 | 2 | 1 |
| A03034 | 3 | 1 | 2 |
| A03035 | 3 | 1 | 2 |
| A03036 | 3 | 3 | 0 |
| A03037 | 3 | 1 | 2 |
| A03038 | 3 | 3 | 0 |
| A03039 | 3 | 3 | 0 |
| A03041 | 3 | 1 | 2 |
| A03042 | 3 | 1 | 2 |
| A03043 | 3 | 1 | 2 |
| A03044 | 3 | 1 | 2 |
| A03045 | 3 | 3 | 0 |
| A03046 | 3 | 3 | 0 |
| A03047 | 3 | 3 | 0 |
| A03048 | 3 | 2 | 1 |
| A03049 | 3 | 2 | 1 |
| A03050 | 3 | 3 | 0 |
| A03051 | 3 | 2 | 1 |
| A03052 | 3 | 1 | 2 |
| A03053 | 3 | 1 | 2 |
| A03054 | 3 | 2 | 1 |
| A03055 | 3 | 2 | 1 |
| A03056 | 3 | 2 | 1 |
| A03057 | 3 | 2 | 1 |
| A03058 | 3 | 1 | 2 |
| A03059 | 3 | 1 | 2 |
| A03060 | 3 | 1 | 2 |
| A03061 | 3 | 1 | 2 |
| A03062 | 3 | 2 | 1 |
| A03063 | 3 | 2 | 1 |
| A03064 | 3 | 3 | 0 |
| A03065 | 3 | 3 | 0 |
| A03066 | 3 | 3 | 0 |
| A03067 | 3 | 3 | 0 |
| A03068 | 3 | 3 | 0 |
| A03069 | 3 | 1 | 2 |
| A03070 | 3 | 2 | 1 |
| A03071 | 3 | 3 | 0 |
| A03072 | 3 | 3 | 0 |
| A03073 | 3 | 3 | 0 |
| A03074 | 3 | 1 | 2 |
| A03075 | 3 | 1 | 2 |
| A03076 | 3 | 1 | 2 |
| A03077 | 3 | 3 | 0 |
| A03078 | 3 | 2 | 1 |
| A03079 | 3 | 1 | 2 |
| A03080 | 3 | 1 | 2 |
| A03081 | 3 | 1 | 2 |
| A03082 | 3 | 1 | 2 |
| A03083 | 3 | 2 | 1 |
| A03084 | 3 | 3 | 0 |
| A03085 | 3 | 1 | 2 |
| A03086 | 3 | 2 | 1 |
| A03087 | 3 | 1 | 2 |
| A03088 | 3 | 1 | 2 |
| A03089 | 3 | 1 | 2 |
| A03090 | 3 | 2 | 1 |
| A03091 | 3 | 1 | 2 |
| A03092 | 3 | 1 | 2 |
| A03093 | 3 | 1 | 2 |
| A03094 | 3 | 1 | 2 |
| A03095 | 3 | 1 | 2 |
| A03096 | 3 | 1 | 2 |
| A03097 | 3 | 2 | 1 |
| A03098 | 3 | 1 | 2 |
| A03099 | 3 | 1 | 2 |
| A03100 | 3 | 1 | 2 |
| A03101 | 3 | 1 | 2 |
| A03102 | 3 | 1 | 2 |
| A03103 | 3 | 1 | 2 |
| A03104 | 3 | 1 | 2 |
| A03105 | 3 | 2 | 1 |
| A03106 | 3 | 1 | 2 |
| A03107 | 3 | 1 | 2 |
| A03108 | 3 | 1 | 2 |
| A03109 | 3 | 1 | 2 |
| A03110 | 3 | 1 | 2 |
| A03111 | 3 | 1 | 2 |
| A03112 | 3 | 1 | 2 |
| A03114 | 3 | 1 | 2 |
| A03115 | 3 | 1 | 2 |
| A03116 | 3 | 3 | 0 |
| A03117 | 3 | 3 | 0 |
| A03118 | 3 | 2 | 1 |
| A03119 | 3 | 2 | 1 |
| A03120 | 3 | 2 | 1 |
| A03121 | 3 | 1 | 2 |
| A03122 | 3 | 1 | 2 |
| A03123 | 3 | 1 | 2 |
| A03124 | 3 | 1 | 2 |
| A03126 | 3 | 1 | 2 |
| A03127 | 3 | 1 | 2 |
| A03128 | 3 | 2 | 1 |
| A03129 | 3 | 2 | 1 |
| A03130 | 3 | 2 | 1 |
| A03131 | 3 | 1 | 2 |
| A03132 | 3 | 3 | 0 |
| A03133 | 3 | 3 | 0 |
| A03134 | 3 | 2 | 1 |
| A03135 | 3 | 2 | 1 |
| A03136 | 3 | 1 | 2 |
| A03137 | 3 | 1 | 2 |
| A03138 | 3 | 3 | 0 |
| A03139 | 3 | 1 | 2 |
| A03141 | 3 | 1 | 2 |
| A03142 | 3 | 3 | 0 |
| A03144 | 3 | 1 | 2 |
| A03145 | 3 | 1 | 2 |
| A03146 | 3 | 3 | 0 |
| A03147 | 3 | 1 | 2 |
| A03148 | 3 | 1 | 2 |
| A03149 | 3 | 3 | 0 |
| A03150 | 3 | 1 | 2 |
| A03151 | 3 | 1 | 2 |
| A03152 | 3 | 1 | 2 |
| A03153 | 3 | 1 | 2 |
| A03154 | 3 | 1 | 2 |
| A03155 | 3 | 1 | 2 |
| A03156 | 3 | 1 | 2 |
| A03157 | 3 | 1 | 2 |
| A03158 | 3 | 1 | 2 |
| A03161 | 3 | 1 | 2 |
| A03162 | 3 | 1 | 2 |
| A03163 | 3 | 1 | 2 |
| A03164 | 3 | 2 | 1 |
| A03165 | 3 | 3 | 0 |
| A03166 | 3 | 1 | 2 |
| A03167 | 3 | 1 | 2 |
| A03169 | 3 | 3 | 0 |
| A03170 | 3 | 2 | 1 |
| A03171 | 3 | 2 | 1 |
| A03172 | 3 | 1 | 2 |
| A03173 | 3 | 1 | 2 |
| A03174 | 3 | 1 | 2 |
| A03175 | 3 | 1 | 2 |
| A03176 | 3 | 1 | 2 |
| A03177 | 3 | 1 | 2 |
| A03178 | 3 | 2 | 1 |
| A03179 | 3 | 1 | 2 |
| A03180 | 3 | 2 | 1 |
| A03181 | 3 | 3 | 0 |
| A03182 | 3 | 1 | 2 |
| A03183 | 3 | 3 | 0 |
| A03185 | 3 | 1 | 2 |
| A03186 | 3 | 2 | 1 |
| A03187 | 3 | 3 | 0 |
| A03189 | 3 | 1 | 2 |
| A03190 | 3 | 3 | 0 |
| A03191 | 3 | 3 | 0 |
| A03192 | 3 | 3 | 0 |
| A03193 | 3 | 1 | 2 |
| A03194 | 3 | 2 | 1 |
| A03195 | 3 | 1 | 2 |
| A03196 | 3 | 1 | 2 |
| A03197 | 3 | 1 | 2 |
| A03198 | 3 | 1 | 2 |
| A03199 | 3 | 1 | 2 |
| A03200 | 3 | 1 | 2 |
| A03201 | 3 | 1 | 2 |
| A03202 | 3 | 3 | 0 |
| A03203 | 3 | 3 | 0 |
| A03204 | 3 | 3 | 0 |
| A03205 | 3 | 3 | 0 |
| A03206 | 3 | 3 | 0 |
| A03207 | 3 | 3 | 0 |
| A03208 | 3 | 3 | 0 |
| A03209 | 3 | 2 | 1 |
| A03210 | 3 | 2 | 1 |
| A03211 | 3 | 2 | 1 |
| A03213 | 3 | 3 | 0 |
| A03214 | 3 | 3 | 0 |
| A03215 | 3 | 2 | 1 |
| A03216 | 3 | 3 | 0 |
| A03218 | 3 | 1 | 2 |
| A03219 | 3 | 1 | 2 |
| A03220 | 3 | 1 | 2 |
| A03221 | 3 | 1 | 2 |
| A03222 | 3 | 1 | 2 |
| A03223 | 3 | 1 | 2 |
| A03224 | 3 | 2 | 1 |
| A03225 | 3 | 3 | 0 |
| A03228 | 3 | 1 | 2 |
| A03229 | 3 | 3 | 0 |
| A03230 | 3 | 3 | 0 |
| A03231 | 3 | 3 | 0 |
| A03232 | 3 | 1 | 2 |
| A03233 | 3 | 1 | 2 |
| A03234 | 3 | 3 | 0 |
| A03235 | 3 | 1 | 2 |
| A03236 | 3 | 1 | 2 |
| A03237 | 3 | 2 | 1 |
| A03238 | 3 | 2 | 1 |
| A03239 | 3 | 3 | 0 |
| A03240 | 3 | 3 | 0 |
| A03241 | 3 | 1 | 2 |
| A03242 | 3 | 3 | 0 |
| A03243 | 3 | 3 | 0 |
| A03245 | 3 | 2 | 1 |
| A03248 | 3 | 1 | 2 |
| A03249 | 3 | 1 | 2 |
| A03251 | 3 | 1 | 2 |
| A03252 | 3 | 1 | 2 |
| A03253 | 3 | 1 | 2 |
| A03254 | 3 | 2 | 1 |
| A03255 | 3 | 1 | 2 |
| A03256 | 3 | 1 | 2 |
| A03257 | 3 | 2 | 1 |
| A03258 | 3 | 3 | 0 |
| A03259 | 3 | 2 | 1 |
| A03260 | 3 | 2 | 1 |
| A03261 | 3 | 2 | 1 |
| A03262 | 3 | 3 | 0 |
| A03263 | 3 | 3 | 0 |
| A03264 | 3 | 3 | 0 |
| A03267 | 3 | 1 | 2 |
| A03268 | 3 | 2 | 1 |
| A03269 | 3 | 1 | 2 |
| A03270 | 3 | 3 | 0 |
| A03271 | 3 | 1 | 2 |
| A03272 | 3 | 3 | 0 |
| A03273 | 3 | 1 | 2 |
| A03274 | 3 | 1 | 2 |
| A03275 | 3 | 3 | 0 |
| A03276 | 3 | 1 | 2 |
| A03277 | 3 | 1 | 2 |
| A03278 | 3 | 3 | 0 |
| A03279 | 3 | 3 | 0 |
| A03280 | 3 | 3 | 0 |
| A03281 | 3 | 3 | 0 |
| A03282 | 3 | 1 | 2 |
| A03283 | 3 | 1 | 2 |
| A03284 | 3 | 1 | 2 |
| A03285 | 3 | 1 | 2 |
| A03286 | 3 | 1 | 2 |
| A03287 | 3 | 3 | 0 |
| A03288 | 3 | 2 | 1 |
| A03289 | 3 | 1 | 2 |
| A03290 | 3 | 1 | 2 |
| A03291 | 3 | 3 | 0 |
| A03292 | 3 | 3 | 0 |
| A03294 | 3 | 1 | 2 |
| A03295 | 3 | 1 | 2 |
| A03296 | 3 | 3 | 0 |
| A03297 | 3 | 3 | 0 |
| A03298 | 3 | 3 | 0 |
| A03299 | 3 | 3 | 0 |
| A03300 | 3 | 3 | 0 |
| A03301 | 3 | 3 | 0 |
| A03302 | 3 | 2 | 1 |
| A03303 | 3 | 3 | 0 |
| A03304 | 3 | 3 | 0 |
| A03305 | 3 | 3 | 0 |
| A03306 | 3 | 3 | 0 |
| A03307 | 3 | 3 | 0 |
| A03308 | 3 | 3 | 0 |
| A03309 | 3 | 3 | 0 |
| A03310 | 3 | 1 | 2 |
| A03311 | 3 | 1 | 2 |
| A03312 | 3 | 1 | 2 |
| A03313 | 3 | 1 | 2 |
| A03314 | 3 | 1 | 2 |
| A03315 | 3 | 3 | 0 |
| A03316 | 3 | 3 | 0 |
| A03317 | 3 | 3 | 0 |
| A03318 | 3 | 2 | 1 |
| A03319 | 3 | 1 | 2 |
| A03320 | 3 | 1 | 2 |
| A03321 | 3 | 2 | 1 |
| A03323 | 3 | 1 | 2 |
| A03324 | 3 | 1 | 2 |
| A03325 | 3 | 3 | 0 |
| A03326 | 3 | 1 | 2 |
| A03327 | 3 | 3 | 0 |
| A03328 | 3 | 3 | 0 |
| A03329 | 3 | 1 | 2 |
| A03330 | 3 | 3 | 0 |
| A03331 | 3 | 1 | 2 |
| A03332 | 3 | 3 | 0 |
| A03333 | 3 | 1 | 2 |
| A03334 | 3 | 3 | 0 |
| A03335 | 3 | 1 | 2 |
| A03336 | 3 | 3 | 0 |
| A03337 | 3 | 3 | 0 |
| A03338 | 3 | 3 | 0 |
| A03339 | 3 | 1 | 2 |
| A03341 | 3 | 1 | 2 |
| A03342 | 3 | 3 | 0 |
| A03343 | 3 | 3 | 0 |
| A03345 | 3 | 1 | 2 |
| A03346 | 3 | 3 | 0 |
| A03347 | 3 | 3 | 0 |
| A03348 | 3 | 3 | 0 |
| A03349 | 3 | 3 | 0 |
| A03350 | 3 | 3 | 0 |
| A03351 | 3 | 3 | 0 |
| A03352 | 3 | 3 | 0 |
| A03353 | 3 | 2 | 1 |
| A03354 | 3 | 1 | 2 |
| A03355 | 3 | 1 | 2 |
| A03356 | 3 | 1 | 2 |
| A03357 | 3 | 1 | 2 |
| A03358 | 3 | 1 | 2 |
| A03359 | 3 | 1 | 2 |
| A03360 | 3 | 3 | 0 |
| A03361 | 3 | 3 | 0 |
| A03362 | 3 | 3 | 0 |
| A03363 | 3 | 3 | 0 |
| A03364 | 3 | 1 | 2 |
| A03365 | 3 | 1 | 2 |
| A03366 | 3 | 1 | 2 |
| A03367 | 3 | 1 | 2 |
| A03368 | 3 | 1 | 2 |
| A03369 | 3 | 1 | 2 |
| A03370 | 3 | 1 | 2 |
| A03373 | 3 | 1 | 2 |
| A03375 | 3 | 2 | 1 |
| A03376 | 3 | 3 | 0 |
| A03377 | 3 | 1 | 2 |
| A03378 | 3 | 1 | 2 |
| A03379 | 3 | 3 | 0 |
| A03380 | 3 | 3 | 0 |
| A03381 | 3 | 3 | 0 |
| A03382 | 3 | 3 | 0 |
| A03383 | 3 | 1 | 2 |
| A03384 | 3 | 3 | 0 |
| A03385 | 3 | 3 | 0 |
| A03386 | 3 | 3 | 0 |
| A03387 | 3 | 1 | 2 |
| A03388 | 3 | 3 | 0 |
| A03389 | 3 | 1 | 2 |
| A03390 | 3 | 3 | 0 |
| A03391 | 3 | 3 | 0 |
| A03392 | 3 | 1 | 2 |
| A03394 | 3 | 1 | 2 |
| A03395 | 3 | 1 | 2 |
| A03396 | 3 | 3 | 0 |
| A03397 | 3 | 1 | 2 |
| A03398 | 3 | 3 | 0 |
| A03399 | 3 | 3 | 0 |
| A03400 | 3 | 3 | 0 |
| A03401 | 3 | 3 | 0 |
| A03403 | 3 | 1 | 2 |
| A03404 | 3 | 1 | 2 |
| A03405 | 3 | 1 | 2 |
| A03406 | 3 | 3 | 0 |
| A03407 | 3 | 3 | 0 |
| A03409 | 3 | 3 | 0 |
| A03410 | 3 | 1 | 2 |
| A03411 | 3 | 3 | 0 |
| A03412 | 3 | 1 | 2 |
| A03413 | 3 | 1 | 2 |
| A03416 | 3 | 1 | 2 |
| A03417 | 3 | 3 | 0 |
| A03418 | 3 | 3 | 0 |
| A03419 | 3 | 3 | 0 |
| A03420 | 3 | 3 | 0 |
| A03421 | 3 | 1 | 2 |
| A03422 | 3 | 1 | 2 |
| A03423 | 3 | 3 | 0 |
| A03424 | 3 | 3 | 0 |
| A03425 | 3 | 1 | 2 |
| A03426 | 3 | 1 | 2 |
| A03427 | 3 | 3 | 0 |
| A03428 | 3 | 3 | 0 |
| A03429 | 3 | 3 | 0 |
| A03430 | 3 | 1 | 2 |
| A03431 | 3 | 3 | 0 |
| A03432 | 3 | 1 | 2 |
| A03433 | 3 | 1 | 2 |
| A03434 | 3 | 1 | 2 |
| A03435 | 3 | 1 | 2 |
| A03436 | 3 | 1 | 2 |
| A03437 | 3 | 1 | 2 |
| A03438 | 3 | 1 | 2 |
| A03439 | 3 | 1 | 2 |
| A03440 | 3 | 1 | 2 |
| A03441 | 3 | 3 | 0 |
| A03442 | 3 | 3 | 0 |
| A03443 | 3 | 1 | 2 |
| A03444 | 3 | 1 | 2 |
| A03445 | 3 | 1 | 2 |
| A03446 | 3 | 1 | 2 |
| A03447 | 3 | 1 | 2 |
| A03448 | 3 | 3 | 0 |
| A03449 | 3 | 3 | 0 |
| A03450 | 3 | 1 | 2 |
| A03451 | 3 | 3 | 0 |
| A03452 | 3 | 1 | 2 |
| A03453 | 3 | 1 | 2 |
| A03454 | 3 | 1 | 2 |
| A03455 | 3 | 1 | 2 |
| A03456 | 3 | 1 | 2 |
| A03457 | 3 | 1 | 2 |
| A03458 | 3 | 2 | 1 |
| A03459 | 3 | 1 | 2 |
| A03460 | 3 | 1 | 2 |
| A03461 | 3 | 1 | 2 |
| A03462 | 3 | 1 | 2 |
| A03463 | 3 | 3 | 0 |
| A03464 | 3 | 3 | 0 |
| A03465 | 3 | 1 | 2 |
| A03466 | 3 | 3 | 0 |
| A03467 | 3 | 1 | 2 |
| A03468 | 3 | 1 | 2 |
| A03469 | 3 | 1 | 2 |
| A03470 | 3 | 1 | 2 |
| A03471 | 3 | 1 | 2 |
| A03473 | 3 | 3 | 0 |
| A03474 | 3 | 3 | 0 |
| A03475 | 3 | 3 | 0 |
| A03476 | 3 | 2 | 1 |
| A03477 | 3 | 1 | 2 |
| A03481 | 3 | 1 | 2 |
| A03482 | 3 | 3 | 0 |
| A03483 | 3 | 1 | 2 |
| A03484 | 3 | 1 | 2 |
| A03485 | 3 | 3 | 0 |
| A03486 | 3 | 1 | 2 |
| A03487 | 3 | 3 | 0 |
| A03488 | 3 | 3 | 0 |
| A03489 | 3 | 3 | 0 |
| A03490 | 3 | 3 | 0 |
| A03491 | 3 | 1 | 2 |
| A03492 | 3 | 3 | 0 |
| A03493 | 3 | 3 | 0 |
| A03494 | 3 | 3 | 0 |
| A03495 | 3 | 1 | 2 |
| A03496 | 3 | 1 | 2 |
| A03497 | 3 | 1 | 2 |
| A03500 | 3 | 3 | 0 |
| A03502 | 3 | 3 | 0 |
| A03503 | 3 | 3 | 0 |
| A03504 | 3 | 3 | 0 |
| A03505 | 3 | 2 | 1 |
| A03506 | 3 | 2 | 1 |
| A03507 | 3 | 2 | 1 |
| A03508 | 3 | 3 | 0 |
| A03509 | 3 | 1 | 2 |
| A03510 | 3 | 3 | 0 |
| A03511 | 3 | 2 | 1 |
| A03512 | 3 | 1 | 2 |
| A03513 | 3 | 1 | 2 |
| A03514 | 3 | 3 | 0 |
| A03515 | 3 | 1 | 2 |
| A03516 | 3 | 1 | 2 |
| A03517 | 3 | 3 | 0 |
| A03518 | 3 | 1 | 2 |
| A03519 | 3 | 3 | 0 |
| A03520 | 3 | 3 | 0 |
| A03521 | 3 | 1 | 2 |
| A03522 | 3 | 3 | 0 |
| A03523 | 3 | 3 | 0 |
| A03524 | 3 | 1 | 2 |
| A03525 | 3 | 1 | 2 |
| A03526 | 3 | 3 | 0 |
| A03527 | 3 | 1 | 2 |
| A03528 | 3 | 1 | 2 |
| A03529 | 3 | 1 | 2 |
| A03530 | 3 | 1 | 2 |
| A03531 | 3 | 1 | 2 |
| A03533 | 3 | 1 | 2 |
| A03534 | 3 | 3 | 0 |
| A03535 | 3 | 1 | 2 |
| A03536 | 3 | 3 | 0 |
| A03537 | 3 | 3 | 0 |
| A03538 | 3 | 1 | 2 |
| A03539 | 3 | 1 | 2 |
| A03540 | 3 | 3 | 0 |
| A03541 | 3 | 3 | 0 |
| A03542 | 3 | 3 | 0 |
| A03543 | 3 | 3 | 0 |
| A03544 | 3 | 3 | 0 |
| A03545 | 3 | 3 | 0 |
| A03546 | 3 | 2 | 1 |
| A03547 | 3 | 1 | 2 |
| A03548 | 3 | 3 | 0 |
| A03549 | 3 | 1 | 2 |
| A03550 | 3 | 1 | 2 |
| A03551 | 3 | 2 | 1 |
| A03552 | 3 | 1 | 2 |
| A03553 | 3 | 1 | 2 |
| A03554 | 3 | 1 | 2 |
| A03555 | 3 | 1 | 2 |
| A03556 | 3 | 1 | 2 |
| A03557 | 3 | 1 | 2 |
| A03558 | 3 | 3 | 0 |
| A03559 | 3 | 3 | 0 |
| A03560 | 3 | 1 | 2 |
| A03561 | 3 | 1 | 2 |
| A03563 | 3 | 1 | 2 |
| A03564 | 3 | 3 | 0 |
| A03565 | 3 | 1 | 2 |
| A03566 | 3 | 1 | 2 |
| A03567 | 3 | 1 | 2 |
| A03568 | 3 | 1 | 2 |
| A03569 | 3 | 1 | 2 |
| A03570 | 3 | 1 | 2 |
| A03571 | 3 | 1 | 2 |
| A03572 | 3 | 1 | 2 |
| A03573 | 3 | 1 | 2 |
| A03574 | 3 | 3 | 0 |
| A03575 | 3 | 1 | 2 |
| A03576 | 3 | 1 | 2 |
| A03577 | 3 | 3 | 0 |
| A03578 | 3 | 3 | 0 |
| A03579 | 3 | 1 | 2 |
| A03580 | 3 | 1 | 2 |
| A03581 | 3 | 1 | 2 |
| A03582 | 3 | 1 | 2 |
| A03583 | 3 | 1 | 2 |
| A03584 | 3 | 1 | 2 |
| A03585 | 3 | 3 | 0 |
| A03586 | 3 | 3 | 0 |
| A03587 | 3 | 1 | 2 |
| A03588 | 3 | 1 | 2 |
| A03589 | 3 | 1 | 2 |
| A03590 | 3 | 1 | 2 |
| A03591 | 3 | 3 | 0 |
| A03592 | 3 | 3 | 0 |
| A03593 | 3 | 3 | 0 |
| A03594 | 3 | 1 | 2 |
| A03595 | 3 | 1 | 2 |
| A03596 | 3 | 1 | 2 |
| A03597 | 3 | 1 | 2 |
| A03598 | 3 | 3 | 0 |
| A03599 | 3 | 1 | 2 |
| A03600 | 3 | 1 | 2 |
| A03601 | 3 | 1 | 2 |
| A03602 | 3 | 3 | 0 |
| A03603 | 3 | 1 | 2 |
| A03604 | 3 | 1 | 2 |
| A03605 | 3 | 1 | 2 |
| A03606 | 3 | 1 | 2 |
| A03607 | 3 | 1 | 2 |
| A03608 | 3 | 1 | 2 |
| A03609 | 3 | 3 | 0 |
| A03610 | 3 | 3 | 0 |
| A03611 | 3 | 2 | 1 |
| A03612 | 3 | 2 | 1 |
| A03613 | 3 | 1 | 2 |
| A03614 | 3 | 1 | 2 |
| A03615 | 3 | 3 | 0 |
| A03616 | 3 | 1 | 2 |
| A03618 | 3 | 3 | 0 |
| A03619 | 3 | 3 | 0 |
| A03620 | 3 | 3 | 0 |
| A03621 | 3 | 1 | 2 |
| A03623 | 3 | 3 | 0 |
| A03624 | 3 | 3 | 0 |
| A03625 | 3 | 1 | 2 |
| A03626 | 3 | 2 | 1 |
| A03627 | 3 | 3 | 0 |
| A03628 | 3 | 1 | 2 |
| A03629 | 3 | 1 | 2 |
| A03630 | 3 | 1 | 2 |
| A03631 | 3 | 3 | 0 |
| A03632 | 3 | 3 | 0 |
| A03633 | 3 | 1 | 2 |
| A03634 | 3 | 3 | 0 |
| A03635 | 3 | 3 | 0 |
| A03636 | 3 | 1 | 2 |
| A03637 | 3 | 1 | 2 |
| A03638 | 3 | 1 | 2 |
| A03639 | 3 | 1 | 2 |
| A03640 | 3 | 3 | 0 |
| A03641 | 3 | 2 | 1 |
| A03642 | 3 | 3 | 0 |
| A03643 | 3 | 3 | 0 |
| A03644 | 3 | 3 | 0 |
| A03645 | 3 | 1 | 2 |
| A03646 | 3 | 3 | 0 |
| A03647 | 3 | 3 | 0 |
| A03648 | 3 | 3 | 0 |
| A03649 | 3 | 1 | 2 |
| A03650 | 3 | 1 | 2 |
| A03651 | 3 | 3 | 0 |
| A03652 | 3 | 3 | 0 |
| A03656 | 3 | 3 | 0 |
| A03657 | 3 | 3 | 0 |
| A03660 | 3 | 3 | 0 |
| A03661 | 3 | 3 | 0 |
| A03662 | 3 | 3 | 0 |
| A03674 | 3 | 1 | 2 |
| A03675 | 3 | 1 | 2 |
| A03676 | 3 | 3 | 0 |
| A03679 | 3 | 1 | 2 |
| A03680 | 3 | 1 | 2 |
| A03681 | 3 | 3 | 0 |
| A03684 | 3 | 1 | 2 |
| A03685 | 3 | 2 | 1 |
| A03686 | 3 | 1 | 2 |
| A03687 | 3 | 1 | 2 |
| A03690 | 3 | 3 | 0 |
| A03691 | 3 | 3 | 0 |
| A03694 | 3 | 3 | 0 |
| A03698 | 3 | 1 | 2 |
| A03700 | 3 | 3 | 0 |
| A03701 | 3 | 3 | 0 |
| A03704 | 3 | 3 | 0 |
| A03705 | 3 | 3 | 0 |
| A03706 | 3 | 3 | 0 |
| A03707 | 3 | 3 | 0 |
| A03709 | 3 | 2 | 1 |
| A03711 | 3 | 2 | 1 |
| A03712 | 3 | 1 | 2 |
| A03713 | 3 | 1 | 2 |
| A03714 | 3 | 1 | 2 |
| A03715 | 3 | 1 | 2 |
| A03716 | 3 | 3 | 0 |
| A03717 | 3 | 1 | 2 |
| A03718 | 3 | 1 | 2 |
| A03719 | 3 | 1 | 2 |
| A03720 | 3 | 1 | 2 |
| A03721 | 3 | 1 | 2 |
| A03722 | 3 | 1 | 2 |
| A03723 | 3 | 1 | 2 |
| A03724 | 3 | 1 | 2 |
| A03725 | 3 | 1 | 2 |
| A03726 | 3 | 1 | 2 |
| A03727 | 3 | 1 | 2 |
| A03728 | 3 | 1 | 2 |
| A03729 | 3 | 3 | 0 |
| A03730 | 3 | 1 | 2 |
| A03731 | 3 | 3 | 0 |
| A03732 | 3 | 2 | 1 |
| A03733 | 3 | 1 | 2 |
| A03734 | 3 | 3 | 0 |
| A03735 | 3 | 3 | 0 |
| A03736 | 3 | 3 | 0 |
| A03737 | 3 | 1 | 2 |
| A03738 | 3 | 1 | 2 |
| A03739 | 3 | 1 | 2 |
| A03740 | 3 | 1 | 2 |
| A03741 | 3 | 1 | 2 |
| A03742 | 3 | 1 | 2 |
| A03744 | 3 | 1 | 2 |
| A03745 | 3 | 1 | 2 |
| A03746 | 3 | 1 | 2 |
| A03747 | 3 | 1 | 2 |
| A03748 | 3 | 1 | 2 |
| A03749 | 3 | 1 | 2 |
| A03750 | 3 | 1 | 2 |
| A03751 | 3 | 1 | 2 |
| A03753 | 3 | 1 | 2 |
| A03778 | 3 | 1 | 2 |
| A03779 | 3 | 1 | 2 |
| A03780 | 3 | 1 | 2 |
| A03781 | 3 | 1 | 2 |
| A03782 | 3 | 1 | 2 |
| A03784 | 3 | 1 | 2 |
| A03785 | 3 | 1 | 2 |
| A03786 | 3 | 1 | 2 |
| A03787 | 3 | 1 | 2 |
| A03788 | 3 | 1 | 2 |
| A03790 | 3 | 1 | 2 |
| A03792 | 3 | 1 | 2 |
| A03793 | 3 | 1 | 2 |
| A03794 | 3 | 1 | 2 |
| A03795 | 3 | 1 | 2 |
| A03796 | 3 | 1 | 2 |
| A03797 | 3 | 1 | 2 |
| A03798 | 3 | 1 | 2 |
| A03799 | 3 | 1 | 2 |
| A03800 | 3 | 1 | 2 |
| A03801 | 3 | 1 | 2 |
| A03803 | 3 | 1 | 2 |
| A03805 | 3 | 1 | 2 |
| A03806 | 3 | 1 | 2 |
| A03807 | 3 | 1 | 2 |
| A03808 | 3 | 1 | 2 |
| A03809 | 3 | 1 | 2 |
| A03810 | 3 | 1 | 2 |
| A03811 | 3 | 1 | 2 |
| A03813 | 3 | 1 | 2 |
| A03814 | 3 | 1 | 2 |
| A03815 | 3 | 1 | 2 |
| A03816 | 3 | 1 | 2 |
| A03817 | 3 | 1 | 2 |
| A03818 | 3 | 1 | 2 |
| A03819 | 3 | 1 | 2 |
| A03820 | 3 | 1 | 2 |
| A03821 | 3 | 1 | 2 |
| A03823 | 3 | 1 | 2 |
| A03825 | 3 | 1 | 2 |
| A03826 | 3 | 1 | 2 |
| A03827 | 3 | 1 | 2 |
| A03828 | 3 | 1 | 2 |
| A03829 | 3 | 1 | 2 |
| A03830 | 3 | 1 | 2 |
| A03831 | 3 | 1 | 2 |
| A03833 | 3 | 1 | 2 |
| A03834 | 3 | 1 | 2 |
| A03835 | 3 | 1 | 2 |
| A03836 | 3 | 1 | 2 |
| A03837 | 3 | 1 | 2 |
| A03838 | 3 | 1 | 2 |
| A03839 | 3 | 1 | 2 |
| A03840 | 3 | 1 | 2 |
| A03841 | 3 | 1 | 2 |
| A03842 | 3 | 1 | 2 |
| A03843 | 3 | 1 | 2 |
| A03845 | 3 | 1 | 2 |
| A03847 | 3 | 1 | 2 |
| A03848 | 3 | 1 | 2 |
| A03849 | 3 | 1 | 2 |
| A03850 | 3 | 1 | 2 |
| A03852 | 3 | 1 | 2 |
| A03853 | 3 | 1 | 2 |
| A03854 | 3 | 1 | 2 |
| A03855 | 3 | 1 | 2 |
| A03856 | 3 | 1 | 2 |
| A03857 | 3 | 1 | 2 |
| A03858 | 3 | 1 | 2 |
| A03859 | 3 | 1 | 2 |
| A03860 | 3 | 1 | 2 |
| A03863 | 3 | 1 | 2 |
| A03865 | 3 | 1 | 2 |
| A03867 | 3 | 1 | 2 |
| A03868 | 3 | 1 | 2 |
| A03869 | 3 | 1 | 2 |
| A03870 | 3 | 1 | 2 |
| A03872 | 3 | 1 | 2 |
| A03873 | 3 | 1 | 2 |
| A03876 | 3 | 1 | 2 |
| A03877 | 3 | 1 | 2 |
| A03878 | 3 | 1 | 2 |
| A03879 | 3 | 1 | 2 |
| A03881 | 3 | 1 | 2 |
| A03882 | 3 | 1 | 2 |
| A03883 | 3 | 1 | 2 |
| A03884 | 3 | 1 | 2 |
| A03885 | 3 | 1 | 2 |
| A03886 | 3 | 1 | 2 |
| A03888 | 3 | 1 | 2 |
| A03889 | 3 | 1 | 2 |
| A03890 | 3 | 1 | 2 |
| A03891 | 3 | 1 | 2 |
| A03892 | 3 | 1 | 2 |
| A03895 | 3 | 1 | 2 |
| A03896 | 3 | 1 | 2 |
| A03897 | 3 | 1 | 2 |
| A03898 | 3 | 1 | 2 |
| A03899 | 3 | 1 | 2 |
| A03900 | 3 | 1 | 2 |
| A03901 | 3 | 1 | 2 |
| A03902 | 3 | 1 | 2 |
| A03904 | 3 | 1 | 2 |
| A03905 | 3 | 1 | 2 |
| A03906 | 3 | 1 | 2 |
| A03907 | 3 | 1 | 2 |
| A03908 | 3 | 1 | 2 |
| A03909 | 3 | 1 | 2 |
| A03910 | 3 | 1 | 2 |
| A03911 | 3 | 1 | 2 |
| A03915 | 3 | 1 | 2 |
| A03916 | 3 | 1 | 2 |
| A03918 | 3 | 1 | 2 |
| A03919 | 3 | 1 | 2 |
| A03920 | 3 | 1 | 2 |
| A03922 | 3 | 1 | 2 |
| A03923 | 3 | 1 | 2 |
| A03924 | 3 | 1 | 2 |
| A03925 | 3 | 1 | 2 |
| A03926 | 3 | 1 | 2 |
| A03927 | 3 | 1 | 2 |
| A03928 | 3 | 1 | 2 |
| A03929 | 3 | 1 | 2 |
| A03931 | 3 | 1 | 2 |
| A03932 | 3 | 1 | 2 |
| A03933 | 3 | 1 | 2 |
| A03934 | 3 | 1 | 2 |
| A03935 | 3 | 1 | 2 |
| A03936 | 3 | 1 | 2 |
| A03937 | 3 | 1 | 2 |
| A03938 | 3 | 1 | 2 |
| A03939 | 3 | 1 | 2 |
| A03940 | 3 | 1 | 2 |
| A03941 | 3 | 1 | 2 |
| A03942 | 3 | 1 | 2 |
| A03943 | 3 | 1 | 2 |
| A03944 | 3 | 1 | 2 |
| A03945 | 3 | 1 | 2 |
| A03946 | 3 | 1 | 2 |
| A03948 | 3 | 1 | 2 |
| A03949 | 3 | 1 | 2 |
| A03950 | 3 | 1 | 2 |
| A03951 | 3 | 1 | 2 |
| A03952 | 3 | 1 | 2 |
| A03953 | 3 | 1 | 2 |
| A03954 | 3 | 1 | 2 |
| A03955 | 3 | 1 | 2 |
| A03956 | 3 | 1 | 2 |
| A03957 | 3 | 1 | 2 |
| A03958 | 3 | 1 | 2 |
| A03959 | 3 | 1 | 2 |
| A03960 | 3 | 1 | 2 |
| A03961 | 3 | 1 | 2 |
| A03962 | 3 | 1 | 2 |
| A03963 | 3 | 1 | 2 |
| A03965 | 3 | 1 | 2 |
| A03967 | 3 | 1 | 2 |
| A03968 | 3 | 1 | 2 |
| A03969 | 3 | 1 | 2 |
| A03971 | 3 | 1 | 2 |
| A03972 | 3 | 1 | 2 |
| A03973 | 3 | 1 | 2 |
| A03975 | 3 | 1 | 2 |
| A03976 | 3 | 1 | 2 |
| A03977 | 3 | 1 | 2 |
| A03978 | 3 | 1 | 2 |
| A03979 | 3 | 1 | 2 |
| A03980 | 3 | 1 | 2 |
| A03981 | 3 | 1 | 2 |
| A03983 | 3 | 1 | 2 |
| A03984 | 3 | 1 | 2 |
| A03985 | 3 | 1 | 2 |
| A03986 | 3 | 1 | 2 |
| A03987 | 3 | 1 | 2 |
| A03988 | 3 | 1 | 2 |
| A03989 | 3 | 1 | 2 |
| A03991 | 3 | 1 | 2 |
| A03992 | 3 | 1 | 2 |
| A03993 | 3 | 1 | 2 |
| A03994 | 3 | 1 | 2 |
| A03995 | 3 | 1 | 2 |
| A03996 | 3 | 1 | 2 |
| A03997 | 3 | 1 | 2 |
| A03998 | 3 | 1 | 2 |
| A03999 | 3 | 1 | 2 |
| A04000 | 3 | 1 | 2 |
| A04002 | 3 | 1 | 2 |
| A04004 | 3 | 1 | 2 |
| A04005 | 3 | 1 | 2 |
| A04006 | 3 | 1 | 2 |
| A04007 | 3 | 1 | 2 |
| A04008 | 3 | 1 | 2 |
| A04009 | 3 | 1 | 2 |
| A04010 | 3 | 1 | 2 |
| A04011 | 3 | 1 | 2 |
| A04012 | 3 | 1 | 2 |
| A04013 | 3 | 1 | 2 |
| A04014 | 3 | 1 | 2 |
| A04015 | 3 | 1 | 2 |
| A04016 | 3 | 1 | 2 |
| A04017 | 3 | 1 | 2 |
| A04018 | 3 | 1 | 2 |
| A04019 | 3 | 1 | 2 |
| A04020 | 3 | 1 | 2 |
| A04021 | 3 | 1 | 2 |
| A04022 | 3 | 1 | 2 |
| A04023 | 3 | 1 | 2 |
| A04024 | 3 | 1 | 2 |
| A04025 | 3 | 1 | 2 |
| A04026 | 3 | 1 | 2 |
| A04027 | 3 | 1 | 2 |
| A04028 | 3 | 1 | 2 |
| A04029 | 3 | 1 | 2 |
| A04030 | 3 | 1 | 2 |
| A04031 | 3 | 1 | 2 |
| A04032 | 3 | 1 | 2 |
| A04033 | 3 | 1 | 2 |
| A04034 | 3 | 1 | 2 |
| A04035 | 3 | 1 | 2 |
| A04037 | 3 | 1 | 2 |
| A04039 | 3 | 1 | 2 |
| A04040 | 3 | 1 | 2 |
| A04041 | 3 | 1 | 2 |
| A04042 | 3 | 1 | 2 |
| A04043 | 3 | 1 | 2 |
| A04044 | 3 | 1 | 2 |
| A04045 | 3 | 1 | 2 |
| A04046 | 3 | 1 | 2 |
| A04047 | 3 | 1 | 2 |
| A04048 | 3 | 1 | 2 |
| A04049 | 3 | 1 | 2 |
| A04050 | 3 | 1 | 2 |
| A04051 | 3 | 1 | 2 |
| A04052 | 3 | 1 | 2 |
| A04053 | 3 | 1 | 2 |
| A04054 | 3 | 1 | 2 |
| A04055 | 3 | 1 | 2 |
| A04056 | 3 | 1 | 2 |
| A04057 | 3 | 1 | 2 |
| A04058 | 3 | 1 | 2 |
| A04059 | 3 | 1 | 2 |
| A04060 | 3 | 1 | 2 |
| A04062 | 3 | 1 | 2 |
| A04063 | 3 | 1 | 2 |
| A04064 | 3 | 1 | 2 |
| A04065 | 3 | 1 | 2 |
| A04066 | 3 | 1 | 2 |
| A04067 | 3 | 1 | 2 |
| A04068 | 3 | 1 | 2 |
| A04069 | 3 | 1 | 2 |
| A04070 | 3 | 1 | 2 |
| A04071 | 3 | 1 | 2 |
| A04072 | 3 | 1 | 2 |
| A04073 | 3 | 1 | 2 |
| A04074 | 3 | 1 | 2 |
| A04075 | 3 | 1 | 2 |
| A04076 | 3 | 1 | 2 |
| A04077 | 3 | 1 | 2 |
| A04078 | 3 | 1 | 2 |
| A04079 | 3 | 1 | 2 |
| A04080 | 3 | 1 | 2 |
| A04081 | 3 | 1 | 2 |
| A04082 | 3 | 1 | 2 |
| A04083 | 3 | 1 | 2 |
| A04084 | 3 | 1 | 2 |
| A04085 | 3 | 1 | 2 |
| A04086 | 3 | 1 | 2 |
| A04087 | 3 | 1 | 2 |
| A04089 | 3 | 1 | 2 |
| A04090 | 3 | 1 | 2 |
| A04091 | 3 | 1 | 2 |
| A04092 | 3 | 1 | 2 |
| A04093 | 3 | 1 | 2 |
| A04094 | 3 | 1 | 2 |
| A04095 | 3 | 1 | 2 |
| A04096 | 3 | 1 | 2 |
| A04097 | 3 | 1 | 2 |
| A04098 | 3 | 1 | 2 |
| A04100 | 3 | 1 | 2 |
| A04101 | 3 | 1 | 2 |
| A04102 | 3 | 1 | 2 |
| A04103 | 3 | 1 | 2 |
| A04104 | 3 | 1 | 2 |
| A04106 | 3 | 1 | 2 |
| A04107 | 3 | 1 | 2 |
| A04109 | 3 | 1 | 2 |
| A04110 | 3 | 1 | 2 |
| A04111 | 3 | 1 | 2 |
| A04112 | 3 | 1 | 2 |
| A04113 | 3 | 1 | 2 |
| A04115 | 3 | 1 | 2 |
| A04117 | 3 | 1 | 2 |
| A04118 | 3 | 1 | 2 |
| A04119 | 3 | 1 | 2 |
| A04120 | 3 | 1 | 2 |
| A04121 | 3 | 1 | 2 |
| A04123 | 3 | 1 | 2 |
| A04125 | 3 | 1 | 2 |
| A04126 | 3 | 1 | 2 |
| A04128 | 3 | 1 | 2 |
| A04131 | 3 | 1 | 2 |
| A04132 | 3 | 1 | 2 |
| A04133 | 3 | 1 | 2 |
| A04134 | 3 | 1 | 2 |
| A04135 | 3 | 1 | 2 |
| A04136 | 3 | 1 | 2 |
| A04137 | 3 | 1 | 2 |
| A04138 | 3 | 1 | 2 |
| A04139 | 3 | 1 | 2 |
| A04144 | 3 | 1 | 2 |
| A04149 | 3 | 1 | 2 |
| A04150 | 3 | 1 | 2 |
| A04151 | 3 | 1 | 2 |
| A04152 | 3 | 1 | 2 |
| AJ0001 | 3 | 3 | 0 |
| AJ0002 | 3 | 3 | 0 |
| AJ0003 | 3 | 3 | 0 |
| AJ0004 | 3 | 3 | 0 |
| AJ0005 | 3 | 3 | 0 |
| AJ0006 | 3 | 3 | 0 |
| AJ0007 | 3 | 3 | 0 |
| AJ0008 | 3 | 3 | 0 |
| AJ0009 | 3 | 3 | 0 |
| AJ0010 | 3 | 3 | 0 |
| AJ0011 | 3 | 3 | 0 |
| AJ0012 | 3 | 3 | 0 |
| AJ0013 | 3 | 3 | 0 |
| AJ0014 | 3 | 3 | 0 |
| AJ0015 | 3 | 3 | 0 |
| AJ0016 | 3 | 3 | 0 |
| AJ0017 | 3 | 3 | 0 |
| AJ0018 | 3 | 3 | 0 |
| AJ0019 | 3 | 3 | 0 |
| AJ0020 | 3 | 3 | 0 |
| AJ0021 | 3 | 3 | 0 |
| AJ0022 | 3 | 3 | 0 |
| AJ0023 | 3 | 3 | 0 |
| AJ0024 | 3 | 3 | 0 |
| AJ0025 | 3 | 3 | 0 |
| AJ0026 | 3 | 3 | 0 |
| AJ0027 | 3 | 3 | 0 |
| AJ0028 | 3 | 3 | 0 |
| AJ0029 | 3 | 3 | 0 |
| AJ0030 | 3 | 3 | 0 |
| AJ0031 | 3 | 3 | 0 |
| AJ0032 | 3 | 3 | 0 |
| AJ0033 | 3 | 3 | 0 |
| AJ0034 | 3 | 3 | 0 |
| AJ0035 | 3 | 3 | 0 |
| AJ0036 | 3 | 3 | 0 |
| AJ0037 | 3 | 3 | 0 |
| AJ0038 | 3 | 3 | 0 |
| AJ0039 | 3 | 3 | 0 |
| AJ0040 | 3 | 3 | 0 |
| AJ0041 | 3 | 3 | 0 |
| AJ0042 | 3 | 3 | 0 |
| AJ0043 | 3 | 3 | 0 |
| AJ0044 | 3 | 3 | 0 |
| AJ0045 | 3 | 3 | 0 |
| AJ0046 | 3 | 3 | 0 |
| AJ0047 | 3 | 3 | 0 |
| AJ0048 | 3 | 3 | 0 |
| AJ0049 | 3 | 3 | 0 |
| AJ0050 | 3 | 3 | 0 |
| AJ0051 | 3 | 3 | 0 |
| AJ0052 | 3 | 3 | 0 |
| AJ0053 | 3 | 3 | 0 |
| AJ0054 | 3 | 3 | 0 |
| AJ0055 | 3 | 3 | 0 |
| AJ0056 | 3 | 3 | 0 |
| AJ0057 | 3 | 3 | 0 |
| AJ0058 | 3 | 3 | 0 |
| AJ0059 | 3 | 3 | 0 |
| AJ0060 | 3 | 3 | 0 |
| AJ0061 | 3 | 3 | 0 |
| AJ0062 | 3 | 3 | 0 |
| AJ0063 | 3 | 3 | 0 |
| AJ0064 | 3 | 3 | 0 |
| AJ0065 | 3 | 3 | 0 |
| AJ0066 | 3 | 2 | 1 |
| AJ0067 | 3 | 2 | 1 |
| AJ0068 | 3 | 2 | 1 |
| AJ0069 | 3 | 3 | 0 |
| AJ0070 | 3 | 3 | 0 |
| AJ0071 | 3 | 3 | 0 |
| AJ0072 | 3 | 3 | 0 |
| AJ0073 | 3 | 2 | 1 |
| AJ0074 | 3 | 2 | 1 |
| AJ0075 | 3 | 2 | 1 |
| AJ0076 | 3 | 2 | 1 |
| AJ0077 | 3 | 3 | 0 |
| AJ0078 | 3 | 2 | 1 |
| AJ0079 | 3 | 3 | 0 |
| AJ0080 | 3 | 3 | 0 |
| AJ0081 | 3 | 3 | 0 |
| AJ0082 | 3 | 3 | 0 |
| AJ0083 | 3 | 3 | 0 |
| AJ0084 | 3 | 3 | 0 |
| AJ0085 | 3 | 3 | 0 |
| AJ0086 | 3 | 3 | 0 |
| AJ0087 | 3 | 3 | 0 |
| AJ0088 | 3 | 3 | 0 |
| AJ0090 | 3 | 3 | 0 |
| AJ0091 | 3 | 3 | 0 |
| AJ0092 | 3 | 3 | 0 |
| AJ0093 | 3 | 3 | 0 |
| AJ0094 | 3 | 3 | 0 |
| AJ0095 | 3 | 3 | 0 |
| AJ0096 | 3 | 2 | 1 |
| BM071 | 3 | 3 | 0 |
| BM072 | 3 | 3 | 0 |
| BM073 | 3 | 3 | 0 |
| BM074 | 3 | 3 | 0 |
| BM076 | 3 | 3 | 0 |
| BM077 | 3 | 3 | 0 |
| BM078 | 3 | 3 | 0 |
| BM079 | 3 | 3 | 0 |
| BM081 | 3 | 3 | 0 |
| BM082 | 3 | 3 | 0 |
| BM083 | 3 | 3 | 0 |
| BM085 | 3 | 2 | 1 |
| BM100 | 3 | 3 | 0 |
| BM101 | 3 | 3 | 0 |
| BM102 | 3 | 3 | 0 |
| BM103 | 3 | 3 | 0 |
| BM104 | 3 | 3 | 0 |
| BM105 | 3 | 3 | 0 |
| BM108 | 3 | 3 | 0 |
| BM109 | 3 | 3 | 0 |
| BM110 | 3 | 3 | 0 |
| BM111 | 3 | 3 | 0 |
| BM113 | 3 | 3 | 0 |
| BM114 | 3 | 3 | 0 |
| BM115 | 3 | 3 | 0 |
| BM116 | 3 | 3 | 0 |
| BM117 | 3 | 3 | 0 |
| BM118 | 3 | 3 | 0 |
| BM119 | 3 | 3 | 0 |
| BM120 | 3 | 3 | 0 |
| BM121 | 3 | 3 | 0 |
| BM122 | 3 | 3 | 0 |
| BM123 | 3 | 3 | 0 |
| BM124 | 3 | 3 | 0 |
| BM126 | 3 | 3 | 0 |
| BM127 | 3 | 3 | 0 |
| BM133 | 3 | 3 | 0 |
| BM134 | 3 | 3 | 0 |
| BM135 | 3 | 3 | 0 |
| BM136 | 3 | 3 | 0 |
| BM138 | 3 | 3 | 0 |
| BM139 | 3 | 3 | 0 |
| BM140 | 3 | 3 | 0 |
| BM141 | 3 | 3 | 0 |
| BM145 | 3 | 3 | 0 |
| BM146 | 3 | 3 | 0 |
| BM147 | 3 | 3 | 0 |
| BM148 | 3 | 3 | 0 |
| BM149 | 3 | 3 | 0 |
| BM150 | 3 | 3 | 0 |
| BM151 | 3 | 3 | 0 |
| BM152 | 3 | 3 | 0 |
| BM153 | 3 | 3 | 0 |
| BM154 | 3 | 3 | 0 |
| BM156 | 3 | 3 | 0 |
| BM157 | 3 | 3 | 0 |
| BM158 | 3 | 3 | 0 |
| BM159 | 3 | 3 | 0 |
| BM160 | 3 | 3 | 0 |
| BM162 | 3 | 3 | 0 |
| BM164 | 3 | 3 | 0 |
| BM165 | 3 | 3 | 0 |
| BM166 | 3 | 3 | 0 |
| BM167 | 3 | 3 | 0 |
| BM168 | 3 | 3 | 0 |
| BM169 | 3 | 3 | 0 |
| BM174 | 3 | 3 | 0 |
| BM175 | 3 | 3 | 0 |
| BM177 | 3 | 3 | 0 |
| BM178 | 3 | 3 | 0 |
| BM179 | 3 | 3 | 0 |
| BM180 | 3 | 3 | 0 |
| BM183 | 3 | 3 | 0 |
| BM185 | 3 | 3 | 0 |
| BM186 | 3 | 3 | 0 |
| BM187 | 3 | 3 | 0 |
| BM188 | 3 | 3 | 0 |
| BM189 | 3 | 3 | 0 |
| BM190 | 3 | 3 | 0 |
| BM191 | 3 | 3 | 0 |
| BM192 | 3 | 3 | 0 |
| DK0002 | 3 | 3 | 0 |
| DK0004 | 3 | 1 | 2 |
| DK0005 | 3 | 1 | 2 |
| DK0006 | 3 | 3 | 0 |
| DK0007 | 3 | 3 | 0 |
| DK0008 | 3 | 1 | 2 |
| DK0009 | 3 | 1 | 2 |
| DK0010 | 3 | 3 | 0 |
| DK0011 | 3 | 1 | 2 |
| DK0012 | 3 | 1 | 2 |
| DK0014 | 3 | 1 | 2 |
| DK0016 | 3 | 1 | 2 |
| DK0017 | 3 | 1 | 2 |
| DK0018 | 3 | 1 | 2 |
| DK0019 | 3 | 3 | 0 |
| DK0022 | 3 | 3 | 0 |
| DK0023 | 3 | 3 | 0 |
| DK0025 | 3 | 1 | 2 |
| DK0026 | 3 | 1 | 2 |
| DK0027 | 3 | 2 | 1 |
| DK0028 | 3 | 1 | 2 |
| DK0029 | 3 | 1 | 2 |
| DK0030 | 3 | 1 | 2 |
| DK0031 | 3 | 1 | 2 |
| DK0032 | 3 | 1 | 2 |
| DK0033 | 3 | 3 | 0 |
| DK0034 | 3 | 1 | 2 |
| DK0035 | 3 | 1 | 2 |
| DK0038 | 3 | 1 | 2 |
| DK0039 | 3 | 3 | 0 |
| DK0040 | 3 | 1 | 2 |
| DK0041 | 3 | 3 | 0 |
| DK0042 | 3 | 1 | 2 |
| DK0043 | 3 | 3 | 0 |
| DK0044 | 3 | 3 | 0 |
| DK0045 | 3 | 3 | 0 |
| DK0046 | 3 | 2 | 1 |
| DK0047 | 3 | 1 | 2 |
| DK0048 | 3 | 1 | 2 |
| DK0049 | 3 | 1 | 2 |
| DK0050 | 3 | 1 | 2 |
| DK0051 | 3 | 1 | 2 |
| DK0052 | 3 | 1 | 2 |
| DK0053 | 3 | 1 | 2 |
| DK0055 | 3 | 1 | 2 |
| DK0056 | 3 | 1 | 2 |
| DK0058 | 3 | 1 | 2 |
| DK0060 | 3 | 1 | 2 |
| DK0061 | 3 | 3 | 0 |
| DK0062 | 3 | 1 | 2 |
| DK0065 | 3 | 3 | 0 |
| DK0066 | 3 | 1 | 2 |
| DK0067 | 3 | 3 | 0 |
| DK0068 | 3 | 1 | 2 |
| DK0069 | 3 | 1 | 2 |
| DK0070 | 3 | 3 | 0 |
| DK0071 | 3 | 1 | 2 |
| DK0072 | 3 | 1 | 2 |
| DK0075 | 3 | 1 | 2 |
| DK0076 | 3 | 3 | 0 |
| DK0077 | 3 | 1 | 2 |
| DK0078 | 3 | 1 | 2 |
| DK0079 | 3 | 1 | 2 |
| DK0081 | 3 | 1 | 2 |
| DK0082 | 3 | 1 | 2 |
| DK0084 | 3 | 3 | 0 |
| DK0085 | 3 | 1 | 2 |
| DK0086 | 3 | 3 | 0 |
| DK0087 | 3 | 1 | 2 |
| DK0088 | 3 | 1 | 2 |
| DK0090 | 3 | 1 | 2 |
| DK0091 | 3 | 1 | 2 |
| DK0092 | 3 | 1 | 2 |
| DK0093 | 3 | 3 | 0 |
| DK0097 | 3 | 1 | 2 |
| DK0098 | 3 | 3 | 0 |
| DK0099 | 3 | 1 | 2 |
| DK0100 | 3 | 1 | 2 |
| DK0101 | 3 | 3 | 0 |
| DK0102 | 3 | 1 | 2 |
| DK0103 | 3 | 1 | 2 |
| DK0104 | 3 | 1 | 2 |
| DK0105 | 3 | 1 | 2 |
| DK0106 | 3 | 1 | 2 |
| DK0107 | 3 | 3 | 0 |
| DK0108 | 3 | 1 | 2 |
| DK0109 | 3 | 1 | 2 |
| DK0110 | 3 | 3 | 0 |
| DK0111 | 3 | 1 | 2 |
| DK0112 | 3 | 3 | 0 |
| DK0113 | 3 | 1 | 2 |
| DK0114 | 3 | 1 | 2 |
| DK0115 | 3 | 1 | 2 |
| DK0116 | 3 | 3 | 0 |
| DK0117 | 3 | 3 | 0 |
| DK0118 | 3 | 1 | 2 |
| DK0119 | 3 | 3 | 0 |
| DK0120 | 3 | 3 | 0 |
| DK0121 | 3 | 3 | 0 |
| DK0122 | 3 | 1 | 2 |
| DK0123 | 3 | 1 | 2 |
| DK0124 | 3 | 1 | 2 |
| DK0125 | 3 | 3 | 0 |
| DK0126 | 3 | 1 | 2 |
| DK0127 | 3 | 1 | 2 |
| DK0128 | 3 | 3 | 0 |
| DK0129 | 3 | 1 | 2 |
| DK0130 | 3 | 1 | 2 |
| DK0131 | 3 | 1 | 2 |
| DK0132 | 3 | 1 | 2 |
| DK0133 | 3 | 3 | 0 |
| DK0134 | 3 | 1 | 2 |
| DK0135 | 3 | 1 | 2 |
| DK0136 | 3 | 1 | 2 |
| DK0137 | 3 | 1 | 2 |
| DK0138 | 3 | 1 | 2 |
| DK0139 | 3 | 3 | 0 |
| DK0140 | 3 | 1 | 2 |
| DK0141 | 3 | 1 | 2 |
| DK0142 | 3 | 1 | 2 |
| DK0144 | 3 | 1 | 2 |
| DK0145 | 3 | 3 | 0 |
| DK0146 | 3 | 1 | 2 |
| DK0147 | 3 | 1 | 2 |
| DK0148 | 3 | 1 | 2 |
| DK0149 | 3 | 3 | 0 |
| DK0150 | 3 | 1 | 2 |
| DK0152 | 3 | 1 | 2 |
| DK0153 | 3 | 1 | 2 |
| DK0154 | 3 | 1 | 2 |
| DK0155 | 3 | 3 | 0 |
| DK0156 | 3 | 1 | 2 |
| DK0157 | 3 | 1 | 2 |
| DK0158 | 3 | 1 | 2 |
| DK0159 | 3 | 1 | 2 |
| DK0160 | 3 | 1 | 2 |
| DK0161 | 3 | 3 | 0 |
| DK0162 | 3 | 3 | 0 |
| DK0163 | 3 | 1 | 2 |
| DK0164 | 3 | 1 | 2 |
| DK0165 | 3 | 1 | 2 |
| DK0166 | 3 | 1 | 2 |
| DK0167 | 3 | 1 | 2 |
| DK0168 | 3 | 1 | 2 |
| DK0169 | 3 | 3 | 0 |
| DK0170 | 3 | 1 | 2 |
| DK0171 | 3 | 1 | 2 |
| DK0172 | 3 | 3 | 0 |
| DK0173 | 3 | 1 | 2 |
| DK0174 | 3 | 1 | 2 |
| DK0176 | 3 | 3 | 0 |
| DK0177 | 3 | 1 | 2 |
| DK0178 | 3 | 1 | 2 |
| DK0179 | 3 | 1 | 2 |
| DK0180 | 3 | 1 | 2 |
| DK0181 | 3 | 1 | 2 |
| DK0182 | 3 | 1 | 2 |
| DK0183 | 3 | 1 | 2 |
| DK0184 | 3 | 1 | 2 |
| DK0185 | 3 | 1 | 2 |
| DK0186 | 3 | 3 | 0 |
| DK0187 | 3 | 3 | 0 |
| DK0188 | 3 | 1 | 2 |
| DK0189 | 3 | 1 | 2 |
| DK0190 | 3 | 1 | 2 |
| DK0191 | 3 | 1 | 2 |
| DK0192 | 3 | 3 | 0 |
| G19002 | 3 | 1 | 2 |
| JB0004 | 3 | 1 | 2 |
| JB0006 | 3 | 1 | 2 |
| JB0007 | 3 | 1 | 2 |
| JB0008 | 3 | 1 | 2 |
| JB0009 | 3 | 1 | 2 |
| JB0010 | 3 | 1 | 2 |
| JB0011 | 3 | 1 | 2 |
| JB0012 | 3 | 1 | 2 |
| JB0013 | 3 | 1 | 2 |
| JB0014 | 3 | 1 | 2 |
| JB0015 | 3 | 1 | 2 |
| JB0016 | 3 | 1 | 2 |
| JB0017 | 3 | 1 | 2 |
| JB0018 | 3 | 1 | 2 |
| JB0020 | 3 | 1 | 2 |
| JB0021 | 3 | 1 | 2 |
| JB0023 | 3 | 1 | 2 |
| JB0024 | 3 | 1 | 2 |
| JB0025 | 3 | 1 | 2 |
| JB0026 | 3 | 1 | 2 |
| JB0027 | 3 | 1 | 2 |
| JB0028 | 3 | 1 | 2 |
| JB0029 | 3 | 1 | 2 |
| JB0030 | 3 | 1 | 2 |
| JB0031 | 3 | 1 | 2 |
| JB0032 | 3 | 1 | 2 |
| JB0033 | 3 | 1 | 2 |
| JB0034 | 3 | 1 | 2 |
| JB0035 | 3 | 1 | 2 |
| JB0036 | 3 | 1 | 2 |
| JB0037 | 3 | 1 | 2 |
| JB0038 | 3 | 1 | 2 |
| JB0039 | 3 | 1 | 2 |
| JB0040 | 3 | 1 | 2 |
| JB0041 | 3 | 1 | 2 |
| JB0042 | 3 | 1 | 2 |
| JB0043 | 3 | 1 | 2 |
| JB0044 | 3 | 1 | 2 |
| JB0045 | 3 | 1 | 2 |
| JB0046 | 3 | 1 | 2 |
| JB0048 | 3 | 1 | 2 |
| JB0049 | 3 | 1 | 2 |
| JB0050 | 3 | 1 | 2 |
| JB0052 | 3 | 1 | 2 |
| JB0053 | 3 | 1 | 2 |
| JB0054 | 3 | 1 | 2 |
| JB0056 | 3 | 1 | 2 |
| JB0057 | 3 | 1 | 2 |
| JB0058 | 3 | 1 | 2 |
| JB0059 | 3 | 1 | 2 |
| JB0060 | 3 | 1 | 2 |
| JB0062 | 3 | 1 | 2 |
| JB0064 | 3 | 1 | 2 |
| JB0068 | 3 | 1 | 2 |
| JB0069 | 3 | 1 | 2 |
| JB0070 | 3 | 1 | 2 |
| JB0071 | 3 | 1 | 2 |
| JB0072 | 3 | 1 | 2 |
| JB0073 | 3 | 1 | 2 |
| JB0075 | 3 | 1 | 2 |
| JB0077 | 3 | 1 | 2 |
| JB0078 | 3 | 1 | 2 |
| JB0079 | 3 | 1 | 2 |
| JB0080 | 3 | 1 | 2 |
| JB0081 | 3 | 1 | 2 |
| JB0082 | 3 | 1 | 2 |
| JB0083 | 3 | 1 | 2 |
| JB0084 | 3 | 1 | 2 |
| JB0085 | 3 | 1 | 2 |
| JB0086 | 3 | 1 | 2 |
| JB0088 | 3 | 1 | 2 |
| JB0089 | 3 | 1 | 2 |
| JB0090 | 3 | 1 | 2 |
| JB0091 | 3 | 1 | 2 |
| JB0092 | 3 | 1 | 2 |
| JB0095 | 3 | 1 | 2 |
| JB0096 | 3 | 1 | 2 |
| JB0097 | 3 | 1 | 2 |
| JB0098 | 3 | 1 | 2 |
| JB0100 | 3 | 1 | 2 |
| JB0101 | 3 | 1 | 2 |
| JB0102 | 3 | 1 | 2 |
| JB0103 | 3 | 1 | 2 |
| JB0104 | 3 | 1 | 2 |
| JB0105 | 3 | 1 | 2 |
| JB0107 | 3 | 1 | 2 |
| JB0108 | 3 | 1 | 2 |
| JB0110 | 3 | 1 | 2 |
| JB0111 | 3 | 1 | 2 |
| JB0112 | 3 | 1 | 2 |
| JB0113 | 3 | 1 | 2 |
| JB0114 | 3 | 1 | 2 |
| JB0115 | 3 | 1 | 2 |
| JB0116 | 3 | 1 | 2 |
| JB0117 | 3 | 1 | 2 |
| JB0118 | 3 | 1 | 2 |
| JB0119 | 3 | 1 | 2 |
| JB0121 | 3 | 1 | 2 |
| JB0122 | 3 | 1 | 2 |
| JB0123 | 3 | 1 | 2 |
| JB0124 | 3 | 1 | 2 |
| JB0125 | 3 | 1 | 2 |
| JB0126 | 3 | 1 | 2 |
| JB0127 | 3 | 1 | 2 |
| JB0128 | 3 | 1 | 2 |
| JB0129 | 3 | 1 | 2 |
| JB0130 | 3 | 1 | 2 |
| JB0132 | 3 | 1 | 2 |
| JB0135 | 3 | 1 | 2 |
| JB0136 | 3 | 1 | 2 |
| JB0137 | 3 | 1 | 2 |
| JB0138 | 3 | 1 | 2 |
| JB0139 | 3 | 1 | 2 |
| JB0140 | 3 | 1 | 2 |
| JB0141 | 3 | 1 | 2 |
| JB0142 | 3 | 1 | 2 |
| JB0143 | 3 | 1 | 2 |
| JB0144 | 3 | 1 | 2 |
| JB0145 | 3 | 1 | 2 |
| JB0146 | 3 | 1 | 2 |
| JB0147 | 3 | 1 | 2 |
| JB0148 | 3 | 1 | 2 |
| JB0149 | 3 | 1 | 2 |
| JB0150 | 3 | 1 | 2 |
| JB0151 | 3 | 1 | 2 |
| JB0152 | 3 | 1 | 2 |
| JB0154 | 3 | 1 | 2 |
| JB0155 | 3 | 1 | 2 |
| JB0156 | 3 | 1 | 2 |
| JB0157 | 3 | 1 | 2 |
| JB0158 | 3 | 1 | 2 |
| JB0160 | 3 | 1 | 2 |
| JB0161 | 3 | 1 | 2 |
| JB0164 | 3 | 1 | 2 |
| JB0165 | 3 | 1 | 2 |
| JB0166 | 3 | 1 | 2 |
| JB0167 | 3 | 1 | 2 |
| JB0168 | 3 | 1 | 2 |
| JB0169 | 3 | 1 | 2 |
| JB0170 | 3 | 1 | 2 |
| JB0172 | 3 | 1 | 2 |
| JB0173 | 3 | 1 | 2 |
| JB0174 | 3 | 1 | 2 |
| JB0175 | 3 | 1 | 2 |
| JB0177 | 3 | 1 | 2 |
| JB0179 | 3 | 1 | 2 |
| JB0180 | 3 | 1 | 2 |
| JB0181 | 3 | 1 | 2 |
| JB0182 | 3 | 1 | 2 |
| JB0183 | 3 | 1 | 2 |
| JB0186 | 3 | 1 | 2 |
| JB0187 | 3 | 1 | 2 |
| JB0188 | 3 | 1 | 2 |
| JB0189 | 3 | 1 | 2 |
| JB0190 | 3 | 1 | 2 |
| JB0191 | 3 | 1 | 2 |
| JB0192 | 3 | 1 | 2 |
| JB0193 | 3 | 1 | 2 |
| JB0197 | 3 | 1 | 2 |
| JB0199 | 3 | 1 | 2 |
| JB0200 | 3 | 1 | 2 |
| JB0202 | 3 | 1 | 2 |
| JB0204 | 3 | 1 | 2 |
| JB0205 | 3 | 1 | 2 |
| JB0206 | 3 | 1 | 2 |
| JB0207 | 3 | 1 | 2 |
| JB0208 | 3 | 1 | 2 |
| JB0209 | 3 | 1 | 2 |
| JB0211 | 3 | 1 | 2 |
| JB0212 | 3 | 1 | 2 |
| JB0213 | 3 | 1 | 2 |
| JB0214 | 3 | 1 | 2 |
| JB0215 | 3 | 1 | 2 |
| JB0216 | 3 | 1 | 2 |
| JB0220 | 3 | 1 | 2 |
| JB0221 | 3 | 1 | 2 |
| JB0222 | 3 | 1 | 2 |
| JB0223 | 3 | 1 | 2 |
| JB0224 | 3 | 1 | 2 |
| JB0225 | 3 | 1 | 2 |
| JB0226 | 3 | 1 | 2 |
| JB0227 | 3 | 1 | 2 |
| JB0228 | 3 | 1 | 2 |
| JB0232 | 3 | 1 | 2 |
| JB0233 | 3 | 1 | 2 |
| JB0234 | 3 | 1 | 2 |
| JB0235 | 3 | 1 | 2 |
| JB0236 | 3 | 1 | 2 |
| JB0237 | 3 | 1 | 2 |
| JB0238 | 3 | 1 | 2 |
| JB0240 | 3 | 1 | 2 |
| JB0241 | 3 | 1 | 2 |
| JB0242 | 3 | 1 | 2 |
| JB0243 | 3 | 1 | 2 |
| JB0244 | 3 | 1 | 2 |
| JB0246 | 3 | 1 | 2 |
| JB0247 | 3 | 1 | 2 |
| JB0248 | 3 | 1 | 2 |
| JB0249 | 3 | 1 | 2 |
| JB0250 | 3 | 1 | 2 |
| JB0251 | 3 | 1 | 2 |
| JB0252 | 3 | 1 | 2 |
| JB0253 | 3 | 1 | 2 |
| JB0254 | 3 | 1 | 2 |
| JB0255 | 3 | 1 | 2 |
| JB0256 | 3 | 1 | 2 |
| JB0257 | 3 | 1 | 2 |
| JB0258 | 3 | 1 | 2 |
| JB0259 | 3 | 1 | 2 |
| JB0260 | 3 | 1 | 2 |
| JB0261 | 3 | 1 | 2 |
| JB0262 | 3 | 1 | 2 |
| JB0263 | 3 | 1 | 2 |
| JB0264 | 3 | 1 | 2 |
| JB0265 | 3 | 1 | 2 |
| JB0267 | 3 | 1 | 2 |
| JB0268 | 3 | 1 | 2 |
| JB0269 | 3 | 1 | 2 |
| JB0270 | 3 | 1 | 2 |
| JB0271 | 3 | 1 | 2 |
| JB0272 | 3 | 1 | 2 |
| JB0273 | 3 | 1 | 2 |
| JB0274 | 3 | 1 | 2 |
| JB0275 | 3 | 1 | 2 |
| JB0276 | 3 | 1 | 2 |
| JB0277 | 3 | 1 | 2 |
| JB0278 | 3 | 1 | 2 |
| JB0279 | 3 | 1 | 2 |
| JB0280 | 3 | 1 | 2 |
| JB0281 | 3 | 1 | 2 |
| JB0282 | 3 | 1 | 2 |
| JB0283 | 3 | 1 | 2 |
| JB0284 | 3 | 1 | 2 |
| JB0285 | 3 | 1 | 2 |
| JB0286 | 3 | 1 | 2 |
| JB0288 | 3 | 1 | 2 |
| JB0289 | 3 | 1 | 2 |
| JB0290 | 3 | 1 | 2 |
| JB0291 | 3 | 1 | 2 |
| JB0292 | 3 | 1 | 2 |
| JB0293 | 3 | 1 | 2 |
| JB0294 | 3 | 1 | 2 |
| JB0295 | 3 | 1 | 2 |
| JB0296 | 3 | 1 | 2 |
| JB0297 | 3 | 1 | 2 |
| JB0298 | 3 | 1 | 2 |
| JB0300 | 3 | 1 | 2 |
| JB0301 | 3 | 1 | 2 |
| JB0302 | 3 | 1 | 2 |
| JB0304 | 3 | 1 | 2 |
| JB0306 | 3 | 1 | 2 |
| JB0307 | 3 | 1 | 2 |
| JB0309 | 3 | 1 | 2 |
| JB0310 | 3 | 1 | 2 |
| JB0311 | 3 | 1 | 2 |
| JB0313 | 3 | 1 | 2 |
| JB0314 | 3 | 1 | 2 |
| JB0315 | 3 | 1 | 2 |
| JB0316 | 3 | 1 | 2 |
| JB0317 | 3 | 1 | 2 |
| JB0318 | 3 | 1 | 2 |
| JB0319 | 3 | 1 | 2 |
| JB0320 | 3 | 1 | 2 |
| JB0321 | 3 | 1 | 2 |
| JB0322 | 3 | 1 | 2 |
| JB0323 | 3 | 1 | 2 |
| JB0325 | 3 | 1 | 2 |
| JB0326 | 3 | 1 | 2 |
| JB0327 | 3 | 1 | 2 |
| JB0329 | 3 | 1 | 2 |
| JB0330 | 3 | 1 | 2 |
| JB0331 | 3 | 1 | 2 |
| JB0332 | 3 | 1 | 2 |
| JB0333 | 3 | 1 | 2 |
| JB0334 | 3 | 1 | 2 |
| JB0335 | 3 | 1 | 2 |
| JB0336 | 3 | 1 | 2 |
| JB0337 | 3 | 1 | 2 |
| JB0338 | 3 | 1 | 2 |
| JB0339 | 3 | 1 | 2 |
| JB0340 | 3 | 1 | 2 |
| JB0341 | 3 | 1 | 2 |
| JB0342 | 3 | 1 | 2 |
| JB0343 | 3 | 1 | 2 |
| JB0345 | 3 | 1 | 2 |
| JB0346 | 3 | 1 | 2 |
| JB0348 | 3 | 1 | 2 |
| JB0349 | 3 | 1 | 2 |
| JB0350 | 3 | 1 | 2 |
| JB0351 | 3 | 1 | 2 |
| JB0352 | 3 | 1 | 2 |
| JB0353 | 3 | 1 | 2 |
| JB0354 | 3 | 1 | 2 |
| JB0355 | 3 | 1 | 2 |
| JB0356 | 3 | 1 | 2 |
| JB0357 | 3 | 1 | 2 |
| JB0359 | 3 | 1 | 2 |
| JB0360 | 3 | 1 | 2 |
| JB0361 | 3 | 1 | 2 |
| JB0362 | 3 | 1 | 2 |
| JB0363 | 3 | 1 | 2 |
| JB0364 | 3 | 1 | 2 |
| JB0365 | 3 | 1 | 2 |
| JB0366 | 3 | 1 | 2 |
| JB0368 | 3 | 1 | 2 |
| JB0369 | 3 | 1 | 2 |
| JB0370 | 3 | 1 | 2 |
| JB0371 | 3 | 1 | 2 |
| JB0372 | 3 | 1 | 2 |
| JB0373 | 3 | 1 | 2 |
| JB0374 | 3 | 1 | 2 |
| JB0377 | 3 | 1 | 2 |
| JB0378 | 3 | 1 | 2 |
| JB0379 | 3 | 1 | 2 |
| JB0380 | 3 | 1 | 2 |
| JB0381 | 3 | 1 | 2 |
| JB0383 | 3 | 1 | 2 |
| JB0384 | 3 | 1 | 2 |
| JB0385 | 3 | 1 | 2 |
| JB0387 | 3 | 1 | 2 |
| JB0389 | 3 | 1 | 2 |
| JB0390 | 3 | 1 | 2 |
| JB0392 | 3 | 1 | 2 |
| JB0393 | 3 | 1 | 2 |
| JB0394 | 3 | 1 | 2 |
| JB0396 | 3 | 1 | 2 |
| JB0397 | 3 | 1 | 2 |
| JB0398 | 3 | 1 | 2 |
| JB0399 | 3 | 1 | 2 |
| JB0400 | 3 | 1 | 2 |
| JB0401 | 3 | 1 | 2 |
| JB0402 | 3 | 1 | 2 |
| JB0404 | 3 | 1 | 2 |
| JB0405 | 3 | 1 | 2 |
| JB0406 | 3 | 1 | 2 |
| JB0407 | 3 | 1 | 2 |
| JB0408 | 3 | 1 | 2 |
| JB0409 | 3 | 1 | 2 |
| JB0410 | 3 | 1 | 2 |
| JB0412 | 3 | 1 | 2 |
| JB0413 | 3 | 1 | 2 |
| JB0414 | 3 | 1 | 2 |
| JB0416 | 3 | 1 | 2 |
| JB0418 | 3 | 1 | 2 |
| JB0419 | 3 | 1 | 2 |
| JB0420 | 3 | 1 | 2 |
| JB0421 | 3 | 1 | 2 |
| JB0423 | 3 | 1 | 2 |
| JB0424 | 3 | 1 | 2 |
| JB0425 | 3 | 1 | 2 |
| JB0426 | 3 | 1 | 2 |
| JB0427 | 3 | 1 | 2 |
| JB0428 | 3 | 1 | 2 |
| JB0430 | 3 | 1 | 2 |
| JB0431 | 3 | 1 | 2 |
| JB0432 | 3 | 1 | 2 |
| JB0433 | 3 | 1 | 2 |
| JB0434 | 3 | 1 | 2 |
| JB0435 | 3 | 1 | 2 |
| JB0436 | 3 | 1 | 2 |
| JB0437 | 3 | 1 | 2 |
| JB0438 | 3 | 1 | 2 |
| JB0439 | 3 | 1 | 2 |
| JB0440 | 3 | 1 | 2 |
| JB0441 | 3 | 1 | 2 |
| JB0442 | 3 | 1 | 2 |
| JB0444 | 3 | 1 | 2 |
| JB0445 | 3 | 1 | 2 |
| JB0446 | 3 | 1 | 2 |
| JB0447 | 3 | 1 | 2 |
| JB0448 | 3 | 1 | 2 |
| JB0449 | 3 | 1 | 2 |
| JB0450 | 3 | 1 | 2 |
| JB0452 | 3 | 1 | 2 |
| JB0454 | 3 | 1 | 2 |
| JB0456 | 3 | 1 | 2 |
| JB0458 | 3 | 1 | 2 |
| JB0459 | 3 | 1 | 2 |
| JB0462 | 3 | 1 | 2 |
| JB0463 | 3 | 1 | 2 |
| JB0464 | 3 | 1 | 2 |
| JB0465 | 3 | 1 | 2 |
| JB0466 | 3 | 1 | 2 |
| JB0467 | 3 | 1 | 2 |
| JB0468 | 3 | 1 | 2 |
| JB0469 | 3 | 1 | 2 |
| JB0470 | 3 | 1 | 2 |
| JB0471 | 3 | 1 | 2 |
| JB0472 | 3 | 1 | 2 |
| JB0473 | 3 | 1 | 2 |
| JB0474 | 3 | 1 | 2 |
| JB0475 | 3 | 1 | 2 |
| JB0476 | 3 | 1 | 2 |
| JB0478 | 3 | 1 | 2 |
| JB0479 | 3 | 1 | 2 |
| JB0481 | 3 | 1 | 2 |
| JB0482 | 3 | 1 | 2 |
| JB0483 | 3 | 1 | 2 |
| JB0484 | 3 | 1 | 2 |
| JB0487 | 3 | 1 | 2 |
| JB0488 | 3 | 1 | 2 |
| JB0490 | 3 | 1 | 2 |
| JB0491 | 3 | 1 | 2 |
| JB0492 | 3 | 1 | 2 |
| JB0493 | 3 | 1 | 2 |
| JB0494 | 3 | 1 | 2 |
| JB0496 | 3 | 1 | 2 |
| JB0498 | 3 | 1 | 2 |
| JB0500 | 3 | 1 | 2 |
| JB0502 | 3 | 1 | 2 |
| JB0503 | 3 | 1 | 2 |
| JB0504 | 3 | 1 | 2 |
| JB0505 | 3 | 1 | 2 |
| JB0506 | 3 | 1 | 2 |
| JB0507 | 3 | 1 | 2 |
| JB0508 | 3 | 1 | 2 |
| JB0509 | 3 | 1 | 2 |
| JB0510 | 3 | 1 | 2 |
| JB0511 | 3 | 1 | 2 |
| JB0512 | 3 | 1 | 2 |
| JB0513 | 3 | 1 | 2 |
| JB0514 | 3 | 1 | 2 |
| JB0515 | 3 | 1 | 2 |
| JB0516 | 3 | 1 | 2 |
| JB0518 | 3 | 1 | 2 |
| JB0519 | 3 | 1 | 2 |
| JB0520 | 3 | 1 | 2 |
| JB0521 | 3 | 1 | 2 |
| JB0522 | 3 | 1 | 2 |
| JB0523 | 3 | 1 | 2 |
| JB0524 | 3 | 1 | 2 |
| JB0525 | 3 | 1 | 2 |
| JB0526 | 3 | 1 | 2 |
| JB0527 | 3 | 1 | 2 |
| JB0528 | 3 | 1 | 2 |
| JB0529 | 3 | 1 | 2 |
| JB0530 | 3 | 1 | 2 |
| JB0531 | 3 | 1 | 2 |
| JB0532 | 3 | 1 | 2 |
| JB0533 | 3 | 1 | 2 |
| JB0534 | 3 | 1 | 2 |
| JB0535 | 3 | 1 | 2 |
| JB0536 | 3 | 1 | 2 |
| JB0537 | 3 | 1 | 2 |
| JB0538 | 3 | 1 | 2 |
| JB0539 | 3 | 1 | 2 |
| JB0540 | 3 | 1 | 2 |
| JB0541 | 3 | 1 | 2 |
| JB0542 | 3 | 1 | 2 |
| JB0543 | 3 | 1 | 2 |
| JB0544 | 3 | 1 | 2 |
| JB0545 | 3 | 1 | 2 |
| JB0547 | 3 | 1 | 2 |
| JB0548 | 3 | 1 | 2 |
| JB0549 | 3 | 1 | 2 |
| JB0550 | 3 | 1 | 2 |
| JB0551 | 3 | 1 | 2 |
| JB0552 | 3 | 1 | 2 |
| JB0553 | 3 | 1 | 2 |
| JB0554 | 3 | 1 | 2 |
| JB0555 | 3 | 1 | 2 |
| JB0556 | 3 | 1 | 2 |
| JB0557 | 3 | 1 | 2 |
| JB0558 | 3 | 1 | 2 |
| JB0559 | 3 | 1 | 2 |
| JB0562 | 3 | 1 | 2 |
| JB0563 | 3 | 1 | 2 |
| JB0564 | 3 | 1 | 2 |
| JB0565 | 3 | 1 | 2 |
| JB0566 | 3 | 1 | 2 |
| JB0567 | 3 | 1 | 2 |
| JB0568 | 3 | 1 | 2 |
| JB0569 | 3 | 1 | 2 |
| JB0570 | 3 | 1 | 2 |
| JB0571 | 3 | 1 | 2 |
| JB0572 | 3 | 1 | 2 |
| JB0573 | 3 | 1 | 2 |
| JB0575 | 3 | 1 | 2 |
| JB0576 | 3 | 1 | 2 |
| JB0577 | 3 | 1 | 2 |
| JB0578 | 3 | 1 | 2 |
| JB0579 | 3 | 1 | 2 |
| JB0580 | 3 | 1 | 2 |
| JB0581 | 3 | 1 | 2 |
| JB0582 | 3 | 1 | 2 |
| JB0583 | 3 | 1 | 2 |
| JB0584 | 3 | 1 | 2 |
| JB0585 | 3 | 1 | 2 |
| JB0586 | 3 | 1 | 2 |
| JB0587 | 3 | 1 | 2 |
| JB0588 | 3 | 1 | 2 |
| JB0589 | 3 | 1 | 2 |
| JB0590 | 3 | 1 | 2 |
| JB0591 | 3 | 1 | 2 |
| JB0592 | 3 | 1 | 2 |
| JB0593 | 3 | 1 | 2 |
| JB0594 | 3 | 1 | 2 |
| JB0595 | 3 | 1 | 2 |
| JB0596 | 3 | 1 | 2 |
| JB0597 | 3 | 1 | 2 |
| JB0598 | 3 | 1 | 2 |
| JB0599 | 3 | 1 | 2 |
| JB0600 | 3 | 1 | 2 |
| JB0602 | 3 | 1 | 2 |
| JB0604 | 3 | 1 | 2 |
| JB0606 | 3 | 1 | 2 |
| JB0607 | 3 | 1 | 2 |
| JB0608 | 3 | 1 | 2 |
| JB0609 | 3 | 1 | 2 |
| JB0610 | 3 | 1 | 2 |
| JB0611 | 3 | 1 | 2 |
| JB0612 | 3 | 1 | 2 |
| JB0613 | 3 | 1 | 2 |
| JB0614 | 3 | 1 | 2 |
| JB0615 | 3 | 1 | 2 |
| JB0616 | 3 | 1 | 2 |
| JB0617 | 3 | 1 | 2 |
| JB0618 | 3 | 1 | 2 |
| JB0619 | 3 | 1 | 2 |
| JB0621 | 3 | 1 | 2 |
| JB0622 | 3 | 1 | 2 |
| JB0623 | 3 | 1 | 2 |
| JB0624 | 3 | 1 | 2 |
| JB0625 | 3 | 1 | 2 |
| JB0626 | 3 | 1 | 2 |
| JB0627 | 3 | 1 | 2 |
| JB0628 | 3 | 1 | 2 |
| JB0629 | 3 | 1 | 2 |
| JB0630 | 3 | 1 | 2 |
| JB0631 | 3 | 1 | 2 |
| JB0632 | 3 | 1 | 2 |
| JB0634 | 3 | 1 | 2 |
| JB0635 | 3 | 1 | 2 |
| JB0636 | 3 | 1 | 2 |
| JB0637 | 3 | 1 | 2 |
| JB0638 | 3 | 1 | 2 |
| JB0639 | 3 | 1 | 2 |
| JB0640 | 3 | 1 | 2 |
| JB0641 | 3 | 1 | 2 |
| JB0643 | 3 | 1 | 2 |
| JB0644 | 3 | 1 | 2 |
| JB0645 | 3 | 1 | 2 |
| JB0646 | 3 | 1 | 2 |
| JB0647 | 3 | 1 | 2 |
| JB0650 | 3 | 1 | 2 |
| JB0651 | 3 | 1 | 2 |
| JB0652 | 3 | 1 | 2 |
| JB0653 | 3 | 1 | 2 |
| JB0654 | 3 | 1 | 2 |
| JB0655 | 3 | 1 | 2 |
| JB0656 | 3 | 1 | 2 |
| JB0657 | 3 | 1 | 2 |
| JB0658 | 3 | 1 | 2 |
| JB0660 | 3 | 1 | 2 |
| JB0661 | 3 | 1 | 2 |
| JB0662 | 3 | 1 | 2 |
| JB0663 | 3 | 1 | 2 |
| JB0665 | 3 | 1 | 2 |
| JB0666 | 3 | 1 | 2 |
| JB0667 | 3 | 1 | 2 |
| JB0668 | 3 | 1 | 2 |
| JB0669 | 3 | 1 | 2 |
| JB0670 | 3 | 1 | 2 |
| JB0671 | 3 | 1 | 2 |
| JB0673 | 3 | 1 | 2 |
| JB0674 | 3 | 1 | 2 |
| JB0675 | 3 | 1 | 2 |
| JB0676 | 3 | 1 | 2 |
| JB0677 | 3 | 1 | 2 |
| JB0678 | 3 | 1 | 2 |
| JB0679 | 3 | 1 | 2 |
| JB0680 | 3 | 1 | 2 |
| JB0681 | 3 | 1 | 2 |
| JB0682 | 3 | 1 | 2 |
| JB0683 | 3 | 1 | 2 |
| JB0684 | 3 | 1 | 2 |
| JB0685 | 3 | 1 | 2 |
| JB0686 | 3 | 1 | 2 |
| JB0687 | 3 | 1 | 2 |
| JB0688 | 3 | 1 | 2 |
| JB0689 | 3 | 1 | 2 |
| JB0690 | 3 | 1 | 2 |
| JB0691 | 3 | 1 | 2 |
| JB0692 | 3 | 1 | 2 |
| JB0693 | 3 | 1 | 2 |
| JB0694 | 3 | 1 | 2 |
| JB0695 | 3 | 1 | 2 |
| JB0696 | 3 | 1 | 2 |
| JB0697 | 3 | 1 | 2 |
| JB0699 | 3 | 1 | 2 |
| JB0700 | 3 | 1 | 2 |
| JB0701 | 3 | 1 | 2 |
| JB0702 | 3 | 1 | 2 |
| JB0703 | 3 | 1 | 2 |
| JB0704 | 3 | 1 | 2 |
| JB0705 | 3 | 1 | 2 |
| JB0706 | 3 | 1 | 2 |
| JB0707 | 3 | 1 | 2 |
| JB0708 | 3 | 1 | 2 |
| JB0709 | 3 | 1 | 2 |
| JB0710 | 3 | 1 | 2 |
| JB0711 | 3 | 1 | 2 |
| JB0712 | 3 | 1 | 2 |
| JB0713 | 3 | 1 | 2 |
| JB0714 | 3 | 1 | 2 |
| JB0715 | 3 | 1 | 2 |
| JB0716 | 3 | 1 | 2 |
| JB0717 | 3 | 1 | 2 |
| JB0718 | 3 | 1 | 2 |
| JB0719 | 3 | 1 | 2 |
| JB0720 | 3 | 1 | 2 |
| JB0721 | 3 | 1 | 2 |
| JB0722 | 3 | 1 | 2 |
| JB0727 | 3 | 1 | 2 |
| JB0728 | 3 | 1 | 2 |
| JB0733 | 3 | 1 | 2 |
| JB0734 | 3 | 1 | 2 |
| JB0735 | 3 | 1 | 2 |
| JB0736 | 3 | 1 | 2 |
| JB0737 | 3 | 1 | 2 |
| JB0738 | 3 | 1 | 2 |
| JB0739 | 3 | 1 | 2 |
| JB0740 | 3 | 1 | 2 |
| JB0741 | 3 | 1 | 2 |
| JB0742 | 3 | 1 | 2 |
| JB0743 | 3 | 1 | 2 |
| JB0745 | 3 | 1 | 2 |
| JB0746 | 3 | 1 | 2 |
| JB0747 | 3 | 1 | 2 |
| JB0748 | 3 | 1 | 2 |
| JB0749 | 3 | 1 | 2 |
| JB0750 | 3 | 1 | 2 |
| JB0752 | 3 | 1 | 2 |
| JB0753 | 3 | 1 | 2 |
| JB0754 | 3 | 1 | 2 |
| JB0755 | 3 | 1 | 2 |
| JB0756 | 3 | 1 | 2 |
| JB0758 | 3 | 1 | 2 |
| JB0759 | 3 | 1 | 2 |
| JB0760 | 3 | 1 | 2 |
| JB0761 | 3 | 1 | 2 |
| JB0766 | 3 | 1 | 2 |
| JB0767 | 3 | 1 | 2 |
| JB0768 | 3 | 1 | 2 |
| JB0769 | 3 | 1 | 2 |
| JB0770 | 3 | 1 | 2 |
| JB0771 | 3 | 1 | 2 |
| JB0772 | 3 | 1 | 2 |
| JB0773 | 3 | 1 | 2 |
| JB0774 | 3 | 1 | 2 |
| JB0775 | 3 | 1 | 2 |
| JB0776 | 3 | 1 | 2 |
| JB0777 | 3 | 1 | 2 |
| JB0778 | 3 | 1 | 2 |
| JB0779 | 3 | 1 | 2 |
| JB0780 | 3 | 1 | 2 |
| JB0781 | 3 | 1 | 2 |
| JB0782 | 3 | 1 | 2 |
| JB0783 | 3 | 1 | 2 |
| JB0784 | 3 | 1 | 2 |
| JB0785 | 3 | 1 | 2 |
| JB0786 | 3 | 1 | 2 |
| JB0787 | 3 | 1 | 2 |
| JB0788 | 3 | 1 | 2 |
| JB0789 | 3 | 1 | 2 |
| JB0790 | 3 | 1 | 2 |
| JB0791 | 3 | 1 | 2 |
| JB0792 | 3 | 1 | 2 |
| JB0793 | 3 | 1 | 2 |
| JB0794 | 3 | 1 | 2 |
| JB0795 | 3 | 1 | 2 |
| JB0796 | 3 | 1 | 2 |
| JB0797 | 3 | 1 | 2 |
| JB0798 | 3 | 1 | 2 |
| JB0799 | 3 | 1 | 2 |
| JB0800 | 3 | 1 | 2 |
| JB0801 | 3 | 1 | 2 |
| JB0802 | 3 | 1 | 2 |
| JB0803 | 3 | 1 | 2 |
| JB0804 | 3 | 1 | 2 |
| JB0805 | 3 | 1 | 2 |
| JB0806 | 3 | 1 | 2 |
| JB0807 | 3 | 1 | 2 |
| JB0808 | 3 | 1 | 2 |
| JB0809 | 3 | 1 | 2 |
| JB0810 | 3 | 1 | 2 |
| JB0811 | 3 | 1 | 2 |
| JB0812 | 3 | 1 | 2 |
| JB0813 | 3 | 1 | 2 |
| JB0814 | 3 | 1 | 2 |
| JB0817 | 3 | 1 | 2 |
| JB0818 | 3 | 1 | 2 |
| JB0819 | 3 | 1 | 2 |
| JB0820 | 3 | 1 | 2 |
| JB0821 | 3 | 1 | 2 |
| JB0822 | 3 | 1 | 2 |
| JB0823 | 3 | 1 | 2 |
| JB0824 | 3 | 1 | 2 |
| JB0825 | 3 | 1 | 2 |
| JB0826 | 3 | 1 | 2 |
| JB0827 | 3 | 1 | 2 |
| JB0828 | 3 | 1 | 2 |
| JB0829 | 3 | 1 | 2 |
| JB0830 | 3 | 1 | 2 |
| JB0831 | 3 | 1 | 2 |
| JB0832 | 3 | 1 | 2 |
| JB0833 | 3 | 1 | 2 |
| JB0834 | 3 | 1 | 2 |
| JB0835 | 3 | 1 | 2 |
| JB0837 | 3 | 1 | 2 |
| JB0838 | 3 | 1 | 2 |
| JB0840 | 3 | 1 | 2 |
| JB0841 | 3 | 1 | 2 |
| JB0842 | 3 | 1 | 2 |
| JB0843 | 3 | 1 | 2 |
| JB0844 | 3 | 1 | 2 |
| JB0846 | 3 | 1 | 2 |
| JB0847 | 3 | 1 | 2 |
| JB0848 | 3 | 1 | 2 |
| JB0849 | 3 | 1 | 2 |
| JB0850 | 3 | 1 | 2 |
| JB0851 | 3 | 1 | 2 |
| JB0852 | 3 | 1 | 2 |
| JB0853 | 3 | 1 | 2 |
| JB0854 | 3 | 1 | 2 |
| JB0855 | 3 | 1 | 2 |
| JB0856 | 3 | 1 | 2 |
| JB0857 | 3 | 1 | 2 |
| JB0858 | 3 | 1 | 2 |
| JB0859 | 3 | 1 | 2 |
| JB0861 | 3 | 1 | 2 |
| JB0863 | 3 | 1 | 2 |
| JB0864 | 3 | 1 | 2 |
| JB0865 | 3 | 1 | 2 |
| JB0867 | 3 | 1 | 2 |
| JB0868 | 3 | 1 | 2 |
| JB0870 | 3 | 1 | 2 |
| JB0871 | 3 | 1 | 2 |
| JB0872 | 3 | 1 | 2 |
| JB0873 | 3 | 1 | 2 |
| JB0874 | 3 | 1 | 2 |
| JB0875 | 3 | 1 | 2 |
| JB0876 | 3 | 1 | 2 |
| JB0877 | 3 | 1 | 2 |
| JB0878 | 3 | 1 | 2 |
| JB0879 | 3 | 1 | 2 |
| JB0881 | 3 | 1 | 2 |
| JB0882 | 3 | 1 | 2 |
| JB0883 | 3 | 1 | 2 |
| JB0884 | 3 | 1 | 2 |
| JB0885 | 3 | 1 | 2 |
| JB0886 | 3 | 1 | 2 |
| JB0889 | 3 | 1 | 2 |
| JB0890 | 3 | 1 | 2 |
| JB0891 | 3 | 1 | 2 |
| JB0893 | 3 | 1 | 2 |
| JB0894 | 3 | 1 | 2 |
| JB0895 | 3 | 1 | 2 |
| JB0896 | 3 | 1 | 2 |
| JB0897 | 3 | 1 | 2 |
| JB0898 | 3 | 1 | 2 |
| LB0001 | 3 | 2 | 1 |
| LB0002 | 3 | 2 | 1 |
| LB0003 | 3 | 2 | 1 |
| LB0004 | 3 | 2 | 1 |
| LB0005 | 3 | 2 | 1 |
| LB0006 | 3 | 2 | 1 |
| LB0007 | 3 | 2 | 1 |
| LB0008 | 3 | 2 | 1 |
| LB0009 | 3 | 3 | 0 |
| LB0010 | 3 | 2 | 1 |
| LB0011 | 3 | 3 | 0 |
| LB0012 | 3 | 2 | 1 |
| LB0013 | 3 | 2 | 1 |
| LB0014 | 3 | 2 | 1 |
| LB0015 | 3 | 2 | 1 |
| LB0016 | 3 | 2 | 1 |
| LB0017 | 3 | 3 | 0 |
| LB0018 | 3 | 2 | 1 |
| LB0019 | 3 | 2 | 1 |
| LB0020 | 3 | 2 | 1 |
| LB0021 | 3 | 2 | 1 |
| LB0022 | 3 | 2 | 1 |
| LB0023 | 3 | 2 | 1 |
| LB0024 | 3 | 1 | 2 |
| LB0025 | 3 | 1 | 2 |
| LB0026 | 3 | 3 | 0 |
| LB0027 | 3 | 2 | 1 |
| LB0028 | 3 | 2 | 1 |
| LB0029 | 3 | 2 | 1 |
| LB0030 | 3 | 3 | 0 |
| LB0032 | 3 | 1 | 2 |
| LB0035 | 3 | 2 | 1 |
| LB0036 | 3 | 2 | 1 |
| LB0037 | 3 | 3 | 0 |
| LB0038 | 3 | 1 | 2 |
| LB0039 | 3 | 1 | 2 |
| LB0040 | 3 | 3 | 0 |
| LB0041 | 3 | 1 | 2 |
| LB0042 | 3 | 2 | 1 |
| LB0043 | 3 | 3 | 0 |
| LB0044 | 3 | 3 | 0 |
| LB0045 | 3 | 2 | 1 |
| LB0046 | 3 | 2 | 1 |
| LB0047 | 3 | 3 | 0 |
| LB0049 | 3 | 1 | 2 |
| LB0050 | 3 | 3 | 0 |
| LB0051 | 3 | 1 | 2 |
| LB0052 | 3 | 3 | 0 |
| LB0053 | 3 | 1 | 2 |
| LB0054 | 3 | 2 | 1 |
| LB0055 | 3 | 1 | 2 |
| LB0056 | 3 | 3 | 0 |
| LB0057 | 3 | 1 | 2 |
| LB0058 | 3 | 2 | 1 |
| LB0059 | 3 | 2 | 1 |
| LB0060 | 3 | 3 | 0 |
| LB0061 | 3 | 2 | 1 |
| LB0062 | 3 | 1 | 2 |
| LB0064 | 3 | 3 | 0 |
| LB0065 | 3 | 1 | 2 |
| LB0066 | 3 | 3 | 0 |
| LB0067 | 3 | 1 | 2 |
| LB0068 | 3 | 3 | 0 |
| LB0069 | 3 | 3 | 0 |
| LB0070 | 3 | 3 | 0 |
| LB0071 | 3 | 3 | 0 |
| LB0072 | 3 | 1 | 2 |
| LB0074 | 3 | 3 | 0 |
| LB0076 | 3 | 2 | 1 |
| LB0077 | 3 | 1 | 2 |
| LB0078 | 3 | 1 | 2 |
| LB0079 | 3 | 3 | 0 |
| LB0079 | 3 | 3 | 0 |
| LB0080 | 3 | 3 | 0 |
| LB0081 | 3 | 3 | 0 |
| LB0082 | 3 | 2 | 1 |
| LB0083 | 3 | 1 | 2 |
| LB0084 | 3 | 3 | 0 |
| LB0085 | 3 | 1 | 2 |
| LB0086 | 3 | 1 | 2 |
| LB0087 | 3 | 2 | 1 |
| LB0088 | 3 | 3 | 0 |
| LB0089 | 3 | 1 | 2 |
| LB0090 | 3 | 3 | 0 |
| LB0092 | 3 | 1 | 2 |
| LB0096 | 3 | 1 | 2 |
| LB0099 | 3 | 1 | 2 |
| LB0100 | 3 | 1 | 2 |
| LB0101 | 3 | 1 | 2 |
| LB0104 | 3 | 3 | 0 |
| LB0105 | 3 | 3 | 0 |
| LB0106 | 3 | 1 | 2 |
| LB0107 | 3 | 1 | 2 |
| LB0108 | 3 | 1 | 2 |
| LB0109 | 3 | 3 | 0 |
| LB0111 | 3 | 1 | 2 |
| LB0112 | 3 | 1 | 2 |
| LB0113 | 3 | 1 | 2 |
| LB0114 | 3 | 1 | 2 |
| LB0115 | 3 | 1 | 2 |
| LB0116 | 3 | 1 | 2 |
| LB0117 | 3 | 1 | 2 |
| LB0118 | 3 | 1 | 2 |
| LB0119 | 3 | 1 | 2 |
| LB0120 | 3 | 3 | 0 |
| LB0121 | 3 | 3 | 0 |
| LB0122 | 3 | 3 | 0 |
| LB0123 | 3 | 3 | 0 |
| LB0125 | 3 | 3 | 0 |
| LB0126 | 3 | 1 | 2 |
| LB0127 | 3 | 1 | 2 |
| LB0128 | 3 | 1 | 2 |
| LB0129 | 3 | 1 | 2 |
| LB0130 | 3 | 1 | 2 |
| LB0131 | 3 | 3 | 0 |
| LB0132 | 3 | 3 | 0 |
| LB0133 | 3 | 1 | 2 |
| LB0134 | 3 | 1 | 2 |
| LB0135 | 3 | 1 | 2 |
| LB0136 | 3 | 1 | 2 |
| LB0137 | 3 | 1 | 2 |
| LB0138 | 3 | 1 | 2 |
| LB0142 | 3 | 2 | 1 |
| LB0143 | 3 | 1 | 2 |
| LB0144 | 3 | 1 | 2 |
| LB0145 | 3 | 1 | 2 |
| LB0146 | 3 | 1 | 2 |
| LB0147 | 3 | 3 | 0 |
| LB0148 | 3 | 1 | 2 |
| LB0149 | 3 | 2 | 1 |
| LB0150 | 3 | 2 | 1 |
| LB0151 | 3 | 2 | 1 |
| LB0152 | 3 | 3 | 0 |
| LB0153 | 3 | 3 | 0 |
| LB0154 | 3 | 3 | 0 |
| LB0158 | 3 | 1 | 2 |
| LB0159 | 3 | 1 | 2 |
| LB0160 | 3 | 1 | 2 |
| LB0161 | 3 | 2 | 1 |
| LB0162 | 3 | 1 | 2 |
| LB0163 | 3 | 1 | 2 |
| LB0164 | 3 | 3 | 0 |
| LB0165 | 3 | 2 | 1 |
| LB0166 | 3 | 2 | 1 |
| LB0167 | 3 | 2 | 1 |
| LB0168 | 3 | 2 | 1 |
| LB0169 | 3 | 2 | 1 |
| LB0170 | 3 | 2 | 1 |
| LB0171 | 3 | 1 | 2 |
| LB0172 | 3 | 2 | 1 |
| LB0173 | 3 | 2 | 1 |
| LB0174 | 3 | 2 | 1 |
| LB0176 | 3 | 2 | 1 |
| LB0177 | 3 | 3 | 0 |
| LB0178 | 3 | 3 | 0 |
| LB0179 | 3 | 1 | 2 |
| LB0180 | 3 | 1 | 2 |
| LB0181 | 3 | 1 | 2 |
| LB0182 | 3 | 1 | 2 |
| LB0183 | 3 | 1 | 2 |
| LB0184 | 3 | 3 | 0 |
| LB0185 | 3 | 1 | 2 |
| LB0186 | 3 | 1 | 2 |
| LB0187 | 3 | 1 | 2 |
| LB0188 | 3 | 3 | 0 |
| LB0189 | 3 | 3 | 0 |
| LB0190 | 3 | 1 | 2 |
| LB0195 | 3 | 3 | 0 |
| LB0196 | 3 | 1 | 2 |
| LB0197 | 3 | 1 | 2 |
| LB0198 | 3 | 2 | 1 |
| LB0199 | 3 | 3 | 0 |
| LB0202 | 3 | 2 | 1 |
| LB0203 | 3 | 2 | 1 |
| LB0205 | 3 | 3 | 0 |
| LB0206 | 3 | 3 | 0 |
| LB0207 | 3 | 2 | 1 |
| LB0208 | 3 | 3 | 0 |
| LB0209 | 3 | 2 | 1 |
| LB0210 | 3 | 1 | 2 |
| LB0211 | 3 | 2 | 1 |
| LB0212 | 3 | 2 | 1 |
| LB0213 | 3 | 1 | 2 |
| LB0214 | 3 | 1 | 2 |
| LB0215 | 3 | 1 | 2 |
| LB0216 | 3 | 1 | 2 |
| LB0217 | 3 | 3 | 0 |
| LB0218 | 3 | 1 | 2 |
| LB0219 | 3 | 3 | 0 |
| LB0220 | 3 | 3 | 0 |
| LB0220 | 3 | 3 | 0 |
| LB0221 | 3 | 3 | 0 |
| LB0222 | 3 | 1 | 2 |
| LB0223 | 3 | 1 | 2 |
| LB0224 | 3 | 3 | 0 |
| LB0225 | 3 | 1 | 2 |
| LB0226 | 3 | 1 | 2 |
| LB0227 | 3 | 1 | 2 |
| LB0228 | 3 | 1 | 2 |
| LB0229 | 3 | 1 | 2 |
| LB0231 | 3 | 1 | 2 |
| LB0232 | 3 | 2 | 1 |
| LB0234 | 3 | 2 | 1 |
| LB0235 | 3 | 1 | 2 |
| LB0237 | 3 | 1 | 2 |
| LB0238 | 3 | 1 | 2 |
| LB0239 | 3 | 1 | 2 |
| LB0240 | 3 | 1 | 2 |
| LB0241 | 3 | 2 | 1 |
| LB0242 | 3 | 1 | 2 |
| LB0246 | 3 | 3 | 0 |
| LB0247 | 3 | 1 | 2 |
| LB0248 | 3 | 3 | 0 |
| LB0249 | 3 | 2 | 1 |
| LB0250 | 3 | 2 | 1 |
| LB0251 | 3 | 1 | 2 |
| LB0254 | 3 | 1 | 2 |
| LB0255 | 3 | 2 | 1 |
| LB0257 | 3 | 1 | 2 |
| LB0258 | 3 | 1 | 2 |
| LB0259 | 3 | 1 | 2 |
| LB0260 | 3 | 2 | 1 |
| LB0261 | 3 | 1 | 2 |
| LB0262 | 3 | 3 | 0 |
| LB0264 | 3 | 1 | 2 |
| LB0268 | 3 | 1 | 2 |
| LB0270 | 3 | 1 | 2 |
| LB0271 | 3 | 2 | 1 |
| LB0272 | 3 | 2 | 1 |
| LB0273 | 3 | 1 | 2 |
| LB0274 | 3 | 3 | 0 |
| LB0275 | 3 | 2 | 1 |
| LB0276 | 3 | 1 | 2 |
| LB0278 | 3 | 1 | 2 |
| LB0279 | 3 | 1 | 2 |
| LB0280 | 3 | 1 | 2 |
| LB0281 | 3 | 2 | 1 |
| LB0283 | 3 | 1 | 2 |
| LB0284 | 3 | 2 | 1 |
| LB0285 | 3 | 1 | 2 |
| LB0286 | 3 | 1 | 2 |
| LB0287 | 3 | 1 | 2 |
| LB0288 | 3 | 2 | 1 |
| LB0289 | 3 | 1 | 2 |
| LB0290 | 3 | 3 | 0 |
| LB0292 | 3 | 1 | 2 |
| LB0293 | 3 | 2 | 1 |
| LB0294 | 3 | 3 | 0 |
| LB0295 | 3 | 3 | 0 |
| LB0296 | 3 | 1 | 2 |
| LB0297 | 3 | 3 | 0 |
| LB0297 | 3 | 3 | 0 |
| LB0299 | 3 | 1 | 2 |
| LB0300 | 3 | 2 | 1 |
| LB0301 | 3 | 1 | 2 |
| LB0302 | 3 | 1 | 2 |
| LB0304 | 3 | 1 | 2 |
| LB0305 | 3 | 1 | 2 |
| LB0307 | 3 | 1 | 2 |
| LB0308 | 3 | 2 | 1 |
| LB0309 | 3 | 2 | 1 |
| LB0310 | 3 | 1 | 2 |
| LB0311 | 3 | 2 | 1 |
| LB0312 | 3 | 2 | 1 |
| LB0313 | 3 | 1 | 2 |
| LB0314 | 3 | 3 | 0 |
| LB0315 | 3 | 3 | 0 |
| LB0316 | 3 | 1 | 2 |
| LB0317 | 3 | 2 | 1 |
| LB0318 | 3 | 1 | 2 |
| LB0319 | 3 | 1 | 2 |
| LB0320 | 3 | 1 | 2 |
| LB0322 | 3 | 1 | 2 |
| LB0333 | 3 | 1 | 2 |
| LB0334 | 3 | 2 | 1 |
| LB0335 | 3 | 3 | 0 |
| LB0336 | 3 | 1 | 2 |
| LB0337 | 3 | 1 | 2 |
| LB0338 | 3 | 1 | 2 |
| LB0339 | 3 | 3 | 0 |
| LB0340 | 3 | 1 | 2 |
| LB0341 | 3 | 1 | 2 |
| LB0342 | 3 | 3 | 0 |
| LB0343 | 3 | 1 | 2 |
| LB0344 | 3 | 1 | 2 |
| LB0345 | 3 | 1 | 2 |
| LB0346 | 3 | 3 | 0 |
| LB0349 | 3 | 2 | 1 |
| LB0350 | 3 | 2 | 1 |
| LB0351 | 3 | 2 | 1 |
| LB0352 | 3 | 1 | 2 |
| LB0353 | 3 | 1 | 2 |
| LB0354 | 3 | 1 | 2 |
| LB0355 | 3 | 3 | 0 |
| LB0356 | 3 | 2 | 1 |
| LB0357 | 3 | 2 | 1 |
| LB0359 | 3 | 2 | 1 |
| LB0360 | 3 | 2 | 1 |
| LB0361 | 3 | 2 | 1 |
| LB0362 | 3 | 1 | 2 |
| LB0363 | 3 | 1 | 2 |
| LB0364 | 3 | 1 | 2 |
| LB0365 | 3 | 1 | 2 |
| LB0366 | 3 | 3 | 0 |
| LB0367 | 3 | 2 | 1 |
| LB0369 | 3 | 2 | 1 |
| LB0372 | 3 | 3 | 0 |
| LB0373 | 3 | 3 | 0 |
| LB0374 | 3 | 3 | 0 |
| LB0375 | 3 | 3 | 0 |
| LB0376 | 3 | 3 | 0 |
| LB0378 | 3 | 3 | 0 |
| LB0379 | 3 | 3 | 0 |
| LB0380 | 3 | 1 | 2 |
| LB0381 | 3 | 1 | 2 |
| LB0382 | 3 | 1 | 2 |
| LB0383 | 3 | 2 | 1 |
| LB0385 | 3 | 2 | 1 |
| LB0386 | 3 | 1 | 2 |
| LB0387 | 3 | 1 | 2 |
| LB0388 | 3 | 2 | 1 |
| LB0390 | 3 | 2 | 1 |
| LB0391 | 3 | 1 | 2 |
| LB0392 | 3 | 1 | 2 |
| LB0393 | 3 | 2 | 1 |
| LB0394 | 3 | 1 | 2 |
| LB0395 | 3 | 3 | 0 |
| LB0396 | 3 | 3 | 0 |
| LB0397 | 3 | 1 | 2 |
| LB0398 | 3 | 1 | 2 |
| LB0399 | 3 | 1 | 2 |
| LB0401 | 3 | 1 | 2 |
| LB0402 | 3 | 3 | 0 |
| LB0403 | 3 | 3 | 0 |
| LB0404 | 3 | 1 | 2 |
| LB0405 | 3 | 1 | 2 |
| LB0406 | 3 | 1 | 2 |
| LB0408 | 3 | 3 | 0 |
| LB0409 | 3 | 1 | 2 |
| LB0410 | 3 | 1 | 2 |
| LB0411 | 3 | 3 | 0 |
| LB0412 | 3 | 3 | 0 |
| LB0413 | 3 | 1 | 2 |
| LB0414 | 3 | 1 | 2 |
| LB0416 | 3 | 1 | 2 |
| LB0417 | 3 | 1 | 2 |
| LB0418 | 3 | 1 | 2 |
| LB0419 | 3 | 3 | 0 |
| LB0420 | 3 | 1 | 2 |
| LB0421 | 3 | 1 | 2 |
| LB0422 | 3 | 1 | 2 |
| LB0423 | 3 | 1 | 2 |
| LB0424 | 3 | 1 | 2 |
| LB0425 | 3 | 3 | 0 |
| LB0426 | 3 | 1 | 2 |
| LB0427 | 3 | 1 | 2 |
| LB0428 | 3 | 1 | 2 |
| LB0429 | 3 | 1 | 2 |
| LB0430 | 3 | 1 | 2 |
| LB0431 | 3 | 1 | 2 |
| LB0432 | 3 | 1 | 2 |
| LB0433 | 3 | 1 | 2 |
| LB0434 | 3 | 3 | 0 |
| LB0435 | 3 | 1 | 2 |
| LB0436 | 3 | 2 | 1 |
| LB0437 | 3 | 1 | 2 |
| LB0438 | 3 | 2 | 1 |
| LB0439 | 3 | 2 | 1 |
| LB0440 | 3 | 1 | 2 |
| LB0441 | 3 | 1 | 2 |
| LB0442 | 3 | 1 | 2 |
| LB0443 | 3 | 1 | 2 |
| LB0444 | 3 | 1 | 2 |
| LB0446 | 3 | 2 | 1 |
| LB0447 | 3 | 2 | 1 |
| LB0448 | 3 | 2 | 1 |
| LB0449 | 3 | 2 | 1 |
| LB0450 | 3 | 2 | 1 |
| LB0451 | 3 | 2 | 1 |
| LB0452 | 3 | 2 | 1 |
| LB0453 | 3 | 2 | 1 |
| LB0454 | 3 | 2 | 1 |
| LB0455 | 3 | 2 | 1 |
| LB0456 | 3 | 2 | 1 |
| LB0457 | 3 | 2 | 1 |
| LB0458 | 3 | 2 | 1 |
| LB0459 | 3 | 2 | 1 |
| LB0460 | 3 | 2 | 1 |
| LB0461 | 3 | 2 | 1 |
| LB0462 | 3 | 2 | 1 |
| LB0463 | 3 | 2 | 1 |
| LB0464 | 3 | 2 | 1 |
| LB0465 | 3 | 2 | 1 |
| LB0466 | 3 | 2 | 1 |
| LB0467 | 3 | 2 | 1 |
| LB0468 | 3 | 2 | 1 |
| LB0469 | 3 | 2 | 1 |
| LB0470 | 3 | 1 | 2 |
| LB0471 | 3 | 1 | 2 |
| LB0473 | 3 | 1 | 2 |
| LB0475 | 3 | 2 | 1 |
| LB0476 | 3 | 2 | 1 |
| LB0477 | 3 | 1 | 2 |
| LB0478 | 3 | 2 | 1 |
| LB0479 | 3 | 2 | 1 |
| LB0480 | 3 | 2 | 1 |
| LB0481 | 3 | 1 | 2 |
| LB0482 | 3 | 1 | 2 |
| LB0483 | 3 | 2 | 1 |
| LB0484 | 3 | 2 | 1 |
| LB0485 | 3 | 2 | 1 |
| LB0486 | 3 | 2 | 1 |
| LB0487 | 3 | 1 | 2 |
| LB0488 | 3 | 2 | 1 |
| LB0489 | 3 | 2 | 1 |
| LB0490 | 3 | 1 | 2 |
| LB0491 | 3 | 1 | 2 |
| LB0492 | 3 | 1 | 2 |
| LB0493 | 3 | 1 | 2 |
| LB0494 | 3 | 1 | 2 |
| LB0495 | 3 | 2 | 1 |
| LB0496 | 3 | 2 | 1 |
| LB0498 | 3 | 2 | 1 |
| LB0499 | 3 | 2 | 1 |
| LB0500 | 3 | 2 | 1 |
| LB0501 | 3 | 2 | 1 |
| LB0502 | 3 | 2 | 1 |
| LB0503 | 3 | 2 | 1 |
| LB0504 | 3 | 2 | 1 |
| LB0505 | 3 | 2 | 1 |
| LB0506 | 3 | 2 | 1 |
| LB0507 | 3 | 2 | 1 |
| LB0508 | 3 | 2 | 1 |
| LB0509 | 3 | 2 | 1 |
| LB0510 | 3 | 1 | 2 |
| LB0511 | 3 | 1 | 2 |
| LB0512 | 3 | 1 | 2 |
| LB0513 | 3 | 1 | 2 |
| LB0514 | 3 | 2 | 1 |
| LB0517 | 3 | 2 | 1 |
| LB0518 | 3 | 2 | 1 |
| LB0519 | 3 | 2 | 1 |
| LB0523 | 3 | 2 | 1 |
| LB0524 | 3 | 2 | 1 |
| LB0525 | 3 | 2 | 1 |
| LB0526 | 3 | 2 | 1 |
| LB0527 | 3 | 2 | 1 |
| LB0528 | 3 | 2 | 1 |
| LB0530 | 3 | 1 | 2 |
| LB0531 | 3 | 2 | 1 |
| LB0532 | 3 | 2 | 1 |
| LB0533 | 3 | 1 | 2 |
| LB0535 | 3 | 2 | 1 |
| LB0536 | 3 | 2 | 1 |
| LB0537 | 3 | 2 | 1 |
| LB0538 | 3 | 2 | 1 |
| LB0539 | 3 | 1 | 2 |
| LB0544 | 3 | 2 | 1 |
| LB0545 | 3 | 1 | 2 |
| LB0547 | 3 | 2 | 1 |
| LB0548 | 3 | 1 | 2 |
| LB0549 | 3 | 1 | 2 |
| LB0550 | 3 | 2 | 1 |
| LB0551 | 3 | 1 | 2 |
| LB0553 | 3 | 1 | 2 |
| LB0557 | 3 | 1 | 2 |
| LB0558 | 3 | 2 | 1 |
| LB0561 | 3 | 1 | 2 |
| LB0562 | 3 | 2 | 1 |
| LB0563 | 3 | 1 | 2 |
| LB0564 | 3 | 1 | 2 |
| LB0565 | 3 | 1 | 2 |
| LB0566 | 3 | 1 | 2 |
| LB0567 | 3 | 1 | 2 |
| LB0568 | 3 | 1 | 2 |
| LB0569 | 3 | 1 | 2 |
| LB0596 | 3 | 1 | 2 |
| LB0597 | 3 | 2 | 1 |
| LB0598 | 3 | 1 | 2 |
| LB0599 | 3 | 2 | 1 |
| LB0600 | 3 | 2 | 1 |
| LB0601 | 3 | 2 | 1 |
| LB0602 | 3 | 2 | 1 |
| LB0603 | 3 | 2 | 1 |
| LB0604 | 3 | 2 | 1 |
| LB0605 | 3 | 2 | 1 |
| LB0606 | 3 | 2 | 1 |
| LB0607 | 3 | 2 | 1 |
| LB0L05 | 3 | 3 | 0 |
| LB0L06 | 3 | 3 | 0 |
| LB0L14 | 3 | 3 | 0 |
| LB0L23 | 3 | 3 | 0 |
| LB0L24 | 3 | 2 | 1 |
| LB2282 | 3 | 1 | 2 |
| LB2283 | 3 | 2 | 1 |
| MD0001 | 3 | 1 | 2 |
| MD0002 | 3 | 1 | 2 |
| MD0003 | 3 | 1 | 2 |
| MD0033 | 3 | 3 | 0 |
| MD0004 | 3 | 1 | 2 |
| MD0005 | 3 | 1 | 2 |
| MD0006 | 3 | 1 | 2 |
| MD0069 | 3 | 3 | 0 |
| MD0007 | 3 | 1 | 2 |
| MD0071 | 3 | 3 | 0 |
| MD0008 | 3 | 1 | 2 |
| MD0010 | 3 | 1 | 2 |
| MD0012 | 3 | 1 | 2 |
| MD0013 | 3 | 3 | 0 |
| MD0014 | 3 | 1 | 2 |
| MD0016 | 3 | 1 | 2 |
| MD0021 | 3 | 1 | 2 |
| MD0024 | 3 | 1 | 2 |
| MD0025 | 3 | 1 | 2 |
| MD0026 | 3 | 1 | 2 |
| MD0028 | 3 | 1 | 2 |
| MD0029 | 3 | 1 | 2 |
| MD0029 | 3 | 1 | 2 |
| MD0030 | 3 | 1 | 2 |
| MD0031 | 3 | 1 | 2 |
| MD0032 | 3 | 1 | 2 |
| MD0033 | 3 | 1 | 2 |
| MD0034 | 3 | 1 | 2 |
| MD0035 | 3 | 1 | 2 |
| MD0036 | 3 | 1 | 2 |
| MD0037 | 3 | 1 | 2 |
| MD0039 | 3 | 1 | 2 |
| MD0042 | 3 | 1 | 2 |
| MD0044 | 3 | 1 | 2 |
| MD0045 | 3 | 1 | 2 |
| MD0046 | 3 | 1 | 2 |
| MD0047 | 3 | 1 | 2 |
| MD0048 | 3 | 1 | 2 |
| MD0049 | 3 | 1 | 2 |
| MD0056 | 3 | 1 | 2 |
| MD0058 | 3 | 1 | 2 |
| MD0059 | 3 | 1 | 2 |
| MD0060 | 3 | 1 | 2 |
| MD0061 | 3 | 1 | 2 |
| MD0063 | 3 | 1 | 2 |
| MD0064 | 3 | 1 | 2 |
| MD0065 | 3 | 1 | 2 |
| MD0066 | 3 | 1 | 2 |
| MD0067 | 3 | 1 | 2 |
| MD0068 | 3 | 1 | 2 |
| MD0069 | 3 | 1 | 2 |
| MD0071 | 3 | 1 | 2 |
| MD0072 | 3 | 1 | 2 |
| MD0073 | 3 | 1 | 2 |
| MD0074 | 3 | 1 | 2 |
| MD0075 | 3 | 1 | 2 |
| MD0076 | 3 | 1 | 2 |
| MD0077 | 3 | 1 | 2 |
| MD0078 | 3 | 1 | 2 |
| MD0081 | 3 | 1 | 2 |
| MD0082 | 3 | 1 | 2 |
| MD0083 | 3 | 1 | 2 |
| MD0085 | 3 | 1 | 2 |
| MD0087 | 3 | 1 | 2 |
| MD0089 | 3 | 1 | 2 |
| MD0090 | 3 | 1 | 2 |
| MD0091 | 3 | 1 | 2 |
| MD0092 | 3 | 1 | 2 |
| MD0093 | 3 | 1 | 2 |
| MD0094 | 3 | 1 | 2 |
| MD0095 | 3 | 1 | 2 |
| MD0096 | 3 | 1 | 2 |
| MD0097 | 3 | 1 | 2 |
| MD0098 | 3 | 1 | 2 |
| MD0099 | 3 | 1 | 2 |
| MD0100 | 3 | 1 | 2 |
| MD0101 | 3 | 1 | 2 |
| MD0102 | 3 | 1 | 2 |
| MD0103 | 3 | 1 | 2 |
| MD0104 | 3 | 1 | 2 |
| MD0105 | 3 | 1 | 2 |
| MD0106 | 3 | 1 | 2 |
| MD0106 | 3 | 1 | 2 |
| MD0107 | 3 | 1 | 2 |
| MD0107 | 3 | 1 | 2 |
| MD0108 | 3 | 1 | 2 |
| MD0109 | 3 | 1 | 2 |
| MD0109 | 3 | 1 | 2 |
| MD0110 | 3 | 1 | 2 |
| MD0111 | 3 | 1 | 2 |
| MD0111 | 3 | 1 | 2 |
| MD0112 | 3 | 1 | 2 |
| MD0113 | 3 | 1 | 2 |
| MD0114 | 3 | 1 | 2 |
| MD0115 | 3 | 1 | 2 |
| MD0116 | 3 | 1 | 2 |
| MD0117 | 3 | 1 | 2 |
| MD0118 | 3 | 1 | 2 |
| MD0118 | 3 | 1 | 2 |
| MD0119 | 3 | 1 | 2 |
| MD0119 | 3 | 1 | 2 |
| MD0120 | 3 | 1 | 2 |
| MD0121 | 3 | 1 | 2 |
| MD0122 | 3 | 1 | 2 |
| MD0123 | 3 | 1 | 2 |
| MD0124 | 3 | 1 | 2 |
| MD0125 | 3 | 1 | 2 |
| MD0126 | 3 | 1 | 2 |
| MD0127 | 3 | 1 | 2 |
| MD0128 | 3 | 1 | 2 |
| MD0129 | 3 | 1 | 2 |
| MD0129 | 3 | 1 | 2 |
| MD0130 | 3 | 1 | 2 |
| MD0131 | 3 | 1 | 2 |
| MD0132 | 3 | 1 | 2 |
| MD0133 | 3 | 1 | 2 |
| MD0134 | 3 | 1 | 2 |
| MD0135 | 3 | 1 | 2 |
| MD0136 | 3 | 1 | 2 |
| MD0137 | 3 | 1 | 2 |
| MD0138 | 3 | 1 | 2 |
| MD0139 | 3 | 1 | 2 |
| MD0140 | 3 | 1 | 2 |
| MD0142 | 3 | 1 | 2 |
| MD0143 | 3 | 1 | 2 |
| MD0144 | 3 | 1 | 2 |
| MD0145 | 3 | 1 | 2 |
| MD0145 | 3 | 1 | 2 |
| MD0146 | 3 | 1 | 2 |
| MD0147 | 3 | 1 | 2 |
| MD0150 | 3 | 1 | 2 |
| MD0152 | 3 | 1 | 2 |
| MD0153 | 3 | 1 | 2 |
| MD0154 | 3 | 1 | 2 |
| MD0155 | 3 | 1 | 2 |
| MD0156 | 3 | 1 | 2 |
| MD0157 | 3 | 1 | 2 |
| MD0158 | 3 | 1 | 2 |
| MD0159 | 3 | 1 | 2 |
| MD0161 | 3 | 1 | 2 |
| MD0162 | 3 | 1 | 2 |
| MD0163 | 3 | 1 | 2 |
| MD0167 | 3 | 1 | 2 |
| MD0169 | 3 | 1 | 2 |
| MD0170 | 3 | 1 | 2 |
| MD0171 | 3 | 1 | 2 |
| MD0172 | 3 | 1 | 2 |
| MD0173 | 3 | 1 | 2 |
| MD0174 | 3 | 1 | 2 |
| MD0175 | 3 | 1 | 2 |
| MD0176 | 3 | 1 | 2 |
| MD0177 | 3 | 1 | 2 |
| MD0178 | 3 | 1 | 2 |
| MD0179 | 3 | 1 | 2 |
| MD0180 | 3 | 1 | 2 |
| MD0181 | 3 | 1 | 2 |
| MD0182 | 3 | 1 | 2 |
| MD0183 | 3 | 1 | 2 |
| MD0184 | 3 | 1 | 2 |
| MD0185 | 3 | 1 | 2 |
| MD0186 | 3 | 1 | 2 |
| MD0187 | 3 | 1 | 2 |
| MD0188 | 3 | 1 | 2 |
| MD0189 | 3 | 1 | 2 |
| MD0190 | 3 | 1 | 2 |
| MD0191 | 3 | 1 | 2 |
| MD0192 | 3 | 1 | 2 |
| MD0193 | 3 | 1 | 2 |
| MD0194 | 3 | 1 | 2 |
| MD0198 | 3 | 1 | 2 |
| MD0199 | 3 | 1 | 2 |
| MD0200 | 3 | 1 | 2 |
| MD0201 | 3 | 1 | 2 |
| MK0001 | 3 | 1 | 2 |
| MK0002 | 3 | 3 | 0 |
| MK0003 | 3 | 2 | 1 |
| MK0004 | 3 | 3 | 0 |
| MK0005 | 3 | 2 | 1 |
| MK0006 | 3 | 1 | 2 |
| MK0008 | 3 | 1 | 2 |
| MK0009 | 3 | 2 | 1 |
| MK0010 | 3 | 1 | 2 |
| MK0011 | 3 | 1 | 2 |
| MK0012 | 3 | 1 | 2 |
| MK0013 | 3 | 3 | 0 |
| MK0014 | 3 | 1 | 2 |
| MK0015 | 3 | 1 | 2 |
| MK0016 | 3 | 1 | 2 |
| MK0041 | 3 | 3 | 0 |
| MK0061 | 3 | 1 | 2 |
| MK0104 | 3 | 3 | 0 |
| MK0129 | 3 | 3 | 0 |
| MK0130 | 3 | 1 | 2 |
| MK0131 | 3 | 1 | 2 |
| MK0132 | 3 | 1 | 2 |
| MK0133 | 3 | 2 | 1 |
| MK0215 | 3 | 3 | 0 |
| MK0231 | 3 | 2 | 1 |
| MK0236 | 3 | 1 | 2 |
| MK0243 | 3 | 1 | 2 |
| MK0244 | 3 | 3 | 0 |
| MK0245 | 3 | 1 | 2 |
| MK0327 | 3 | 3 | 0 |
| MK0334 | 3 | 3 | 0 |
| MK0336 | 3 | 2 | 1 |
| MK0344 | 3 | 1 | 2 |
| MK0358 | 3 | 2 | 1 |
| MK0359 | 3 | 1 | 2 |
| MK0361 | 3 | 3 | 0 |
| MK0362 | 3 | 1 | 2 |
| MK0363 | 3 | 3 | 0 |
| MK0390 | 3 | 2 | 1 |
| MK0392 | 3 | 3 | 0 |
| MK0393 | 3 | 2 | 1 |
| MK0394 | 3 | 2 | 1 |
| MK0406 | 3 | 3 | 0 |
| MK0421 | 3 | 2 | 1 |
| MK0423 | 3 | 3 | 0 |
| MK0424 | 3 | 1 | 2 |
| MK0425 | 3 | 1 | 2 |
| MK0426 | 3 | 2 | 1 |
| MK0427 | 3 | 2 | 1 |
| MK0428 | 3 | 1 | 2 |
| MK0430 | 3 | 1 | 2 |
| MK0431 | 3 | 1 | 2 |
| MK0432 | 3 | 1 | 2 |
| MK0433 | 3 | 1 | 2 |
| MK0434 | 3 | 1 | 2 |
| MK0435 | 3 | 3 | 0 |
| MK0438 | 3 | 1 | 2 |
| MK0439 | 3 | 3 | 0 |
| MK0440 | 3 | 2 | 1 |
| MK0441 | 3 | 2 | 1 |
| MK0442 | 3 | 3 | 0 |
| MK0443 | 3 | 1 | 2 |
| MK0444 | 3 | 1 | 2 |
| MK0445 | 3 | 3 | 0 |
| MK0447 | 3 | 1 | 2 |
| MK0448 | 3 | 2 | 1 |
| MK0449 | 3 | 2 | 1 |
| MK0450 | 3 | 1 | 2 |
| MK0451 | 3 | 2 | 1 |
| MK0452 | 3 | 2 | 1 |
| MK0453 | 3 | 2 | 1 |
| MK0454 | 3 | 3 | 0 |
| MK0456 | 3 | 1 | 2 |
| MK0457 | 3 | 1 | 2 |
| MK0458 | 3 | 1 | 2 |
| MK0459 | 3 | 2 | 1 |
| MK0460 | 3 | 1 | 2 |
| MK0461 | 3 | 2 | 1 |
| MK0462 | 3 | 1 | 2 |
| MK0463 | 3 | 1 | 2 |
| MK0464 | 3 | 3 | 0 |
| MK0465 | 3 | 2 | 1 |
| MK0466 | 3 | 2 | 1 |
| MK0467 | 3 | 1 | 2 |
| MK0469 | 3 | 1 | 2 |
| MK0470 | 3 | 3 | 0 |
| MK0471 | 3 | 3 | 0 |
| MK0472 | 3 | 1 | 2 |
| MK0473 | 3 | 3 | 0 |
| MK0474 | 3 | 3 | 0 |
| MK0475 | 3 | 3 | 0 |
| MK0476 | 3 | 3 | 0 |
| MK0477 | 3 | 2 | 1 |
| MK0478 | 3 | 1 | 2 |
| MK0479 | 3 | 2 | 1 |
| MK0480 | 3 | 2 | 1 |
| MK0481 | 3 | 1 | 2 |
| MK0482 | 3 | 1 | 2 |
| MK0483 | 3 | 1 | 2 |
| MK0484 | 3 | 1 | 2 |
| MK0485 | 3 | 1 | 2 |
| MK0486 | 3 | 1 | 2 |
| MK0487 | 3 | 1 | 2 |
| MK0488 | 3 | 3 | 0 |
| MK0489 | 3 | 3 | 0 |
| MK0490 | 3 | 3 | 0 |
| MK0492 | 3 | 3 | 0 |
| MK0493 | 3 | 2 | 1 |
| MK0494 | 3 | 1 | 2 |
| MK0496 | 3 | 1 | 2 |
| MK0497 | 3 | 1 | 2 |
| MK0498 | 3 | 1 | 2 |
| MK0500 | 3 | 1 | 2 |
| MK0501 | 3 | 3 | 0 |
| MK0502 | 3 | 2 | 1 |
| MK0522 | 3 | 3 | 0 |
| MK0532 | 3 | 3 | 0 |
| MK0533 | 3 | 2 | 1 |
| MK0534 | 3 | 2 | 1 |
| MK0535 | 3 | 2 | 1 |
| MK0536 | 3 | 2 | 1 |
| MK0537 | 3 | 2 | 1 |
| MK0539 | 3 | 2 | 1 |
| MK0540 | 3 | 1 | 2 |
| MK0541 | 3 | 1 | 2 |
| MK0542 | 3 | 1 | 2 |
| MK0543 | 3 | 2 | 1 |
| MK0544 | 3 | 2 | 1 |
| MK0545 | 3 | 1 | 2 |
| MK0546 | 3 | 1 | 2 |
| MK0547 | 3 | 1 | 2 |
| MK0548 | 3 | 1 | 2 |
| MK0549 | 3 | 1 | 2 |
| MK0550 | 3 | 1 | 2 |
| MK0551 | 3 | 2 | 1 |
| MK0552 | 3 | 2 | 1 |
| MK0553 | 3 | 1 | 2 |
| MK0561 | 3 | 2 | 1 |
| MK0563 | 3 | 1 | 2 |
| MK0564 | 3 | 1 | 2 |
| MK0566 | 3 | 1 | 2 |
| MK0567 | 3 | 1 | 2 |
| MK0568 | 3 | 2 | 1 |
| MK0569 | 3 | 1 | 2 |
| MK0577 | 3 | 1 | 2 |
| MK0578 | 3 | 1 | 2 |
| MK0641 | 3 | 3 | 0 |
| MK0661 | 3 | 1 | 2 |
| MK0662 | 3 | 3 | 0 |
| MK0663 | 3 | 1 | 2 |
| MK0671 | 3 | 1 | 2 |
| MK0672 | 3 | 1 | 2 |
| MK0673 | 3 | 1 | 2 |
| MK0674 | 3 | 3 | 0 |
| MK0675 | 3 | 3 | 0 |
| MK0676 | 3 | 1 | 2 |
| MK0678 | 3 | 1 | 2 |
| MK0679 | 3 | 1 | 2 |
| MK0680 | 3 | 3 | 0 |
| MK0685 | 3 | 3 | 0 |
| MK0686 | 3 | 3 | 0 |
| MK0693 | 3 | 1 | 2 |
| MK0694 | 3 | 1 | 2 |
| MK0700 | 3 | 2 | 1 |
| MK0704 | 3 | 1 | 2 |
| MK0705 | 3 | 1 | 2 |
| MK0706 | 3 | 1 | 2 |
| MK0707 | 3 | 1 | 2 |
| MK0708 | 3 | 1 | 2 |
| MK0713 | 3 | 1 | 2 |
| MK0714 | 3 | 3 | 0 |
| MK0715 | 3 | 3 | 0 |
| MK0716 | 3 | 3 | 0 |
| MK0717 | 3 | 1 | 2 |
| MK0719 | 3 | 3 | 0 |
| MK0720 | 3 | 1 | 2 |
| MK0721 | 3 | 1 | 2 |
| MK0734 | 3 | 3 | 0 |
| MK0747 | 3 | 3 | 0 |
| MK0748 | 3 | 1 | 2 |
| MK0749 | 3 | 3 | 0 |
| MK0750 | 3 | 3 | 0 |
| MK0751 | 3 | 3 | 0 |
| MK0752 | 3 | 2 | 1 |
| MK0753 | 3 | 3 | 0 |
| MK0754 | 3 | 3 | 0 |
| MK0755 | 3 | 1 | 2 |
| MK0756 | 3 | 1 | 2 |
| MK0757 | 3 | 3 | 0 |
| MK0758 | 3 | 3 | 0 |
| MK0759 | 3 | 1 | 2 |
| MK0760 | 3 | 2 | 1 |
| MK0761 | 3 | 1 | 2 |
| MK0762 | 3 | 3 | 0 |
| MK0763 | 3 | 1 | 2 |
| MK0765 | 3 | 1 | 2 |
| MK0766 | 3 | 3 | 0 |
| MK0767 | 3 | 3 | 0 |
| MK0768 | 3 | 3 | 0 |
| MK0769 | 3 | 3 | 0 |
| MK0770 | 3 | 3 | 0 |
| MK0771 | 3 | 1 | 2 |
| MK0772 | 3 | 1 | 2 |
| MK0773 | 3 | 1 | 2 |
| MK0774 | 3 | 3 | 0 |
| MK0775 | 3 | 3 | 0 |
| MK0776 | 3 | 3 | 0 |
| MK0777 | 3 | 1 | 2 |
| MK0778 | 3 | 3 | 0 |
| MK0779 | 3 | 3 | 0 |
| MK0780 | 3 | 3 | 0 |
| MK0781 | 3 | 3 | 0 |
| MK0782 | 3 | 1 | 2 |
| MK0783 | 3 | 1 | 2 |
| MK0784 | 3 | 1 | 2 |
| MK0785 | 3 | 1 | 2 |
| MK0786 | 3 | 1 | 2 |
| MK0787 | 3 | 1 | 2 |
| MK0788 | 3 | 1 | 2 |
| MK0789 | 3 | 3 | 0 |
| MK0791 | 3 | 2 | 1 |
| MK0792 | 3 | 3 | 0 |
| MK0793 | 3 | 2 | 1 |
| MK0796 | 3 | 3 | 0 |
| MK0797 | 3 | 3 | 0 |
| MK0798 | 3 | 1 | 2 |
| MK0799 | 3 | 1 | 2 |
| MK0800 | 3 | 3 | 0 |
| MK0801 | 3 | 3 | 0 |
| MK0802 | 3 | 1 | 2 |
| MK0803 | 3 | 1 | 2 |
| MK0804 | 3 | 1 | 2 |
| MK0805 | 3 | 3 | 0 |
| MK0806 | 3 | 3 | 0 |
| MK0807 | 3 | 3 | 0 |
| MK0808 | 3 | 3 | 0 |
| MK0809 | 3 | 3 | 0 |
| MK0810 | 3 | 3 | 0 |
| MK0811 | 3 | 1 | 2 |
| MK0812 | 3 | 1 | 2 |
| MK0813 | 3 | 1 | 2 |
| MK0815 | 3 | 3 | 0 |
| MK0816 | 3 | 2 | 1 |
| MK0817 | 3 | 3 | 0 |
| MK0818 | 3 | 1 | 2 |
| MK0819 | 3 | 1 | 2 |
| MK0820 | 3 | 1 | 2 |
| MK0821 | 3 | 1 | 2 |
| MK0822 | 3 | 1 | 2 |
| MK0823 | 3 | 3 | 0 |
| MK0824 | 3 | 1 | 2 |
| MK0825 | 3 | 2 | 1 |
| MK0826 | 3 | 2 | 1 |
| MK0827 | 3 | 2 | 1 |
| MK0828 | 3 | 1 | 2 |
| MK0829 | 3 | 1 | 2 |
| MK0830 | 3 | 1 | 2 |
| MK0831 | 3 | 1 | 2 |
| MK0832 | 3 | 1 | 2 |
| MK0833 | 3 | 1 | 2 |
| MK0834 | 3 | 1 | 2 |
| MK0835 | 3 | 3 | 0 |
| MK0836 | 3 | 1 | 2 |
| MK0837 | 3 | 1 | 2 |
| MK0838 | 3 | 1 | 2 |
| MK0839 | 3 | 3 | 0 |
| MK0840 | 3 | 3 | 0 |
| MK0841 | 3 | 2 | 1 |
| MK0842 | 3 | 3 | 0 |
| MK0843 | 3 | 1 | 2 |
| MK0844 | 3 | 1 | 2 |
| MK0845 | 3 | 3 | 0 |
| MK0846 | 3 | 1 | 2 |
| MK0847 | 3 | 1 | 2 |
| MK0849 | 3 | 2 | 1 |
| MK0850 | 3 | 2 | 1 |
| MK0851 | 3 | 3 | 0 |
| MK0852 | 3 | 2 | 1 |
| MK0853 | 3 | 1 | 2 |
| MK0854 | 3 | 1 | 2 |
| MK0855 | 3 | 3 | 0 |
| MK0856 | 3 | 1 | 2 |
| MK0857 | 3 | 1 | 2 |
| MK0858 | 3 | 1 | 2 |
| MK0859 | 3 | 3 | 0 |
| MK0860 | 3 | 1 | 2 |
| MK0861 | 3 | 3 | 0 |
| MK0862 | 3 | 3 | 0 |
| MK0863 | 3 | 2 | 1 |
| MK0864 | 3 | 2 | 1 |
| MK0865 | 3 | 3 | 0 |
| MK0866 | 3 | 3 | 0 |
| MK0867 | 3 | 1 | 2 |
| MK0868 | 3 | 1 | 2 |
| MK0869 | 3 | 3 | 0 |
| MK0870 | 3 | 3 | 0 |
| MK0871 | 3 | 3 | 0 |
| MK0872 | 3 | 2 | 1 |
| MK0873 | 3 | 2 | 1 |
| MK0874 | 3 | 3 | 0 |
| MK0875 | 3 | 3 | 0 |
| MK0876 | 3 | 3 | 0 |
| MK0877 | 3 | 1 | 2 |
| MK0878 | 3 | 2 | 1 |
| MK0879 | 3 | 1 | 2 |
| MK0880 | 3 | 1 | 2 |
| MK0881 | 3 | 1 | 2 |
| MK0882 | 3 | 3 | 0 |
| MK0883 | 3 | 1 | 2 |
| MK0884 | 3 | 1 | 2 |
| MK0885 | 3 | 3 | 0 |
| MK0886 | 3 | 3 | 0 |
| MK0887 | 3 | 3 | 0 |
| MK0888 | 3 | 1 | 2 |
| MK0889 | 3 | 1 | 2 |
| MK0890 | 3 | 1 | 2 |
| MK0891 | 3 | 3 | 0 |
| MK0892 | 3 | 1 | 2 |
| MK0893 | 3 | 3 | 0 |
| MK0894 | 3 | 3 | 0 |
| MK0895 | 3 | 1 | 2 |
| MK0896 | 3 | 1 | 2 |
| MK0897 | 3 | 3 | 0 |
| MK0898 | 3 | 1 | 2 |
| MK0900 | 3 | 1 | 2 |
| MK0901 | 3 | 1 | 2 |
| MK0902 | 3 | 3 | 0 |
| MK0903 | 3 | 2 | 1 |
| MK0904 | 3 | 2 | 1 |
| MK0905 | 3 | 1 | 2 |
| MK0906 | 3 | 1 | 2 |
| MK0908 | 3 | 1 | 2 |
| MK0911 | 3 | 1 | 2 |
| MK0912 | 3 | 1 | 2 |
| MK0914 | 3 | 1 | 2 |
| MK0915 | 3 | 1 | 2 |
| MK0916 | 3 | 1 | 2 |
| MK0919 | 3 | 3 | 0 |
| MK0920 | 3 | 1 | 2 |
| MK0921 | 3 | 1 | 2 |
| MK0922 | 3 | 3 | 0 |
| MK0923 | 3 | 3 | 0 |
| MK0924 | 3 | 3 | 0 |
| MK0929 | 3 | 3 | 0 |
| MK0930 | 3 | 3 | 0 |
| MK0931 | 3 | 3 | 0 |
| MK0932 | 3 | 3 | 0 |
| MK0933 | 3 | 1 | 2 |
| MK0934 | 3 | 2 | 1 |
| MK0935 | 3 | 3 | 0 |
| MK0936 | 3 | 3 | 0 |
| MK0937 | 3 | 1 | 2 |
| MK0938 | 3 | 3 | 0 |
| MK0939 | 3 | 3 | 0 |
| MK0940 | 3 | 2 | 1 |
| MK0941 | 3 | 3 | 0 |
| MK0942 | 3 | 1 | 2 |
| MK0943 | 3 | 1 | 2 |
| MK0944 | 3 | 3 | 0 |
| MK0945 | 3 | 3 | 0 |
| MK0946 | 3 | 3 | 0 |
| MK0947 | 3 | 1 | 2 |
| MK0948 | 3 | 1 | 2 |
| MK0949 | 3 | 1 | 2 |
| MK0952 | 3 | 3 | 0 |
| MK0953 | 3 | 1 | 2 |
| MK0954 | 3 | 1 | 2 |
| MK0955 | 3 | 1 | 2 |
| MK0956 | 3 | 1 | 2 |
| MK0957 | 3 | 3 | 0 |
| MK0958 | 3 | 3 | 0 |
| MK0959 | 3 | 2 | 1 |
| MK0960 | 3 | 3 | 0 |
| MK0961 | 3 | 3 | 0 |
| MK0962 | 3 | 2 | 1 |
| MK0963 | 3 | 1 | 2 |
| MK0965 | 3 | 3 | 0 |
| MK0966 | 3 | 2 | 1 |
| MK0967 | 3 | 1 | 2 |
| MK0968 | 3 | 2 | 1 |
| MK0969 | 3 | 1 | 2 |
| MK1002 | 3 | 3 | 0 |
| MK1003 | 3 | 3 | 0 |
| MK1004 | 3 | 3 | 0 |
| MK1005 | 3 | 2 | 1 |
| MK1006 | 3 | 3 | 0 |
| MK1007 | 3 | 3 | 0 |
| MK1008 | 3 | 3 | 0 |
| MK1010 | 3 | 3 | 0 |
| MK1011 | 3 | 3 | 0 |
| MK1012 | 3 | 1 | 2 |
| MK1013 | 3 | 3 | 0 |
| MK1014 | 3 | 2 | 1 |
| MK1015 | 3 | 1 | 2 |
| MK1016 | 3 | 1 | 2 |
| MK1017 | 3 | 1 | 2 |
| MK1018 | 3 | 3 | 0 |
| MK1019 | 3 | 3 | 0 |
| MK1020 | 3 | 3 | 0 |
| MK1021 | 3 | 1 | 2 |
| MK1022 | 3 | 1 | 2 |
| MK1023 | 3 | 3 | 0 |
| MK1024 | 3 | 3 | 0 |
| MK1025 | 3 | 3 | 0 |
| MK1026 | 3 | 2 | 1 |
| MK1027 | 3 | 3 | 0 |
| MK1028 | 3 | 2 | 1 |
| MK1029 | 3 | 3 | 0 |
| MK1030 | 3 | 3 | 0 |
| MK1031 | 3 | 2 | 1 |
| MK1032 | 3 | 2 | 1 |
| MK1033 | 3 | 1 | 2 |
| MK1034 | 3 | 1 | 2 |
| MK1035 | 3 | 1 | 2 |
| MK1036 | 3 | 3 | 0 |
| MK1037 | 3 | 2 | 1 |
| MK1038 | 3 | 3 | 0 |
| MK1039 | 3 | 1 | 2 |
| MK1040 | 3 | 1 | 2 |
| MK1041 | 3 | 3 | 0 |
| MK1042 | 3 | 3 | 0 |
| MK1043 | 3 | 3 | 0 |
| MK1044 | 3 | 2 | 1 |
| MK1045 | 3 | 1 | 2 |
| MK1046 | 3 | 3 | 0 |
| MK1047 | 3 | 1 | 2 |
| MK1048 | 3 | 1 | 2 |
| MK1049 | 3 | 1 | 2 |
| MK1050 | 3 | 1 | 2 |
| MK1051 | 3 | 3 | 0 |
| MK1111 | 3 | 3 | 0 |
| MK1149 | 3 | 3 | 0 |
| MK1151 | 3 | 2 | 1 |
| MK1152 | 3 | 3 | 0 |
| MK1153 | 3 | 2 | 1 |
| MK1154 | 3 | 1 | 2 |
| MK1155 | 3 | 2 | 1 |
| MK1156 | 3 | 3 | 0 |
| MK1157 | 3 | 3 | 0 |
| MK1158 | 3 | 3 | 0 |
| MK1159 | 3 | 2 | 1 |
| MK1160 | 3 | 3 | 0 |
| MK1161 | 3 | 1 | 2 |
| MK1162 | 3 | 2 | 1 |
| MK1163 | 3 | 3 | 0 |
| MK1164 | 3 | 2 | 1 |
| MK1165 | 3 | 3 | 0 |
| MK1166 | 3 | 3 | 0 |
| MK1167 | 3 | 1 | 2 |
| MK1168 | 3 | 3 | 0 |
| MK1169 | 3 | 3 | 0 |
| MK1170 | 3 | 3 | 0 |
| MK1171 | 3 | 3 | 0 |
| MK1172 | 3 | 3 | 0 |
| MK1173 | 3 | 3 | 0 |
| MK1174 | 3 | 3 | 0 |
| MK1175 | 3 | 3 | 0 |
| MK1176 | 3 | 2 | 1 |
| MK1177 | 3 | 2 | 1 |
| MK1178 | 3 | 3 | 0 |
| MK1179 | 3 | 1 | 2 |
| MK1180 | 3 | 1 | 2 |
| MK1181 | 3 | 2 | 1 |
| MK1182 | 3 | 3 | 0 |
| MK1183 | 3 | 3 | 0 |
| MK1184 | 3 | 1 | 2 |
| MK1185 | 3 | 1 | 2 |
| MK1186 | 3 | 3 | 0 |
| MK1187 | 3 | 3 | 0 |
| MK1188 | 3 | 3 | 0 |
| MK1189 | 3 | 3 | 0 |
| MK1190 | 3 | 3 | 0 |
| MK1191 | 3 | 3 | 0 |
| MK1192 | 3 | 3 | 0 |
| MK1193 | 3 | 2 | 1 |
| MK1194 | 3 | 3 | 0 |
| MK1195 | 3 | 1 | 2 |
| MK1196 | 3 | 1 | 2 |
| MK1197 | 3 | 3 | 0 |
| MK1198 | 3 | 3 | 0 |
| MK1199 | 3 | 3 | 0 |
| MM0001 | 3 | 3 | 0 |
| MM0002 | 3 | 2 | 1 |
| MM0003 | 3 | 2 | 1 |
| MM0004 | 3 | 2 | 1 |
| MM0005 | 3 | 2 | 1 |
| MM0006 | 3 | 2 | 1 |
| MM0007 | 3 | 2 | 1 |
| MM0009 | 3 | 2 | 1 |
| MM0010 | 3 | 2 | 1 |
| MM0012 | 3 | 3 | 0 |
| MM0013 | 3 | 2 | 1 |
| MM0014 | 3 | 2 | 1 |
| MM0015 | 3 | 2 | 1 |
| MM0016 | 3 | 2 | 1 |
| MM0017 | 3 | 2 | 1 |
| MM0018 | 3 | 3 | 0 |
| MM0019 | 3 | 2 | 1 |
| MM0020 | 3 | 2 | 1 |
| MM0021 | 3 | 2 | 1 |
| MM0022 | 3 | 2 | 1 |
| MM0023 | 3 | 2 | 1 |
| MM0024 | 3 | 2 | 1 |
| MM0026 | 3 | 3 | 0 |
| MM0027 | 3 | 2 | 1 |
| MM0029 | 3 | 1 | 2 |
| MM0030 | 3 | 2 | 1 |
| MM0031 | 3 | 2 | 1 |
| MM0032 | 3 | 2 | 1 |
| MM0033 | 3 | 2 | 1 |
| MM0034 | 3 | 3 | 0 |
| MM0035 | 3 | 3 | 0 |
| MM0036 | 3 | 3 | 0 |
| MM0037 | 3 | 2 | 1 |
| MM0038 | 3 | 3 | 0 |
| MM0039 | 3 | 2 | 1 |
| MM0040 | 3 | 3 | 0 |
| MM0041 | 3 | 3 | 0 |
| MM0044 | 3 | 2 | 1 |
| MM0045 | 3 | 2 | 1 |
| MM0047 | 3 | 1 | 2 |
| MM0048 | 3 | 2 | 1 |
| MM0051 | 3 | 2 | 1 |
| MM0053 | 3 | 2 | 1 |
| MM0054 | 3 | 2 | 1 |
| MM0055 | 3 | 2 | 1 |
| MM0056 | 3 | 2 | 1 |
| MM0057 | 3 | 1 | 2 |
| MM0058 | 3 | 3 | 0 |
| MM0059 | 3 | 3 | 0 |
| MM0060 | 3 | 2 | 1 |
| MM0061 | 3 | 2 | 1 |
| MW0001 | 3 | 3 | 0 |
| MW0002 | 3 | 3 | 0 |
| MW0003 | 3 | 3 | 0 |
| MW0004 | 3 | 3 | 0 |
| MW0005 | 3 | 2 | 1 |
| MW0006 | 3 | 3 | 0 |
| MW0007 | 3 | 3 | 0 |
| MW0008 | 3 | 3 | 0 |
| MW0009 | 3 | 3 | 0 |
| MW0010 | 3 | 2 | 1 |
| MW0011 | 3 | 2 | 1 |
| MW0012 | 3 | 1 | 2 |
| MW0013 | 3 | 3 | 0 |
| MW0014 | 3 | 2 | 1 |
| MW0015 | 3 | 1 | 2 |
| MW0016 | 3 | 2 | 1 |
| MW0017 | 3 | 1 | 2 |
| MW0018 | 3 | 3 | 0 |
| MW0019 | 3 | 3 | 0 |
| MW0020 | 3 | 3 | 0 |
| MW0021 | 3 | 3 | 0 |
| MW0022 | 3 | 1 | 2 |
| MW0023 | 3 | 3 | 0 |
| MW0024 | 3 | 3 | 0 |
| MW0025 | 3 | 3 | 0 |
| MW0026 | 3 | 3 | 0 |
| MW0027 | 3 | 2 | 1 |
| MW0028 | 3 | 2 | 1 |
| MW0029 | 3 | 1 | 2 |
| MW0030 | 3 | 2 | 1 |
| MW0031 | 3 | 3 | 0 |
| MW0032 | 3 | 3 | 0 |
| MW0033 | 3 | 2 | 1 |
| MW0036 | 3 | 2 | 1 |
| MW0037 | 3 | 3 | 0 |
| MW0038 | 3 | 3 | 0 |
| MW0042 | 3 | 3 | 0 |
| MW0043 | 3 | 3 | 0 |
| R18006a | 3 | 1 | 2 |
| R18006b | 3 | 1 | 2 |
| R18018a | 3 | 1 | 2 |
| R18018b | 3 | 1 | 2 |
| R18023c | 3 | 1 | 2 |
| R18026c | 3 | 1 | 2 |
| R18V41 | 3 | 1 | 2 |
| R19_A3674 | 3 | 1 | 2 |
| R19_A3675 | 3 | 1 | 2 |
| R19_A3676 | 3 | 1 | 2 |
| R19_A3679 | 3 | 1 | 2 |
| R19_A3680 | 3 | 1 | 2 |
| R19_A3681 | 3 | 1 | 2 |
| R19_A3684 | 3 | 1 | 2 |
| R19_A3686 | 3 | 1 | 2 |
| R19_A3687 | 3 | 1 | 2 |
| R19_A3690 | 3 | 1 | 2 |
| R19_A3691 | 3 | 1 | 2 |
| R19_A3694 | 3 | 1 | 2 |
| R19_A3698 | 3 | 1 | 2 |
| R19_A3700 | 3 | 1 | 2 |
| R19_A3701 | 3 | 1 | 2 |
| R19003 | 3 | 1 | 2 |
| R19003 | 3 | 1 | 2 |
| R19005 | 3 | 1 | 2 |
| R19005 | 3 | 1 | 2 |
| R19007 | 3 | 1 | 2 |
| R19007 | 3 | 1 | 2 |
| R19011 | 3 | 1 | 2 |
| R19011 | 3 | 1 | 2 |
| R19015 | 3 | 1 | 2 |
| R19015 | 3 | 1 | 2 |
| R19016 | 3 | 1 | 2 |
| R19016 | 3 | 1 | 2 |
| R19020 | 3 | 1 | 2 |
| R19020 | 3 | 1 | 2 |
| R19021 | 3 | 1 | 2 |
| R19021 | 3 | 1 | 2 |
| R19023 | 3 | 1 | 2 |
| R19023 | 3 | 1 | 2 |
| R19033 | 3 | 1 | 2 |
| R19033 | 3 | 1 | 2 |
| R19034 | 3 | 1 | 2 |
| R19034 | 3 | 1 | 2 |
| R19036 | 3 | 1 | 2 |
| R19036 | 3 | 1 | 2 |
| TV0001 | 3 | 2 | 1 |
| TV0002 | 3 | 1 | 2 |
| TV0003 | 3 | 2 | 1 |
| TV0004 | 3 | 2 | 1 |
| TV0005 | 3 | 2 | 1 |
| TV0006 | 3 | 3 | 0 |
| TV0007 | 3 | 3 | 0 |
| TV0008 | 3 | 3 | 0 |
| TV0009 | 3 | 2 | 1 |
| TV0010 | 3 | 3 | 0 |
| TV0012 | 3 | 3 | 0 |
| TV0013 | 3 | 3 | 0 |
| TV0014 | 3 | 3 | 0 |
| TV0017 | 3 | 2 | 1 |
| TV0018 | 3 | 3 | 0 |
| TV0019 | 3 | 2 | 1 |
| TV0020 | 3 | 3 | 0 |
| TV0021 | 3 | 3 | 0 |
| TV0022 | 3 | 3 | 0 |
| TV0023 | 3 | 3 | 0 |
| TV0024 | 3 | 3 | 0 |
| TV0025 | 3 | 3 | 0 |
| TV0026 | 3 | 3 | 0 |
| TV0027 | 3 | 3 | 0 |
| TV0028 | 3 | 3 | 0 |
| TV0030 | 3 | 3 | 0 |
| TV0031 | 3 | 3 | 0 |
| TV0032 | 3 | 3 | 0 |
| TV0033 | 3 | 3 | 0 |
| TV0036 | 3 | 3 | 0 |
| TV0037 | 3 | 3 | 0 |
| TV0038 | 3 | 3 | 0 |
| TV0039 | 3 | 2 | 1 |
| TV0040 | 3 | 3 | 0 |
| TV0041 | 3 | 3 | 0 |
| TV0042 | 3 | 3 | 0 |
| TV0043 | 3 | 3 | 0 |
| TV0044 | 3 | 3 | 0 |
| TV0045 | 3 | 3 | 0 |
| TV0046 | 3 | 3 | 0 |
| TV0047 | 3 | 3 | 0 |
| TV0048 | 3 | 3 | 0 |
| TV0049 | 3 | 3 | 0 |
| TV0050 | 3 | 2 | 1 |
| TV0051 | 3 | 2 | 1 |
| TV0052 | 3 | 2 | 1 |
| TV0053 | 3 | 3 | 0 |
| TV0054 | 3 | 3 | 0 |
| TV0055 | 3 | 3 | 0 |
| TV0059 | 3 | 3 | 0 |
| TV0060 | 3 | 3 | 0 |
| TV0061 | 3 | 3 | 0 |
| TV0062 | 3 | 3 | 0 |
| TV0063 | 3 | 3 | 0 |
| TV0064 | 3 | 3 | 0 |
| TV0065 | 3 | 3 | 0 |
| TV0066 | 3 | 3 | 0 |
| TV0067 | 3 | 3 | 0 |
| TV0068 | 3 | 3 | 0 |
| TV0069 | 3 | 3 | 0 |
| TV0070 | 3 | 3 | 0 |
| TV0071 | 3 | 3 | 0 |
| TV0072 | 3 | 3 | 0 |
| TV0073 | 3 | 3 | 0 |
| TV0074 | 3 | 3 | 0 |
| TV0075 | 3 | 3 | 0 |
| TV0076 | 3 | 3 | 0 |
| TV0077 | 3 | 2 | 1 |
| TV0078 | 3 | 3 | 0 |
| TV0079 | 3 | 3 | 0 |
| TV0080 | 3 | 2 | 1 |
| TV0081 | 3 | 3 | 0 |
| TV0082 | 3 | 3 | 0 |
| TV0083 | 3 | 2 | 1 |
| TV0084 | 3 | 3 | 0 |
| TV0085 | 3 | 3 | 0 |
| TV0086 | 3 | 3 | 0 |
| TV0087 | 3 | 3 | 0 |
| TV0088 | 3 | 3 | 0 |
| TV0089 | 3 | 2 | 1 |
| TV0090 | 3 | 3 | 0 |
| TV0091 | 3 | 1 | 2 |
| TV0092 | 3 | 3 | 0 |
| TV0093 | 3 | 3 | 0 |
| TV0094 | 3 | 3 | 0 |
| TV0095 | 3 | 2 | 1 |
| TV0096 | 3 | 3 | 0 |
| VK0001 | 3 | 2 | 1 |
| VK0002 | 3 | 3 | 0 |
| VK0003 | 3 | 2 | 1 |
| VK0004 | 3 | 2 | 1 |
| VK0005 | 3 | 2 | 1 |
| VK0006 | 3 | 3 | 0 |
| VK0007 | 3 | 2 | 1 |
| VK0008 | 3 | 2 | 1 |
| VK0009 | 3 | 3 | 0 |
| VK0010 | 3 | 3 | 0 |
| VK0011 | 3 | 2 | 1 |
| VK0012 | 3 | 3 | 0 |
| VK0013 | 3 | 2 | 1 |
| VK0014 | 3 | 3 | 0 |
| VK0015 | 3 | 2 | 1 |
| VK0016 | 3 | 3 | 0 |
| VK0017 | 3 | 2 | 1 |
| VK0018 | 3 | 2 | 1 |
| VK0019 | 3 | 2 | 1 |
| VK0028 | 3 | 2 | 1 |
| VK0029 | 3 | 2 | 1 |
| VK0038 | 3 | 2 | 1 |
| VK0039 | 3 | 2 | 1 |
| VK0040 | 3 | 3 | 0 |
| VK0041 | 3 | 2 | 1 |
| VK0042 | 3 | 2 | 1 |
| VK0043 | 3 | 2 | 1 |
| VK0044 | 3 | 2 | 1 |
| VK0045 | 3 | 3 | 0 |
| VK0046 | 3 | 2 | 1 |
| VK0047 | 3 | 2 | 1 |
| VK0048 | 3 | 2 | 1 |
| VK0049 | 3 | 2 | 1 |
| VK0050 | 3 | 2 | 1 |
| VK0051 | 3 | 2 | 1 |
| VK0054 | 3 | 2 | 1 |
| VK0056 | 3 | 2 | 1 |
| VK0059 | 3 | 2 | 1 |
| VK0060 | 3 | 2 | 1 |
| VK0061 | 3 | 2 | 1 |
| VK0062 | 3 | 3 | 0 |
| VK0063 | 3 | 3 | 0 |
| VK0064 | 3 | 2 | 1 |
| VK0065 | 3 | 2 | 1 |
| VK0066 | 3 | 2 | 1 |
| VK0067 | 3 | 3 | 0 |
| VK0068 | 3 | 2 | 1 |
| VK0069 | 3 | 2 | 1 |
| VK0070 | 3 | 3 | 0 |
| VK0071 | 3 | 2 | 1 |
| VK0072 | 3 | 2 | 1 |
| VK0074 | 3 | 2 | 1 |
| VK0075 | 3 | 3 | 0 |
| VK0077 | 3 | 3 | 0 |
| VK0078 | 3 | 2 | 1 |
| VK0080 | 3 | 2 | 1 |
| VK0081 | 3 | 3 | 0 |
| VK0082 | 3 | 3 | 0 |
| VK0083 | 3 | 3 | 0 |
| VK0084 | 3 | 3 | 0 |
| VK0085 | 3 | 2 | 1 |
| VK0087 | 3 | 3 | 0 |
| VK0088 | 3 | 2 | 1 |
| VK0089 | 3 | 2 | 1 |
| VK0090 | 3 | 2 | 1 |
| VK0091 | 3 | 2 | 1 |
| VK0093 | 3 | 2 | 1 |
| VK0094 | 3 | 3 | 0 |
| VK0095 | 3 | 2 | 1 |
| VK0098 | 3 | 3 | 0 |
| VK0099 | 3 | 2 | 1 |
| VK0100 | 3 | 3 | 0 |
| VK0116 | 3 | 2 | 1 |
| VK0117 | 3 | 2 | 1 |
| VK0126 | 3 | 2 | 1 |
| VK0127 | 3 | 2 | 1 |
| VK0128 | 3 | 3 | 0 |
| VK0129 | 3 | 2 | 1 |
| VK0130 | 3 | 3 | 0 |
| VK0131 | 3 | 2 | 1 |
| VK0132 | 3 | 3 | 0 |
| VK0133 | 3 | 3 | 0 |
| VK0134 | 3 | 2 | 1 |
| VK0135 | 3 | 3 | 0 |
| VK0136 | 3 | 2 | 1 |
| VK0137 | 3 | 2 | 1 |
| VK0138 | 3 | 3 | 0 |
| VK0141 | 3 | 2 | 1 |
| VK0142 | 3 | 2 | 1 |
| VK0143 | 3 | 2 | 1 |
| VK0144 | 3 | 2 | 1 |
| VK0145 | 3 | 2 | 1 |
| VK0146 | 3 | 3 | 0 |
| VK0147 | 3 | 2 | 1 |
| VK0148 | 3 | 2 | 1 |
| VK0149 | 3 | 1 | 2 |
| VK0150 | 3 | 2 | 1 |
| VK0151 | 3 | 2 | 1 |
| VK0152 | 3 | 3 | 0 |
| VK0153 | 3 | 3 | 0 |
| VK0154 | 3 | 2 | 1 |
| VK0155 | 3 | 3 | 0 |
| VK0156 | 3 | 2 | 1 |
| VK0157 | 3 | 3 | 0 |
| VK0158 | 3 | 3 | 0 |
| VK0159 | 3 | 3 | 0 |
| VK0160 | 3 | 3 | 0 |
| VK0161 | 3 | 2 | 1 |
| VK0162 | 3 | 2 | 1 |
| VK0163 | 3 | 3 | 0 |
| VK0164 | 3 | 2 | 1 |
| VK0165 | 3 | 2 | 1 |
| VK0166 | 3 | 3 | 0 |
| VK0167 | 3 | 2 | 1 |
| VK0168 | 3 | 2 | 1 |
| VK0169 | 3 | 2 | 1 |
| VK0170 | 3 | 2 | 1 |
| VK0171 | 3 | 2 | 1 |
| VK0172 | 3 | 2 | 1 |
| VK0173 | 3 | 3 | 0 |
| VK0174 | 3 | 2 | 1 |
| VK0175 | 3 | 3 | 0 |
| VK0176 | 3 | 2 | 1 |
| VK0177 | 3 | 3 | 0 |
| VK0178 | 3 | 2 | 1 |
| VK0179 | 3 | 3 | 0 |
| VK0180 | 3 | 2 | 1 |
| VK0181 | 3 | 1 | 2 |
| VK0182 | 3 | 2 | 1 |
| VK0183 | 3 | 2 | 1 |
| VK0184 | 3 | 3 | 0 |
| VK0185 | 3 | 3 | 0 |
| VK0186 | 3 | 2 | 1 |
| VK0187 | 3 | 3 | 0 |
| VK0188 | 3 | 2 | 1 |
| VK0189 | 3 | 2 | 1 |
| VK0190 | 3 | 3 | 0 |
| VK0191 | 3 | 2 | 1 |
| VK0192 | 3 | 2 | 1 |
| VK0193 | 3 | 2 | 1 |
| VK0194 | 3 | 2 | 1 |
| VK0195 | 3 | 2 | 1 |
| VK0196 | 3 | 3 | 0 |
| VK0197 | 3 | 3 | 0 |
| VK0198 | 3 | 2 | 1 |
| VK0199 | 3 | 2 | 1 |
| VK0200 | 3 | 3 | 0 |
| VK0201 | 3 | 2 | 1 |
| VK0202 | 3 | 2 | 1 |
| VK0203 | 3 | 3 | 0 |
| VK0204 | 3 | 2 | 1 |
| VK0205 | 3 | 2 | 1 |
| VK0206 | 3 | 3 | 0 |
| VK0207 | 3 | 3 | 0 |
| VK0208 | 3 | 2 | 1 |
| VK0209 | 3 | 2 | 1 |
| VK0210 | 3 | 1 | 2 |
| VK0211 | 3 | 2 | 1 |
| VK0212 | 3 | 3 | 0 |
| VK0213 | 3 | 2 | 1 |
| VK0214 | 3 | 2 | 1 |
| VK0215 | 3 | 3 | 0 |
| VK0216 | 3 | 2 | 1 |
| VK0218 | 3 | 2 | 1 |
| VK0219 | 3 | 3 | 0 |
| VK0220 | 3 | 1 | 2 |
| VK0221 | 3 | 1 | 2 |
| VK0222 | 3 | 3 | 0 |
| VK0223 | 3 | 2 | 1 |
| VK0224 | 3 | 2 | 1 |
| VK0225 | 3 | 1 | 2 |
| VK0226 | 3 | 3 | 0 |
| VK0227 | 3 | 2 | 1 |
| VK0228 | 3 | 2 | 1 |
| VK0229 | 3 | 3 | 0 |
| VK0230 | 3 | 2 | 1 |
| VK0231 | 3 | 3 | 0 |
| VK0232 | 3 | 2 | 1 |
| VK0233 | 3 | 3 | 0 |
| VK0235 | 3 | 2 | 1 |
| VK0236 | 3 | 2 | 1 |
| VK0237 | 3 | 2 | 1 |
| VK0239 | 3 | 2 | 1 |
| VK0240 | 3 | 2 | 1 |
| VK0241 | 3 | 2 | 1 |
| VK0242 | 3 | 2 | 1 |
| VK0243 | 3 | 2 | 1 |
| VK0244 | 3 | 2 | 1 |
| VK0245 | 3 | 2 | 1 |
| VK0246 | 3 | 2 | 1 |
| VK0247 | 3 | 3 | 0 |
| VK0248 | 3 | 2 | 1 |
| VK0249 | 3 | 3 | 0 |
| VK0250 | 3 | 1 | 2 |
| VK0251 | 3 | 2 | 1 |
| VK0252 | 3 | 2 | 1 |
| VK0253 | 3 | 3 | 0 |
| VK0254 | 3 | 3 | 0 |
| VK0255 | 3 | 2 | 1 |
| VK0256 | 3 | 3 | 0 |
| VK0257 | 3 | 2 | 1 |
| VK0258 | 3 | 2 | 1 |
| VK0259 | 3 | 2 | 1 |
| VK0260 | 3 | 3 | 0 |
| VK0261 | 3 | 3 | 0 |
| VK0262 | 3 | 2 | 1 |
| VK0263 | 3 | 2 | 1 |
| VK0264 | 3 | 2 | 1 |
| VK0265 | 3 | 2 | 1 |
| VK0266 | 3 | 2 | 1 |
| VK0267 | 3 | 2 | 1 |
| VK0268 | 3 | 2 | 1 |
| VK0269 | 3 | 2 | 1 |
| VK0270 | 3 | 3 | 0 |
| VK0271 | 3 | 2 | 1 |
| VK0272 | 3 | 2 | 1 |
| VK0273 | 3 | 2 | 1 |
| VK0274 | 3 | 2 | 1 |
| VK0275 | 3 | 2 | 1 |
| VK0276 | 3 | 2 | 1 |
| VK0277 | 3 | 2 | 1 |
| VK0278 | 3 | 2 | 1 |
| VK0280 | 3 | 3 | 0 |
| VK0281 | 3 | 2 | 1 |
| VK0283 | 3 | 2 | 1 |
| VK0284 | 3 | 2 | 1 |
| VK0285 | 3 | 2 | 1 |
| VK0286 | 3 | 1 | 2 |
| VK0289 | 3 | 2 | 1 |
| VK0303 | 3 | 2 | 1 |
| VK0307 | 3 | 2 | 1 |
| VK0308 | 3 | 3 | 0 |
| VK0309 | 3 | 3 | 0 |
| VK0310 | 3 | 2 | 1 |
| VK0311 | 3 | 2 | 1 |
| VK0312 | 3 | 2 | 1 |
| VK0313 | 3 | 2 | 1 |
| VK0315 | 3 | 2 | 1 |
| VK0317 | 3 | 2 | 1 |
| VK0318 | 3 | 2 | 1 |
| VK0319 | 3 | 2 | 1 |
| VK0320 | 3 | 3 | 0 |
| VK0321 | 3 | 2 | 1 |
| VK0322 | 3 | 2 | 1 |
| VK0323 | 3 | 2 | 1 |
| VK0324 | 3 | 2 | 1 |
| VK0325 | 3 | 2 | 1 |
| VK0326 | 3 | 3 | 0 |
| VK0327 | 3 | 2 | 1 |
| VK0328 | 3 | 1 | 2 |
| VK0329 | 3 | 2 | 1 |
| VK0331 | 3 | 2 | 1 |
| VK0332 | 3 | 1 | 2 |
| VK0334 | 3 | 2 | 1 |
| VK0335 | 3 | 2 | 1 |
| VK0336 | 3 | 2 | 1 |
| VK0337 | 3 | 3 | 0 |
| VK0338 | 3 | 3 | 0 |
| VK0340 | 3 | 3 | 0 |
| VK0341 | 3 | 2 | 1 |
| VK0342 | 3 | 2 | 1 |
| VK0343 | 3 | 2 | 1 |
| VK0345 | 3 | 2 | 1 |
| VK0349 | 3 | 2 | 1 |
| VK0352 | 3 | 2 | 1 |
| VK0353 | 3 | 2 | 1 |
| VK0354 | 3 | 2 | 1 |
| VK0355 | 3 | 2 | 1 |
| VK0356 | 3 | 2 | 1 |
| VK0357 | 3 | 1 | 2 |
| VK0358 | 3 | 2 | 1 |
| VK0359 | 3 | 3 | 0 |
| VK0360 | 3 | 2 | 1 |
| VK0361 | 3 | 2 | 1 |
| VK0362 | 3 | 2 | 1 |
| VK0363 | 3 | 2 | 1 |
| VK0364 | 3 | 3 | 0 |
| VK0365 | 3 | 2 | 1 |
| VK0366 | 3 | 2 | 1 |
| VK0367 | 3 | 2 | 1 |
| VK0368 | 3 | 2 | 1 |
| VK0369 | 3 | 2 | 1 |
| VK0370 | 3 | 2 | 1 |
| VK0371 | 3 | 2 | 1 |
| VK0372 | 3 | 2 | 1 |
| VK0373 | 3 | 3 | 0 |
| VK0378 | 3 | 1 | 2 |
| VK0379 | 3 | 1 | 2 |
| VK0382 | 3 | 2 | 1 |
| VK0384 | 3 | 2 | 1 |
| VK0386 | 3 | 2 | 1 |
| VK0387 | 3 | 3 | 0 |
| VK0388 | 3 | 2 | 1 |
| VK0389 | 3 | 1 | 2 |
| VK0390 | 3 | 1 | 2 |
| VK0391 | 3 | 2 | 1 |
| VK0392 | 3 | 3 | 0 |
| VK0393 | 3 | 2 | 1 |
| VK0395 | 3 | 1 | 2 |
| VK0397 | 3 | 2 | 1 |
| VK0398 | 3 | 2 | 1 |
| VK0399 | 3 | 2 | 1 |
| VK0400 | 3 | 2 | 1 |
| VK0401 | 3 | 3 | 0 |
| VK0402 | 3 | 2 | 1 |
| VK0403 | 3 | 3 | 0 |
| VK0404 | 3 | 2 | 1 |
| VK0405 | 3 | 2 | 1 |
| VK0406 | 3 | 3 | 0 |
| VK0407 | 3 | 2 | 1 |
| VK0408 | 3 | 3 | 0 |
| VK0409 | 3 | 2 | 1 |
| VK0410 | 3 | 2 | 1 |
| VK0411 | 3 | 2 | 1 |
| VK0412 | 3 | 3 | 0 |
| VK0413 | 3 | 2 | 1 |
| VK0414 | 3 | 2 | 1 |
| VK0415 | 3 | 3 | 0 |
| VK0416 | 3 | 2 | 1 |
| VK0417 | 3 | 2 | 1 |
| VK0418 | 3 | 2 | 1 |
| VK0419 | 3 | 3 | 0 |
| VK0420 | 3 | 3 | 0 |
| VK0421 | 3 | 2 | 1 |
| VK0422 | 3 | 2 | 1 |
| VK0423 | 3 | 2 | 1 |
| VK0424 | 3 | 3 | 0 |
| VK0425 | 3 | 2 | 1 |
| VK0426 | 3 | 3 | 0 |
| VK0427 | 3 | 3 | 0 |
| VK0428 | 3 | 2 | 1 |
| VK0430 | 3 | 3 | 0 |
| VK0432 | 3 | 1 | 2 |
| VK0433 | 3 | 2 | 1 |
| VK0434 | 3 | 2 | 1 |
| VK0435 | 3 | 2 | 1 |
| VK0437 | 3 | 2 | 1 |
| VK0438 | 3 | 3 | 0 |
| VK0439 | 3 | 3 | 0 |
| VK0440 | 3 | 2 | 1 |
| VK0441 | 3 | 3 | 0 |
| VK0442 | 3 | 2 | 1 |
| VK0443 | 3 | 2 | 1 |
| VK0444 | 3 | 2 | 1 |
| VK0445 | 3 | 3 | 0 |
| VK0446 | 3 | 2 | 1 |
| VK0447 | 3 | 2 | 1 |
| VK0449 | 3 | 2 | 1 |
| VK0450 | 3 | 2 | 1 |
| VK0451 | 3 | 3 | 0 |
| VK0452 | 3 | 2 | 1 |
| VK0453 | 3 | 2 | 1 |
| VK0454 | 3 | 3 | 0 |
| VK0455 | 3 | 2 | 1 |
| VK0456 | 3 | 2 | 1 |
| VK0457 | 3 | 2 | 1 |
| VK0459 | 3 | 2 | 1 |
| VK0460 | 3 | 2 | 1 |
| VK0463 | 3 | 2 | 1 |
| VK0465 | 3 | 3 | 0 |
| VK0467 | 3 | 2 | 1 |
| VK0468 | 3 | 3 | 0 |
| VK0469 | 3 | 2 | 1 |
| VK0470 | 3 | 1 | 2 |
| VK0471 | 3 | 3 | 0 |
| VK0472 | 3 | 3 | 0 |
| VK0476 | 3 | 3 | 0 |
| VK0477 | 3 | 2 | 1 |
| VK0478 | 3 | 2 | 1 |
| VK0480 | 3 | 2 | 1 |
| VK0481 | 3 | 3 | 0 |
| VK0483 | 3 | 2 | 1 |
| VK0484 | 3 | 2 | 1 |
| VK0485 | 3 | 3 | 0 |
| VK0486 | 3 | 3 | 0 |
| VK0487 | 3 | 2 | 1 |
| VK0488 | 3 | 2 | 1 |
| VK0489 | 3 | 2 | 1 |
| VK0490 | 3 | 2 | 1 |
| VK0491 | 3 | 3 | 0 |
| VK0492 | 3 | 2 | 1 |
| VK0493 | 3 | 2 | 1 |
| VK0494 | 3 | 2 | 1 |
| VK0495 | 3 | 2 | 1 |
| VK0496 | 3 | 2 | 1 |
| VK0497 | 3 | 2 | 1 |
| VK0498 | 3 | 2 | 1 |
| VK0499 | 3 | 2 | 1 |
| VK0500 | 3 | 2 | 1 |
| VK0501 | 3 | 3 | 0 |
| VK0502 | 3 | 2 | 1 |
| VK0503 | 3 | 1 | 2 |
| VK0504 | 3 | 3 | 0 |
| VK0505 | 3 | 1 | 2 |
| VK0506 | 3 | 2 | 1 |
| VK0507 | 3 | 1 | 2 |
| VK0508 | 3 | 1 | 2 |
| VK0510 | 3 | 2 | 1 |
| VK0512 | 3 | 2 | 1 |
| VK0514 | 3 | 2 | 1 |
| VK0515 | 3 | 2 | 1 |
| VK0516 | 3 | 2 | 1 |
| VK0517 | 3 | 2 | 1 |
| VK0518 | 3 | 2 | 1 |
| VK0519 | 3 | 3 | 0 |
| VK0520 | 3 | 2 | 1 |
| VK0521 | 3 | 2 | 1 |
| VK0523 | 3 | 2 | 1 |
| VK0524 | 3 | 2 | 1 |
| VK0525 | 3 | 2 | 1 |
| VK0526 | 3 | 2 | 1 |
| VK0527 | 3 | 3 | 0 |
| VK0528 | 3 | 2 | 1 |
| VK0529 | 3 | 3 | 0 |
| VK0530 | 3 | 3 | 0 |
| VK0531 | 3 | 3 | 0 |
| VK0533 | 3 | 3 | 0 |
| VK0534 | 3 | 2 | 1 |
| VK0535 | 3 | 3 | 0 |
| VK0537 | 3 | 2 | 1 |
| VK0539 | 3 | 2 | 1 |
| VK0540 | 3 | 2 | 1 |
| VK0541 | 3 | 2 | 1 |
| VK0543 | 3 | 2 | 1 |
| VK0544 | 3 | 3 | 0 |
| VK0546 | 3 | 3 | 0 |
| VK0547 | 3 | 2 | 1 |
| VK0548 | 3 | 2 | 1 |
| VK0550 | 3 | 2 | 1 |
| VK0551 | 3 | 3 | 0 |
| VK0552 | 3 | 2 | 1 |
| VK0553 | 3 | 2 | 1 |
| VK0554 | 3 | 2 | 1 |
| VK0555 | 3 | 2 | 1 |
| VK0556 | 3 | 1 | 2 |
| VK0557 | 3 | 3 | 0 |
| VK0558 | 3 | 3 | 0 |
| VK0559 | 3 | 2 | 1 |
| VK0560 | 3 | 2 | 1 |
| VK0561 | 3 | 3 | 0 |
| VK0563 | 3 | 2 | 1 |
| VK0564 | 3 | 2 | 1 |
| VK0565 | 3 | 2 | 1 |
| VK0566 | 3 | 2 | 1 |
| VK0567 | 3 | 2 | 1 |
| VK0568 | 3 | 2 | 1 |
| VK0569 | 3 | 2 | 1 |
| VK0570 | 3 | 3 | 0 |
| VK0571 | 3 | 2 | 1 |
| VK0572 | 3 | 2 | 1 |
| VK0573 | 3 | 2 | 1 |
| VK0574 | 3 | 2 | 1 |
| VK0575 | 3 | 2 | 1 |
| VK0576 | 3 | 2 | 1 |
| VK0577 | 3 | 2 | 1 |
| VK0578 | 3 | 3 | 0 |
| VK0579 | 3 | 2 | 1 |
| VK0580 | 3 | 2 | 1 |
| VK0581 | 3 | 1 | 2 |
| VK0582 | 3 | 3 | 0 |
| VK0583 | 3 | 2 | 1 |
| VK0586 | 3 | 2 | 1 |
| VK0595 | 3 | 2 | 1 |
| VK0596 | 3 | 2 | 1 |
| VK0597 | 3 | 2 | 1 |
| VK0598 | 3 | 2 | 1 |
| VK0599 | 3 | 2 | 1 |
| VK0603 | 3 | 3 | 0 |
| VK0604 | 3 | 2 | 1 |
| VK0605 | 3 | 2 | 1 |
| VK0606 | 3 | 2 | 1 |
| VK0607 | 3 | 3 | 0 |
| VK0608 | 3 | 2 | 1 |
| VK0609 | 3 | 3 | 0 |
| VK0610 | 3 | 2 | 1 |
| VK0611 | 3 | 3 | 0 |
| VK0612 | 3 | 3 | 0 |
| VK0613 | 3 | 2 | 1 |
| VK0614 | 3 | 3 | 0 |
| VK0615 | 3 | 3 | 0 |
| VK0616 | 3 | 2 | 1 |
| VK0618 | 3 | 2 | 1 |
| VK0619 | 3 | 2 | 1 |
| VK0620 | 3 | 2 | 1 |
| VK0621 | 3 | 3 | 0 |
| VK0622 | 3 | 2 | 1 |
| VK0623 | 3 | 2 | 1 |
| VK0625 | 3 | 2 | 1 |
| VK0626 | 3 | 2 | 1 |
| VK0627 | 3 | 2 | 1 |
| VK0628 | 3 | 3 | 0 |
| VK0634 | 3 | 2 | 1 |
| VK0635 | 3 | 2 | 1 |
| VK0636 | 3 | 2 | 1 |
| VK0637 | 3 | 2 | 1 |
| VK0638 | 3 | 3 | 0 |
| VK0640 | 3 | 2 | 1 |
| VK0641 | 3 | 2 | 1 |
| VK0642 | 3 | 3 | 0 |
| VK0644 | 3 | 3 | 0 |
| VK0645 | 3 | 2 | 1 |
| VK0646 | 3 | 3 | 0 |
| VK0647 | 3 | 3 | 0 |
| VK0650 | 3 | 2 | 1 |
| VK0651 | 3 | 1 | 2 |
| VK0652 | 3 | 2 | 1 |
| VK0654 | 3 | 2 | 1 |
| VK0655 | 3 | 3 | 0 |
| VK0656 | 3 | 2 | 1 |
| VK0657 | 3 | 3 | 0 |
| VK0660 | 3 | 2 | 1 |
| VK0663 | 3 | 2 | 1 |
| VK0664 | 3 | 2 | 1 |
| VK0665 | 3 | 1 | 2 |
| VK0668 | 3 | 2 | 1 |
| VK0670 | 3 | 3 | 0 |
| VK0672 | 3 | 2 | 1 |
| VK0673 | 3 | 2 | 1 |
| VK0674 | 3 | 3 | 0 |
| VK0677 | 3 | 2 | 1 |
| VK0678 | 3 | 2 | 1 |
| VK0680 | 3 | 1 | 2 |
| VK0681 | 3 | 3 | 0 |
| VK0682 | 3 | 1 | 2 |
| VK0683 | 3 | 3 | 0 |
| VK0684 | 3 | 2 | 1 |
| VK0685 | 3 | 2 | 1 |
| VK0686 | 3 | 2 | 1 |
| VK0687 | 3 | 2 | 1 |
| VK0688 | 3 | 2 | 1 |
| VK0689 | 3 | 3 | 0 |
| VK0690 | 3 | 2 | 1 |
| VK0692 | 3 | 3 | 0 |
| VK0693 | 3 | 2 | 1 |
| VK0694 | 3 | 2 | 1 |
| VK0695 | 3 | 2 | 1 |
| VK0696 | 3 | 2 | 1 |
| VK0697 | 3 | 3 | 0 |
| VK0698 | 3 | 2 | 1 |
| VK0699 | 3 | 2 | 1 |
| VK0700 | 3 | 2 | 1 |
| VK0701 | 3 | 2 | 1 |
| VK0702 | 3 | 2 | 1 |
| VK0703 | 3 | 2 | 1 |
| VK0704 | 3 | 3 | 0 |
| VK0705 | 3 | 1 | 2 |
| VK0706 | 3 | 3 | 0 |
| VK0707 | 3 | 2 | 1 |
| VK0708 | 3 | 2 | 1 |
| VK0709 | 3 | 2 | 1 |
| VK0710 | 3 | 2 | 1 |
| VK0711 | 3 | 2 | 1 |
| VK0712 | 3 | 2 | 1 |
| VK0713 | 3 | 3 | 0 |
| VK0715 | 3 | 2 | 1 |
| VK0716 | 3 | 2 | 1 |
| VK0717 | 3 | 3 | 0 |
| VK0718 | 3 | 3 | 0 |
| VK0719 | 3 | 2 | 1 |
| VK0721 | 3 | 2 | 1 |
| VK0722 | 3 | 2 | 1 |
| VK0723 | 3 | 2 | 1 |
| VK0724 | 3 | 2 | 1 |
| VK0725 | 3 | 1 | 2 |
| VK0726 | 3 | 3 | 0 |
| VK0727 | 3 | 2 | 1 |
| VK0728 | 3 | 1 | 2 |
| VK0729 | 3 | 2 | 1 |
| VK0735 | 3 | 3 | 0 |
| VK0736 | 3 | 3 | 0 |
| VK0737 | 3 | 3 | 0 |
| VK0738 | 3 | 3 | 0 |
| VK0739 | 3 | 3 | 0 |
| VK0740 | 3 | 3 | 0 |
| VK0741 | 3 | 2 | 1 |
| VK0742 | 3 | 2 | 1 |
| VK0743 | 3 | 3 | 0 |
| VK0744 | 3 | 3 | 0 |
| VK0745 | 3 | 3 | 0 |
| VK0746 | 3 | 3 | 0 |
| VK0747 | 3 | 3 | 0 |
| VK0748 | 3 | 3 | 0 |
