# Supplementary material for: Phylogenomic and mitogenomic data can accelerate inventorying of tropical beetles during the current biodiversity crisis
Source: eLife. 2021 Dec 20;10:e71895. doi: 10.7554/eLife.71895 (PMC8798050; doi:10.7554/eLife.71895)
Supplement: Source data 1. — Depicted numbers above branches represent SH-alrt support values. [file elife-71895-data1.pdf]

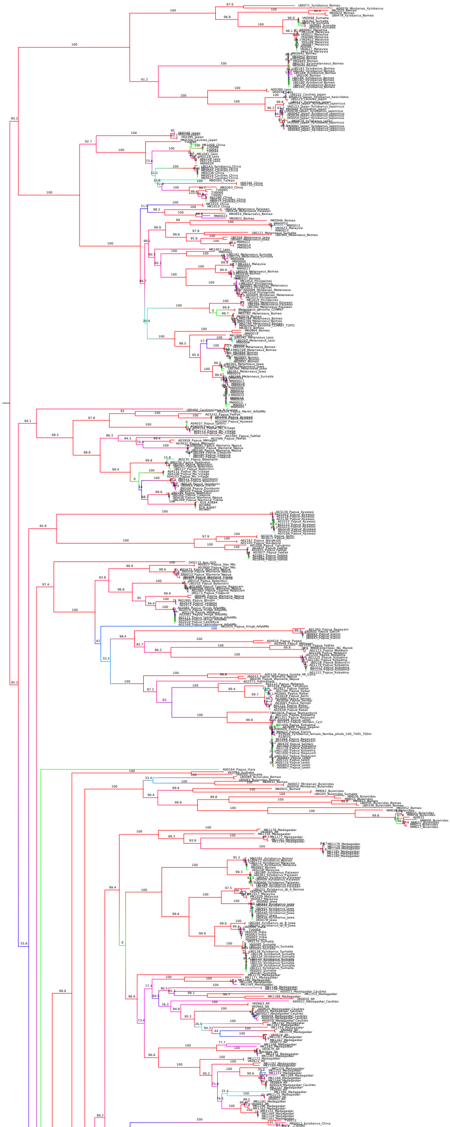

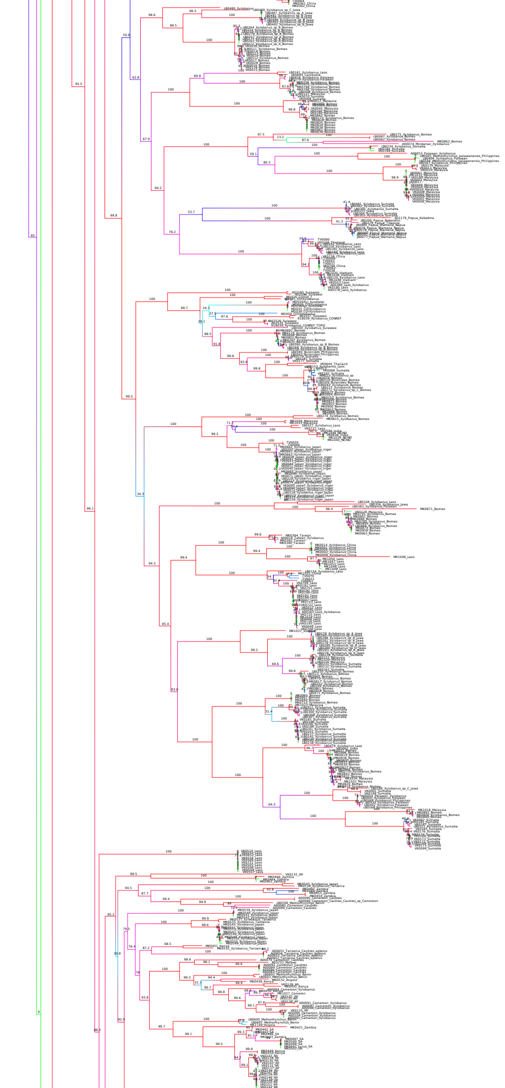

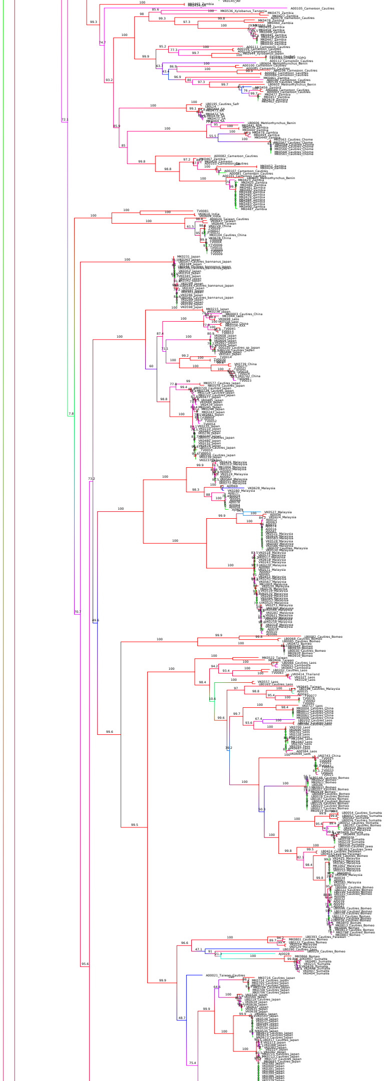

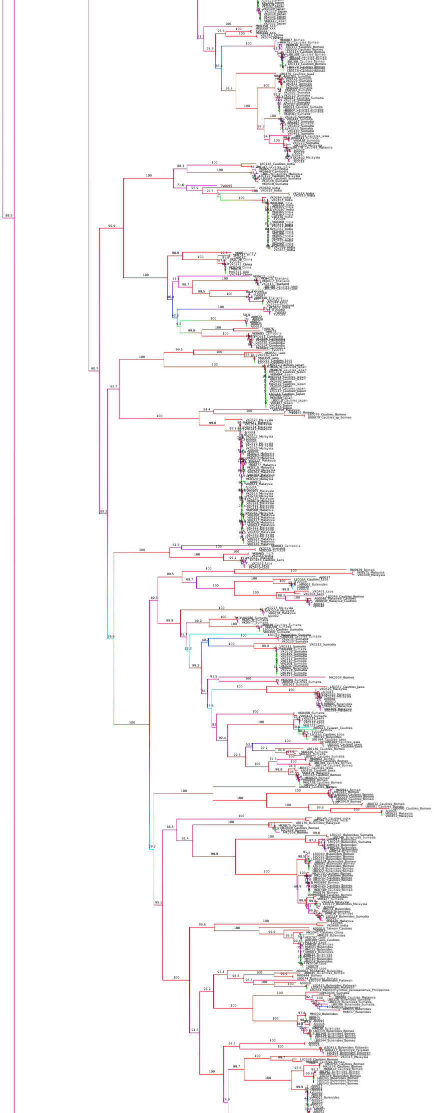

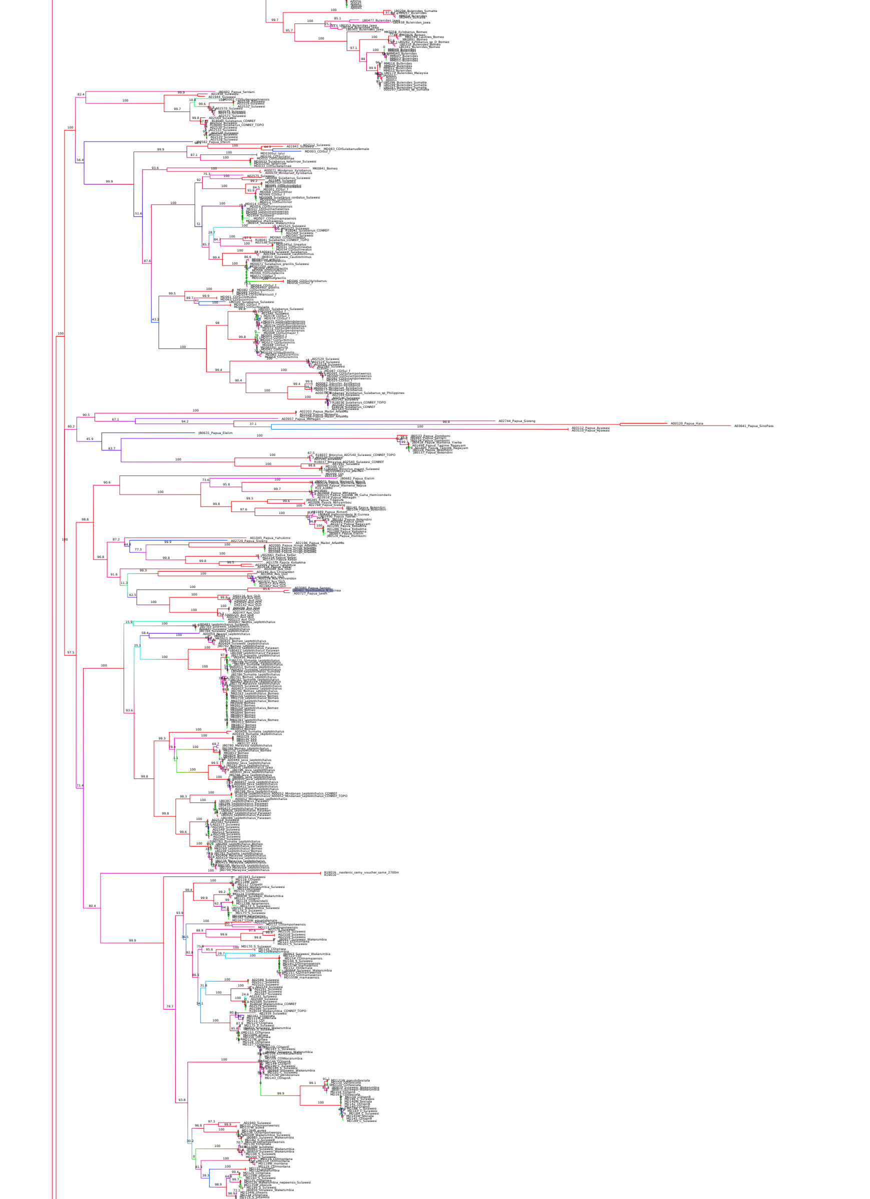

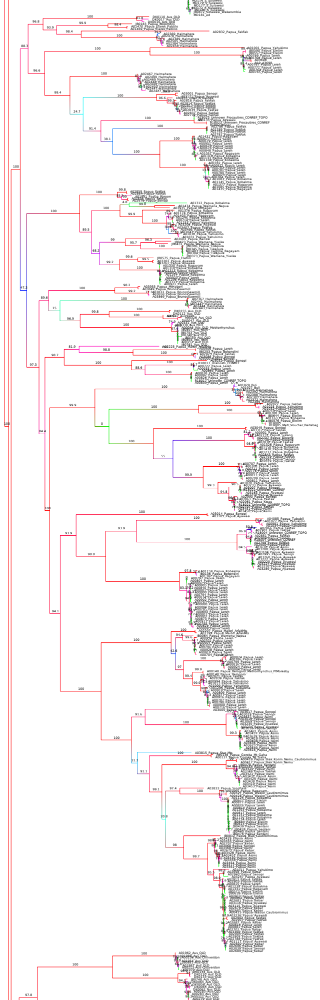

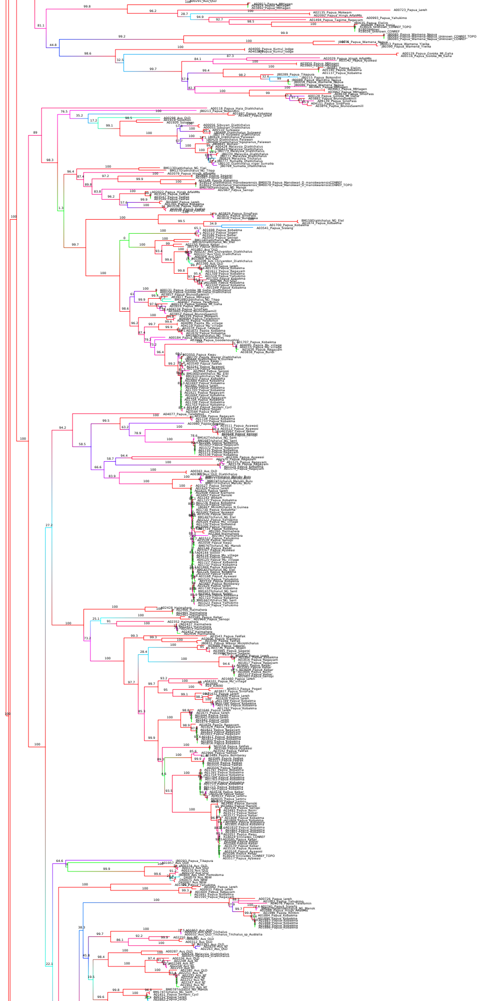

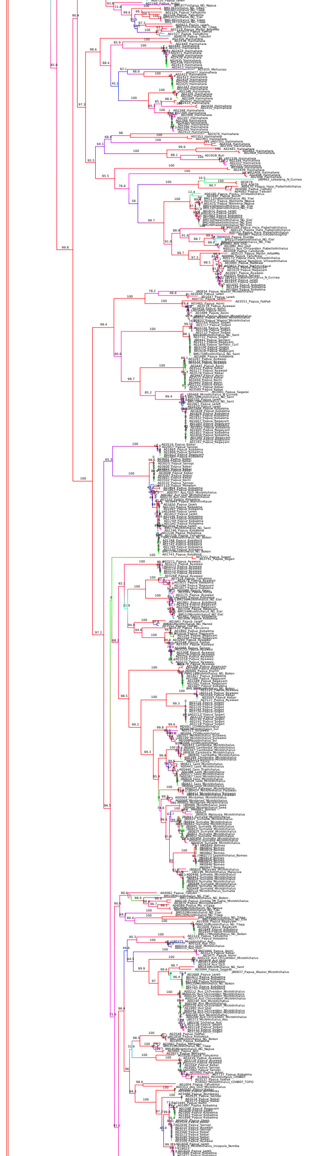

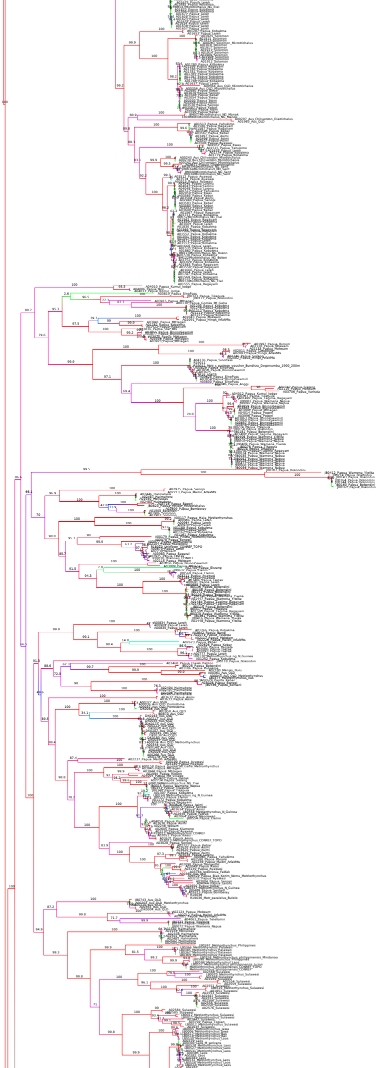

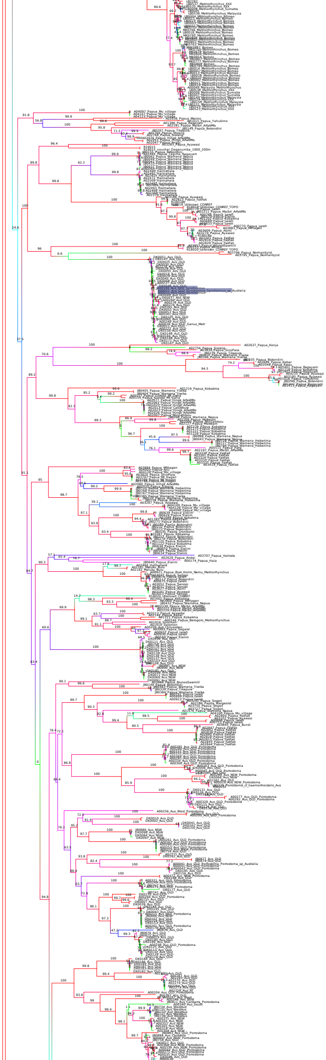

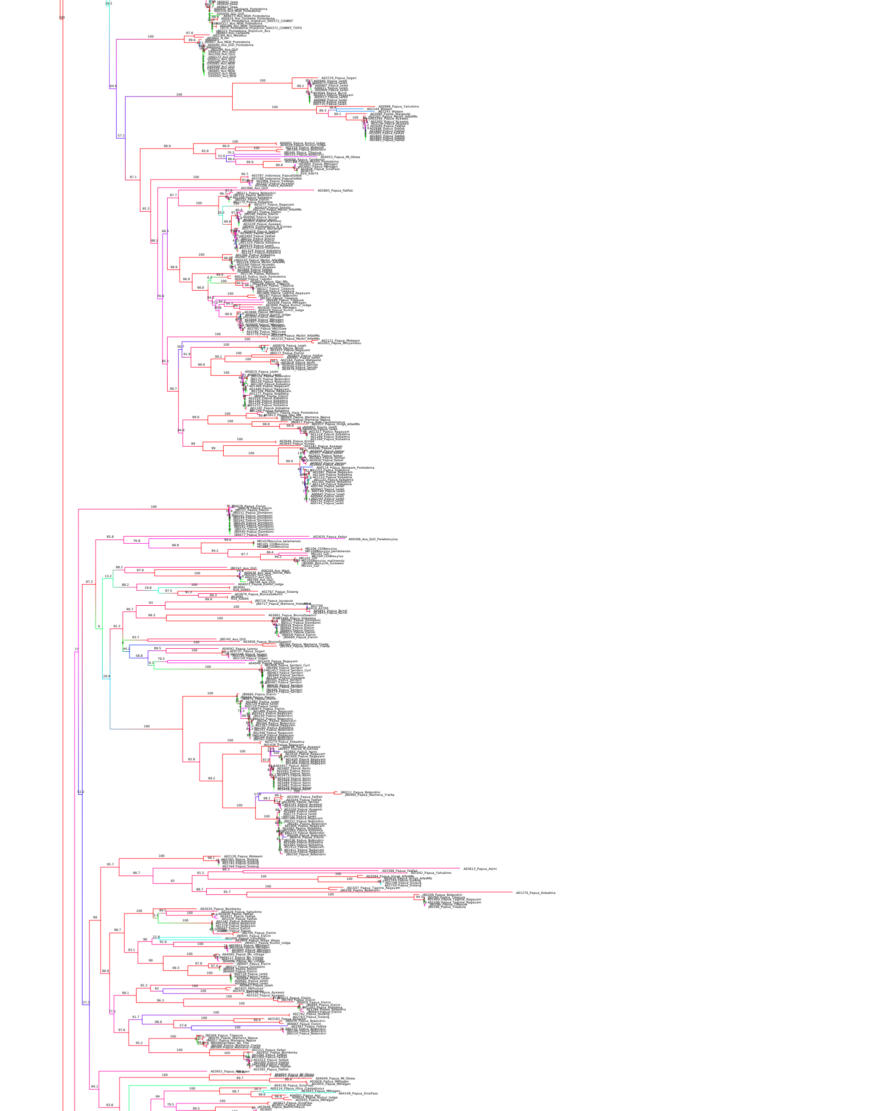

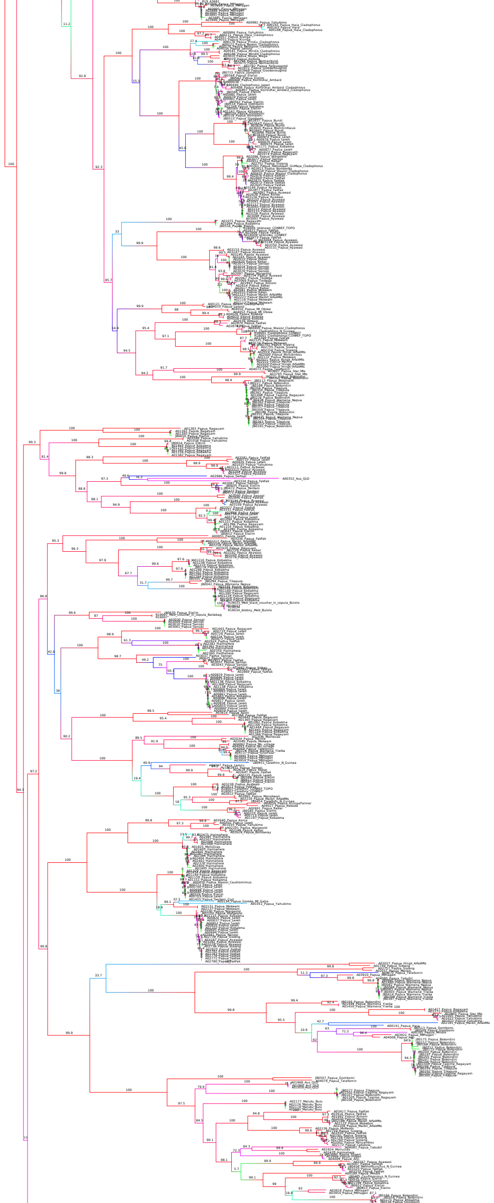

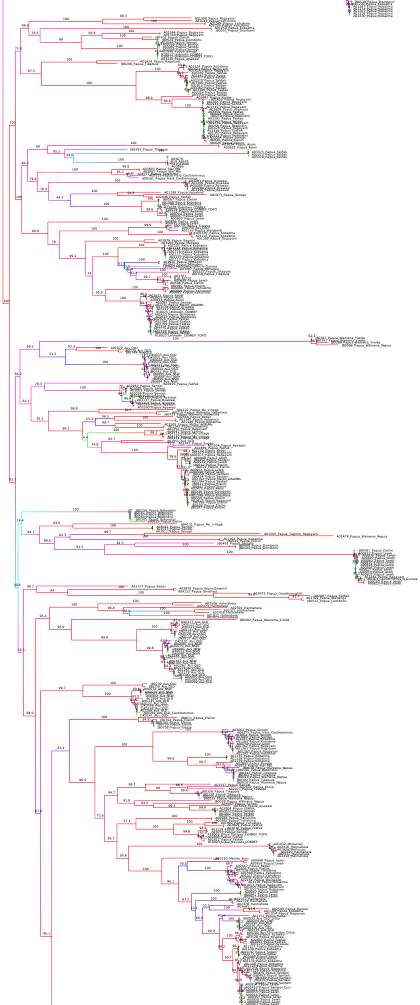

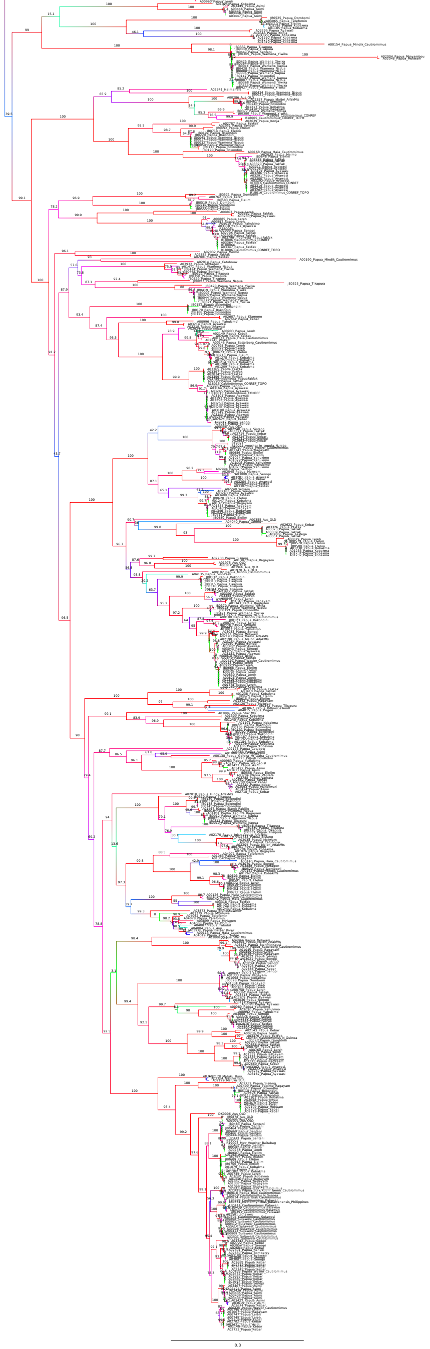
0.3
