# Supplementary material for: Phylogenomic and mitogenomic data can accelerate inventorying of tropical beetles during the current biodiversity crisis
Source: eLife. 2021 Dec 20;10:e71895. doi: 10.7554/eLife.71895 (PMC8798050; doi:10.7554/eLife.71895)
Supplement: Source data 2. — Depicted numbers above branches represent SH-aLRT support values. The taxa with the constrained position in the tree are marked with the red star. [file elife-71895-data2.pdf]

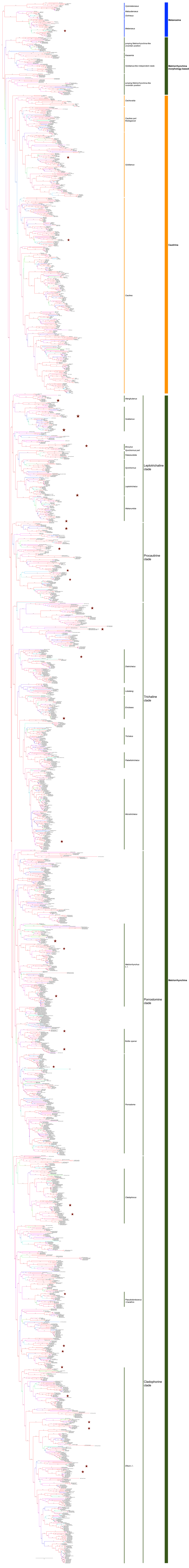

Xylometanoeus
Matsudanoeus
Ochineus
Metanoeus
Metanoeina
jumping Metriorrhynchina-like uncertain position
Kassemia
Xylobanus-like independent clade
jumping Metriorrhynchina-like uncertain position
Metriorrhynchina morphology-based
Cechovskia
Cautires part Madagascar
Xylobanus
Cautires
Cautirina
Mangkutanus
Sulabanus
Broxylus
Synchonnus part
Falsolucidota
Synchonnus
Leptotrichalus
Wakarumbia
Leptotrichaline clade
Procautirine clade
Diatrichalus
Lobatang
Eniclases
Trichalus
Flabellotrichalus
Microtrichalus
Trichaline clade
Metriorrhynchus s. l.
Bottle opener
Porrostoma
Porrostomine clade
Metriorrhynchina
Cladophorus
Pseudodontocerus =Carathrix
Ditua s. l.
Cladophorine clade
